# Supplementary material for: LncEGFL7OS regulates human angiogenesis by interacting with MAX at the EGFL7/miR-126 locus
Source: eLife. 2019 Feb 11;8:e40470. doi: 10.7554/eLife.40470 (PMC6370342; doi:10.7554/eLife.40470)
Supplement: Figure 1—source data 1. [file elife-40470-fig1-data1.pptx]

## Slide 1
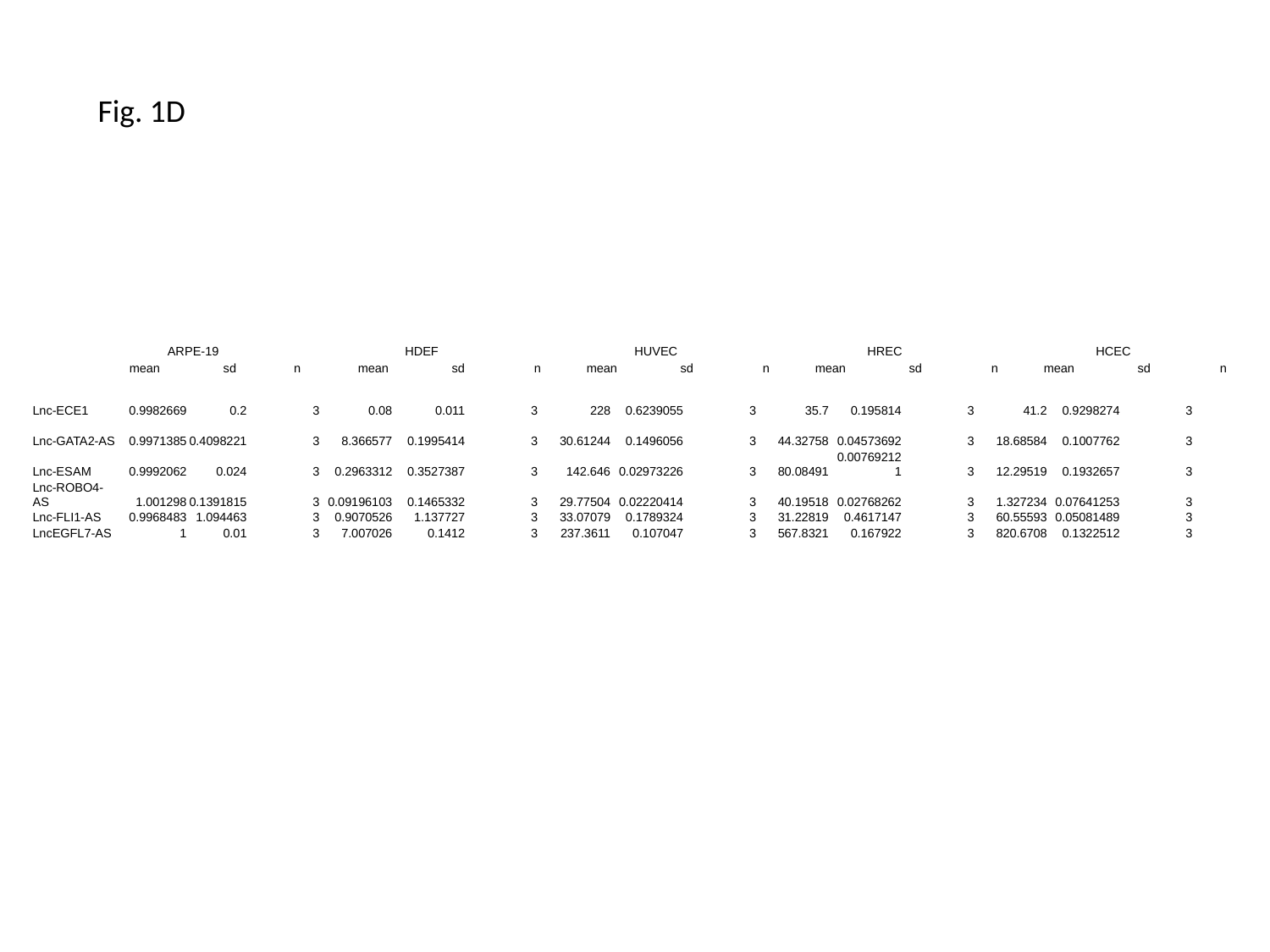

Fig. 1D
| ARPE-19 | | | HDEF | | | HUVEC | | | HREC | | | HCEC | | |
| --- | --- | --- | --- | --- | --- | --- | --- | --- | --- | --- | --- | --- | --- | --- |
| mean | sd | n | mean | sd | n | mean | sd | n | mean | sd | n | mean | sd | n |
| Lnc-ECE1 | 0.9982669 | 0.2 | 3 | 0.08 | 0.011 | 3 | 228 | 0.6239055 | 3 | 35.7 | 0.195814 | 3 | 41.2 | 0.9298274 | 3 |
| --- | --- | --- | --- | --- | --- | --- | --- | --- | --- | --- | --- | --- | --- | --- | --- |
| Lnc-GATA2-AS | 0.9971385 | 0.4098221 | 3 | 8.366577 | 0.1995414 | 3 | 30.61244 | 0.1496056 | 3 | 44.32758 | 0.04573692 | 3 | 18.68584 | 0.1007762 | 3 |
| Lnc-ESAM | 0.9992062 | 0.024 | 3 | 0.2963312 | 0.3527387 | 3 | 142.646 | 0.02973226 | 3 | 80.08491 | 0.007692121 | 3 | 12.29519 | 0.1932657 | 3 |
| Lnc-ROBO4-AS | 1.001298 | 0.1391815 | 3 | 0.09196103 | 0.1465332 | 3 | 29.77504 | 0.02220414 | 3 | 40.19518 | 0.02768262 | 3 | 1.327234 | 0.07641253 | 3 |
| Lnc-FLI1-AS | 0.9968483 | 1.094463 | 3 | 0.9070526 | 1.137727 | 3 | 33.07079 | 0.1789324 | 3 | 31.22819 | 0.4617147 | 3 | 60.55593 | 0.05081489 | 3 |
| LncEGFL7-AS | 1 | 0.01 | 3 | 7.007026 | 0.1412 | 3 | 237.3611 | 0.107047 | 3 | 567.8321 | 0.167922 | 3 | 820.6708 | 0.1322512 | 3 |
