# Supplementary material for: LncEGFL7OS regulates human angiogenesis by interacting with MAX at the EGFL7/miR-126 locus
Source: eLife. 2019 Feb 11;8:e40470. doi: 10.7554/eLife.40470 (PMC6370342; doi:10.7554/eLife.40470)
Supplement: Figure 2—source data 1. [file elife-40470-fig2-data1.pptx]

## Slide 1
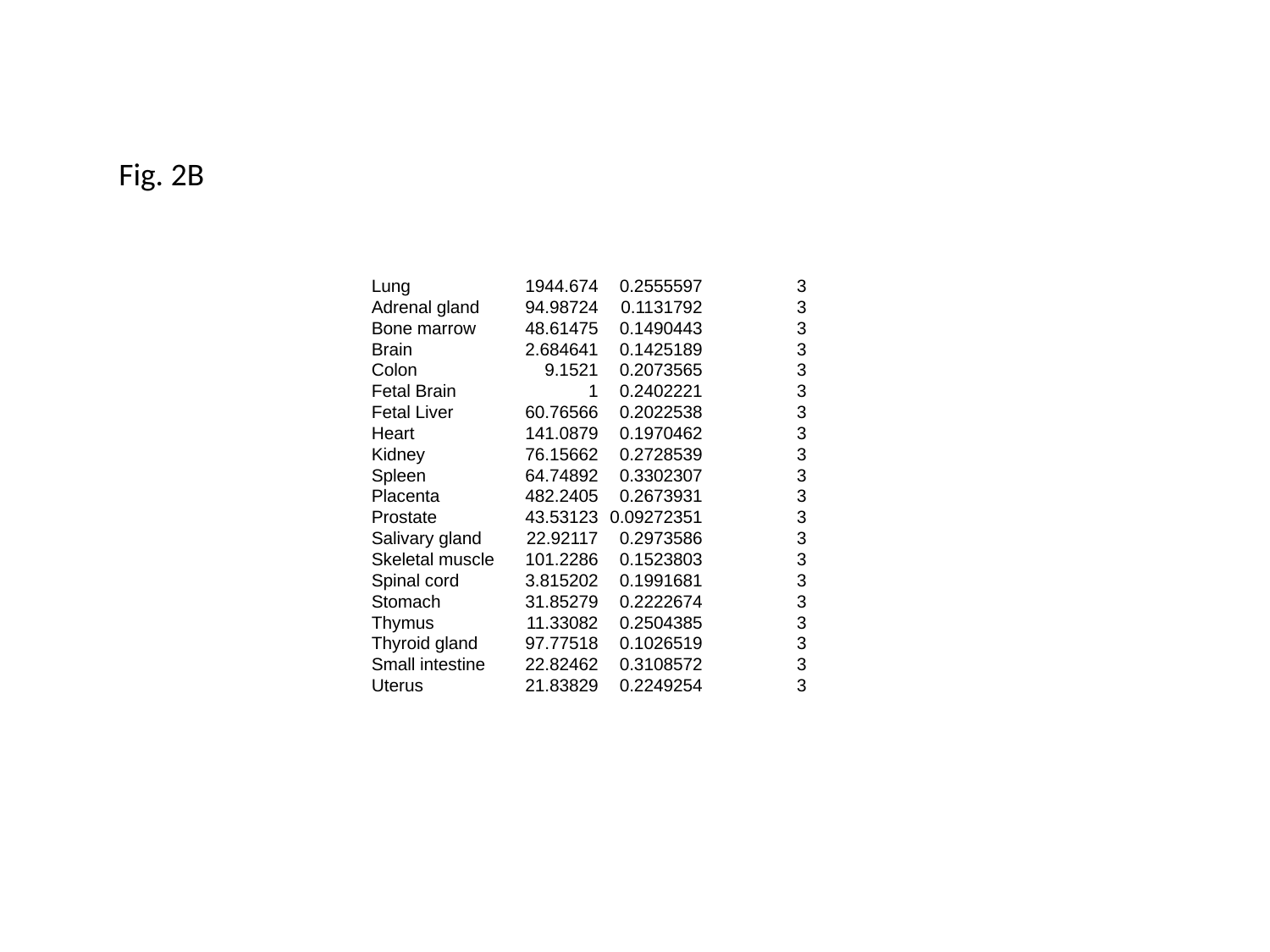

Fig. 2B
| Lung | 1944.674 | 0.2555597 | 3 |
| --- | --- | --- | --- |
| Adrenal gland | 94.98724 | 0.1131792 | 3 |
| Bone marrow | 48.61475 | 0.1490443 | 3 |
| Brain | 2.684641 | 0.1425189 | 3 |
| Colon | 9.1521 | 0.2073565 | 3 |
| Fetal Brain | 1 | 0.2402221 | 3 |
| Fetal Liver | 60.76566 | 0.2022538 | 3 |
| Heart | 141.0879 | 0.1970462 | 3 |
| Kidney | 76.15662 | 0.2728539 | 3 |
| Spleen | 64.74892 | 0.3302307 | 3 |
| Placenta | 482.2405 | 0.2673931 | 3 |
| Prostate | 43.53123 | 0.09272351 | 3 |
| Salivary gland | 22.92117 | 0.2973586 | 3 |
| Skeletal muscle | 101.2286 | 0.1523803 | 3 |
| Spinal cord | 3.815202 | 0.1991681 | 3 |
| Stomach | 31.85279 | 0.2222674 | 3 |
| Thymus | 11.33082 | 0.2504385 | 3 |
| Thyroid gland | 97.77518 | 0.1026519 | 3 |
| Small intestine | 22.82462 | 0.3108572 | 3 |
| Uterus | 21.83829 | 0.2249254 | 3 |

## Slide 2
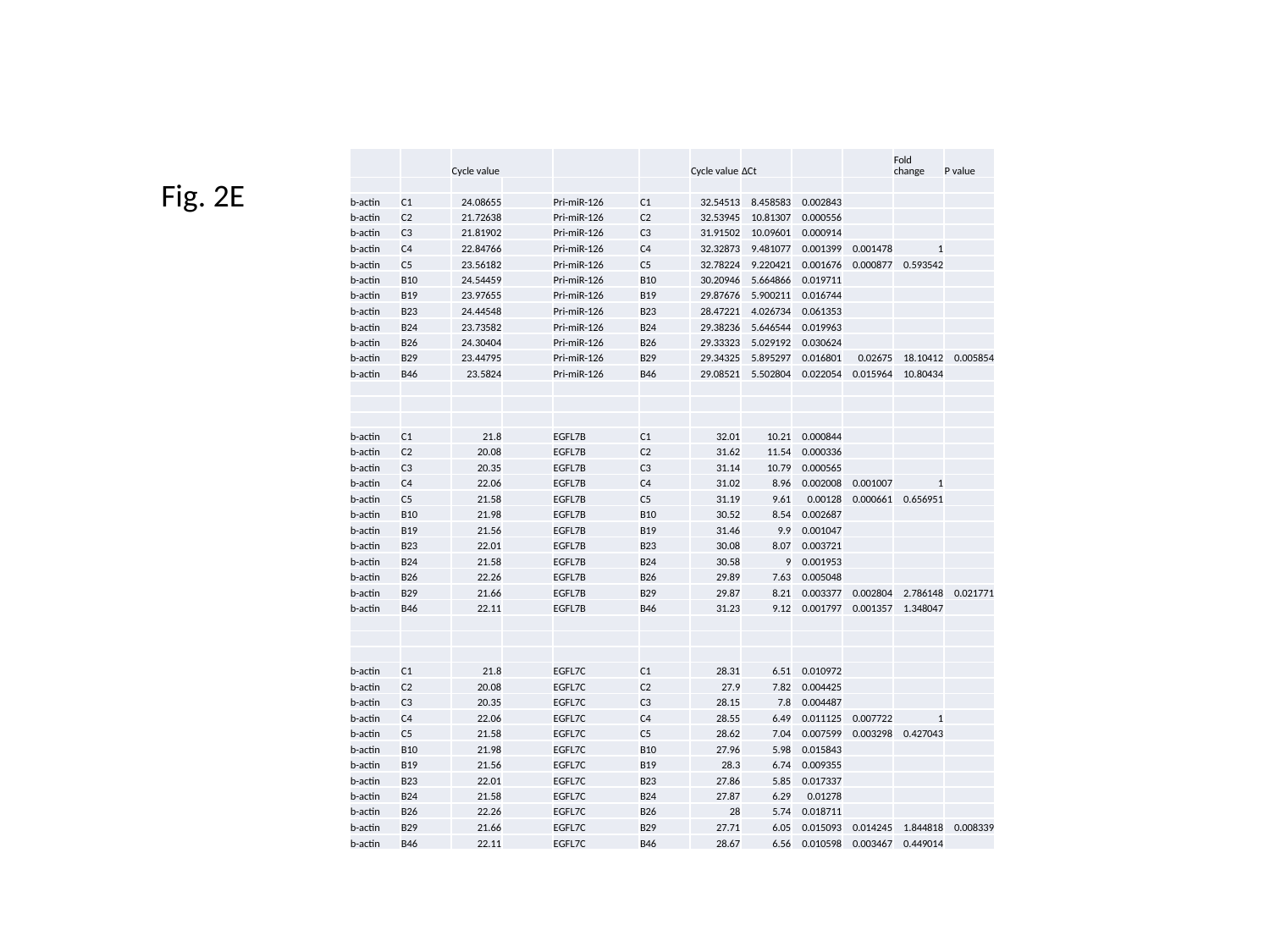

| | | Cycle value | | | | Cycle value | ΔCt | | | Fold change | P value |
| --- | --- | --- | --- | --- | --- | --- | --- | --- | --- | --- | --- |
| | | | | | | | | | | | |
| b-actin | C1 | 24.08655 | | Pri-miR-126 | C1 | 32.54513 | 8.458583 | 0.002843 | | | |
| b-actin | C2 | 21.72638 | | Pri-miR-126 | C2 | 32.53945 | 10.81307 | 0.000556 | | | |
| b-actin | C3 | 21.81902 | | Pri-miR-126 | C3 | 31.91502 | 10.09601 | 0.000914 | | | |
| b-actin | C4 | 22.84766 | | Pri-miR-126 | C4 | 32.32873 | 9.481077 | 0.001399 | 0.001478 | 1 | |
| b-actin | C5 | 23.56182 | | Pri-miR-126 | C5 | 32.78224 | 9.220421 | 0.001676 | 0.000877 | 0.593542 | |
| b-actin | B10 | 24.54459 | | Pri-miR-126 | B10 | 30.20946 | 5.664866 | 0.019711 | | | |
| b-actin | B19 | 23.97655 | | Pri-miR-126 | B19 | 29.87676 | 5.900211 | 0.016744 | | | |
| b-actin | B23 | 24.44548 | | Pri-miR-126 | B23 | 28.47221 | 4.026734 | 0.061353 | | | |
| b-actin | B24 | 23.73582 | | Pri-miR-126 | B24 | 29.38236 | 5.646544 | 0.019963 | | | |
| b-actin | B26 | 24.30404 | | Pri-miR-126 | B26 | 29.33323 | 5.029192 | 0.030624 | | | |
| b-actin | B29 | 23.44795 | | Pri-miR-126 | B29 | 29.34325 | 5.895297 | 0.016801 | 0.02675 | 18.10412 | 0.005854 |
| b-actin | B46 | 23.5824 | | Pri-miR-126 | B46 | 29.08521 | 5.502804 | 0.022054 | 0.015964 | 10.80434 | |
| | | | | | | | | | | | |
| | | | | | | | | | | | |
| | | | | | | | | | | | |
| b-actin | C1 | 21.8 | | EGFL7B | C1 | 32.01 | 10.21 | 0.000844 | | | |
| b-actin | C2 | 20.08 | | EGFL7B | C2 | 31.62 | 11.54 | 0.000336 | | | |
| b-actin | C3 | 20.35 | | EGFL7B | C3 | 31.14 | 10.79 | 0.000565 | | | |
| b-actin | C4 | 22.06 | | EGFL7B | C4 | 31.02 | 8.96 | 0.002008 | 0.001007 | 1 | |
| b-actin | C5 | 21.58 | | EGFL7B | C5 | 31.19 | 9.61 | 0.00128 | 0.000661 | 0.656951 | |
| b-actin | B10 | 21.98 | | EGFL7B | B10 | 30.52 | 8.54 | 0.002687 | | | |
| b-actin | B19 | 21.56 | | EGFL7B | B19 | 31.46 | 9.9 | 0.001047 | | | |
| b-actin | B23 | 22.01 | | EGFL7B | B23 | 30.08 | 8.07 | 0.003721 | | | |
| b-actin | B24 | 21.58 | | EGFL7B | B24 | 30.58 | 9 | 0.001953 | | | |
| b-actin | B26 | 22.26 | | EGFL7B | B26 | 29.89 | 7.63 | 0.005048 | | | |
| b-actin | B29 | 21.66 | | EGFL7B | B29 | 29.87 | 8.21 | 0.003377 | 0.002804 | 2.786148 | 0.021771 |
| b-actin | B46 | 22.11 | | EGFL7B | B46 | 31.23 | 9.12 | 0.001797 | 0.001357 | 1.348047 | |
| | | | | | | | | | | | |
| | | | | | | | | | | | |
| | | | | | | | | | | | |
| b-actin | C1 | 21.8 | | EGFL7C | C1 | 28.31 | 6.51 | 0.010972 | | | |
| b-actin | C2 | 20.08 | | EGFL7C | C2 | 27.9 | 7.82 | 0.004425 | | | |
| b-actin | C3 | 20.35 | | EGFL7C | C3 | 28.15 | 7.8 | 0.004487 | | | |
| b-actin | C4 | 22.06 | | EGFL7C | C4 | 28.55 | 6.49 | 0.011125 | 0.007722 | 1 | |
| b-actin | C5 | 21.58 | | EGFL7C | C5 | 28.62 | 7.04 | 0.007599 | 0.003298 | 0.427043 | |
| b-actin | B10 | 21.98 | | EGFL7C | B10 | 27.96 | 5.98 | 0.015843 | | | |
| b-actin | B19 | 21.56 | | EGFL7C | B19 | 28.3 | 6.74 | 0.009355 | | | |
| b-actin | B23 | 22.01 | | EGFL7C | B23 | 27.86 | 5.85 | 0.017337 | | | |
| b-actin | B24 | 21.58 | | EGFL7C | B24 | 27.87 | 6.29 | 0.01278 | | | |
| b-actin | B26 | 22.26 | | EGFL7C | B26 | 28 | 5.74 | 0.018711 | | | |
| b-actin | B29 | 21.66 | | EGFL7C | B29 | 27.71 | 6.05 | 0.015093 | 0.014245 | 1.844818 | 0.008339 |
| b-actin | B46 | 22.11 | | EGFL7C | B46 | 28.67 | 6.56 | 0.010598 | 0.003467 | 0.449014 | |
Fig. 2E

## Slide 3
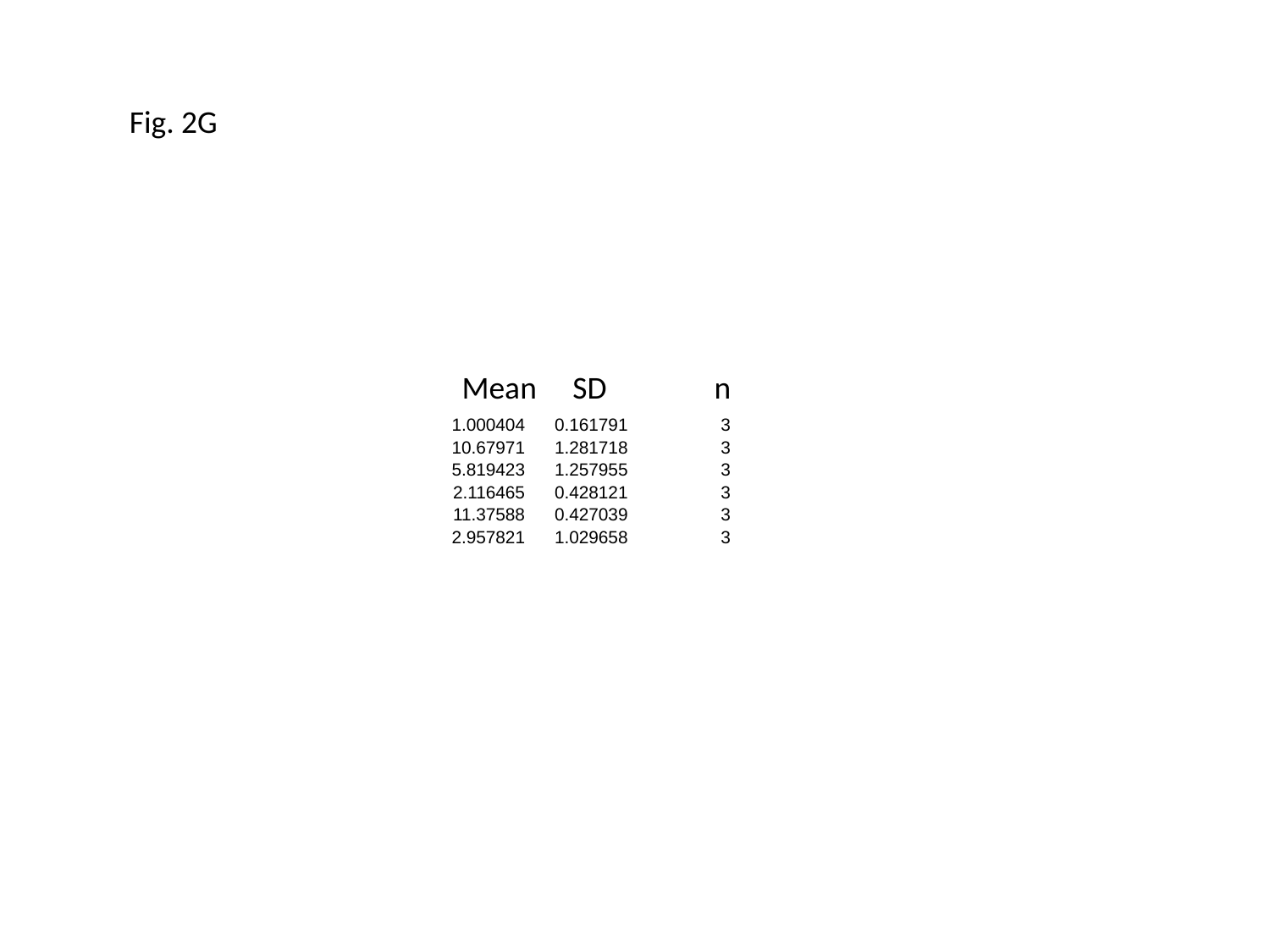

Fig. 2G
Mean SD n
| 1.000404 | 0.161791 | 3 |
| --- | --- | --- |
| 10.67971 | 1.281718 | 3 |
| 5.819423 | 1.257955 | 3 |
| 2.116465 | 0.428121 | 3 |
| 11.37588 | 0.427039 | 3 |
| 2.957821 | 1.029658 | 3 |

## Slide 4
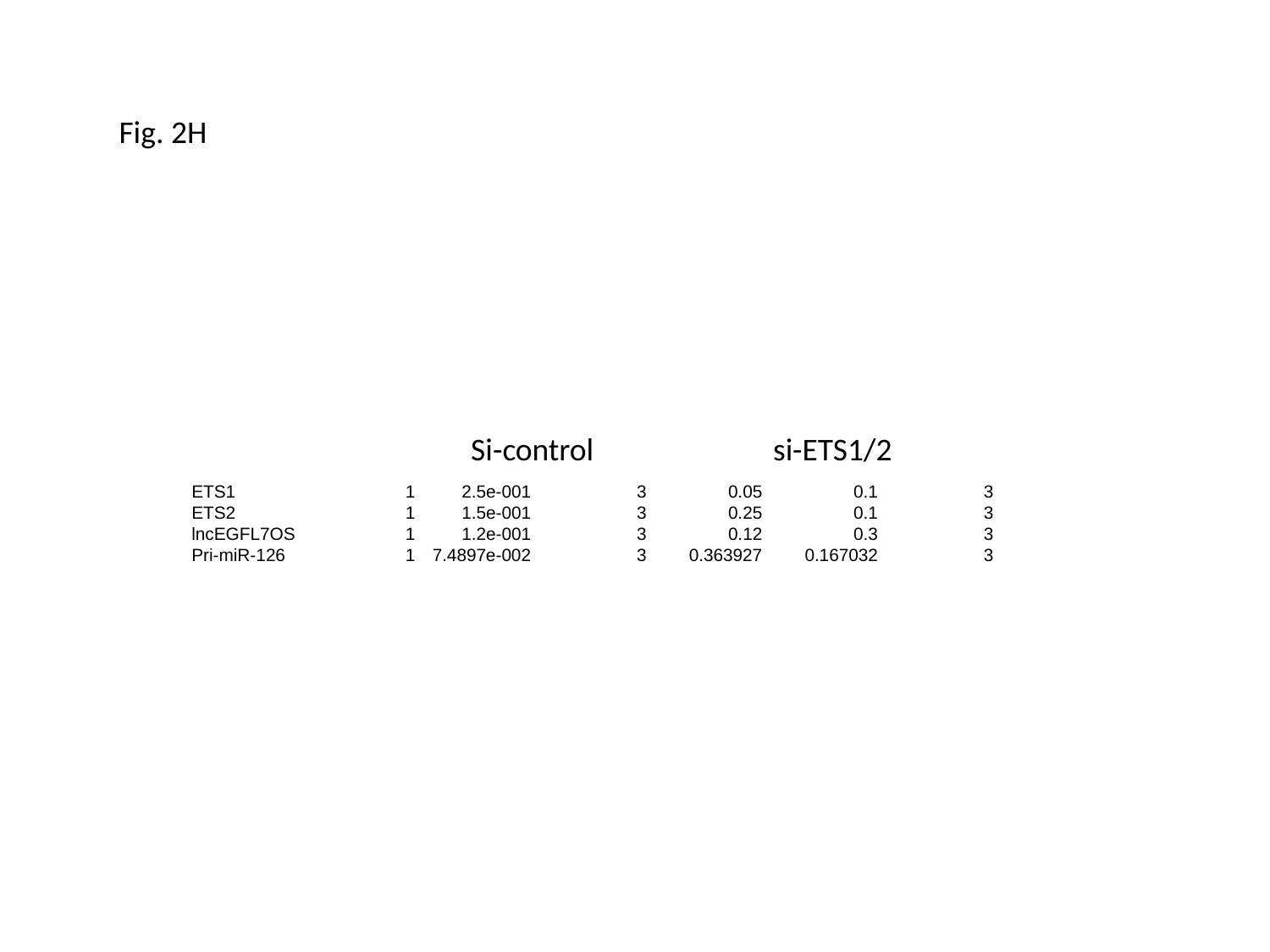

Fig. 2H
Si-control si-ETS1/2
| ETS1 | 1 | 2.5e-001 | 3 | 0.05 | 0.1 | 3 |
| --- | --- | --- | --- | --- | --- | --- |
| ETS2 | 1 | 1.5e-001 | 3 | 0.25 | 0.1 | 3 |
| lncEGFL7OS | 1 | 1.2e-001 | 3 | 0.12 | 0.3 | 3 |
| Pri-miR-126 | 1 | 7.4897e-002 | 3 | 0.363927 | 0.167032 | 3 |
