# Supplementary material for: LncEGFL7OS regulates human angiogenesis by interacting with MAX at the EGFL7/miR-126 locus
Source: eLife. 2019 Feb 11;8:e40470. doi: 10.7554/eLife.40470 (PMC6370342; doi:10.7554/eLife.40470)
Supplement: Figure 2—figure supplement 1—source data 1. [file elife-40470-fig2-figsupp1-data1.pptx]

## Slide 1
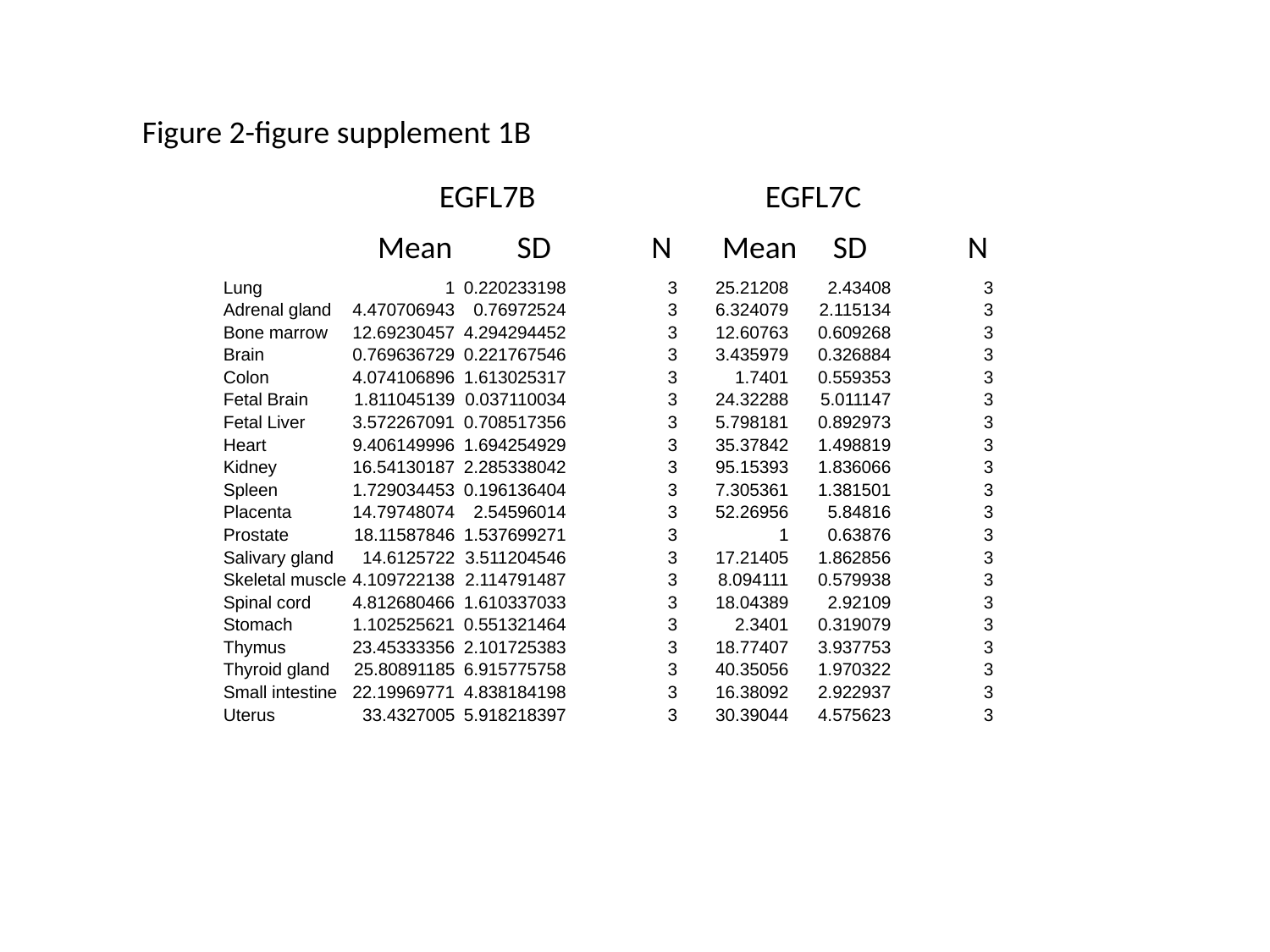

Figure 2-figure supplement 1B
EGFL7B EGFL7C
Mean SD N Mean SD N
| Lung | 1 | 0.220233198 | 3 | 25.21208 | 2.43408 | 3 |
| --- | --- | --- | --- | --- | --- | --- |
| Adrenal gland | 4.470706943 | 0.76972524 | 3 | 6.324079 | 2.115134 | 3 |
| Bone marrow | 12.69230457 | 4.294294452 | 3 | 12.60763 | 0.609268 | 3 |
| Brain | 0.769636729 | 0.221767546 | 3 | 3.435979 | 0.326884 | 3 |
| Colon | 4.074106896 | 1.613025317 | 3 | 1.7401 | 0.559353 | 3 |
| Fetal Brain | 1.811045139 | 0.037110034 | 3 | 24.32288 | 5.011147 | 3 |
| Fetal Liver | 3.572267091 | 0.708517356 | 3 | 5.798181 | 0.892973 | 3 |
| Heart | 9.406149996 | 1.694254929 | 3 | 35.37842 | 1.498819 | 3 |
| Kidney | 16.54130187 | 2.285338042 | 3 | 95.15393 | 1.836066 | 3 |
| Spleen | 1.729034453 | 0.196136404 | 3 | 7.305361 | 1.381501 | 3 |
| Placenta | 14.79748074 | 2.54596014 | 3 | 52.26956 | 5.84816 | 3 |
| Prostate | 18.11587846 | 1.537699271 | 3 | 1 | 0.63876 | 3 |
| Salivary gland | 14.6125722 | 3.511204546 | 3 | 17.21405 | 1.862856 | 3 |
| Skeletal muscle | 4.109722138 | 2.114791487 | 3 | 8.094111 | 0.579938 | 3 |
| Spinal cord | 4.812680466 | 1.610337033 | 3 | 18.04389 | 2.92109 | 3 |
| Stomach | 1.102525621 | 0.551321464 | 3 | 2.3401 | 0.319079 | 3 |
| Thymus | 23.45333356 | 2.101725383 | 3 | 18.77407 | 3.937753 | 3 |
| Thyroid gland | 25.80891185 | 6.915775758 | 3 | 40.35056 | 1.970322 | 3 |
| Small intestine | 22.19969771 | 4.838184198 | 3 | 16.38092 | 2.922937 | 3 |
| Uterus | 33.4327005 | 5.918218397 | 3 | 30.39044 | 4.575623 | 3 |

## Slide 2
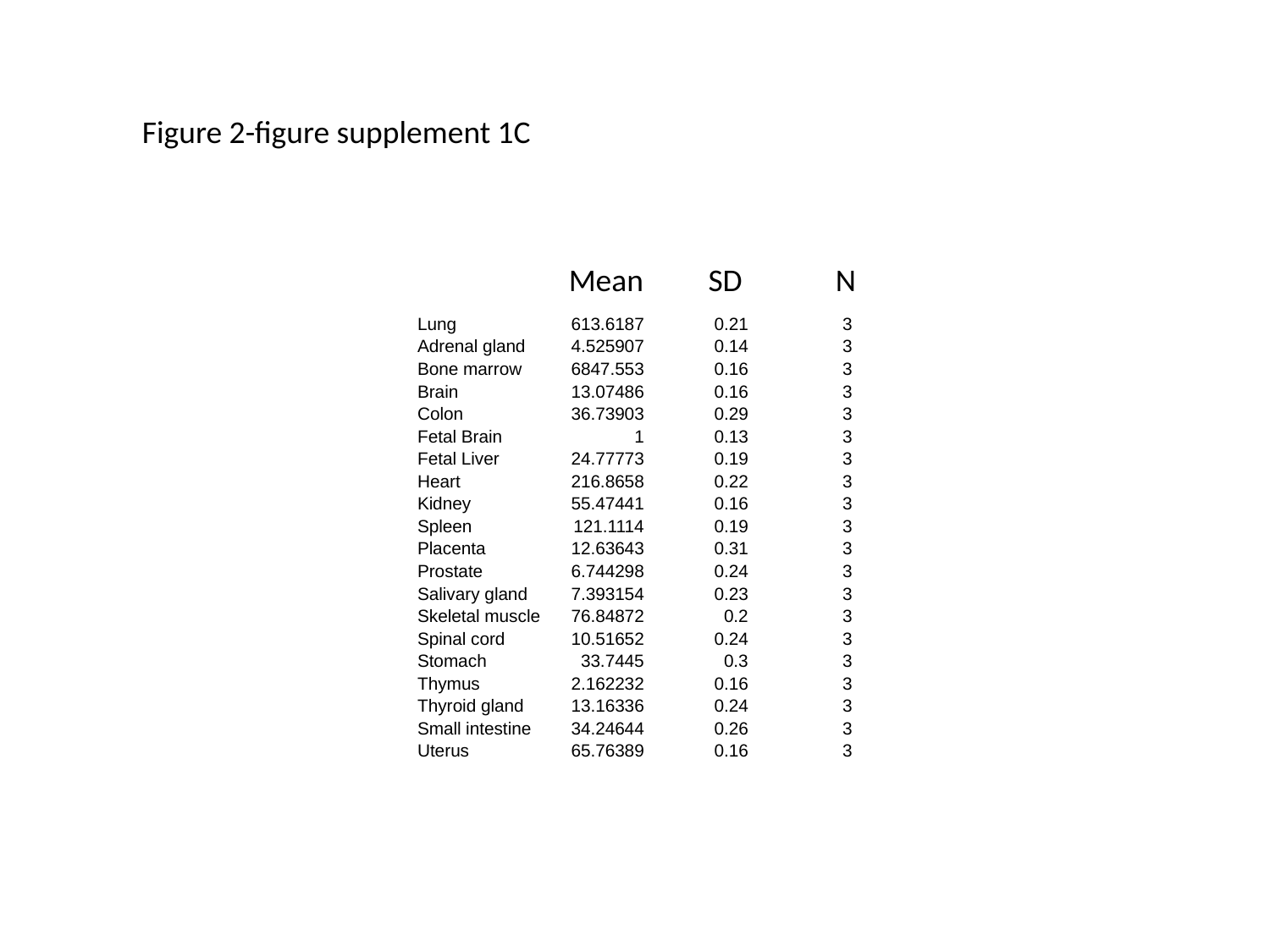

Figure 2-figure supplement 1C
Mean SD N
| Lung | 613.6187 | 0.21 | 3 |
| --- | --- | --- | --- |
| Adrenal gland | 4.525907 | 0.14 | 3 |
| Bone marrow | 6847.553 | 0.16 | 3 |
| Brain | 13.07486 | 0.16 | 3 |
| Colon | 36.73903 | 0.29 | 3 |
| Fetal Brain | 1 | 0.13 | 3 |
| Fetal Liver | 24.77773 | 0.19 | 3 |
| Heart | 216.8658 | 0.22 | 3 |
| Kidney | 55.47441 | 0.16 | 3 |
| Spleen | 121.1114 | 0.19 | 3 |
| Placenta | 12.63643 | 0.31 | 3 |
| Prostate | 6.744298 | 0.24 | 3 |
| Salivary gland | 7.393154 | 0.23 | 3 |
| Skeletal muscle | 76.84872 | 0.2 | 3 |
| Spinal cord | 10.51652 | 0.24 | 3 |
| Stomach | 33.7445 | 0.3 | 3 |
| Thymus | 2.162232 | 0.16 | 3 |
| Thyroid gland | 13.16336 | 0.24 | 3 |
| Small intestine | 34.24644 | 0.26 | 3 |
| Uterus | 65.76389 | 0.16 | 3 |

## Slide 3
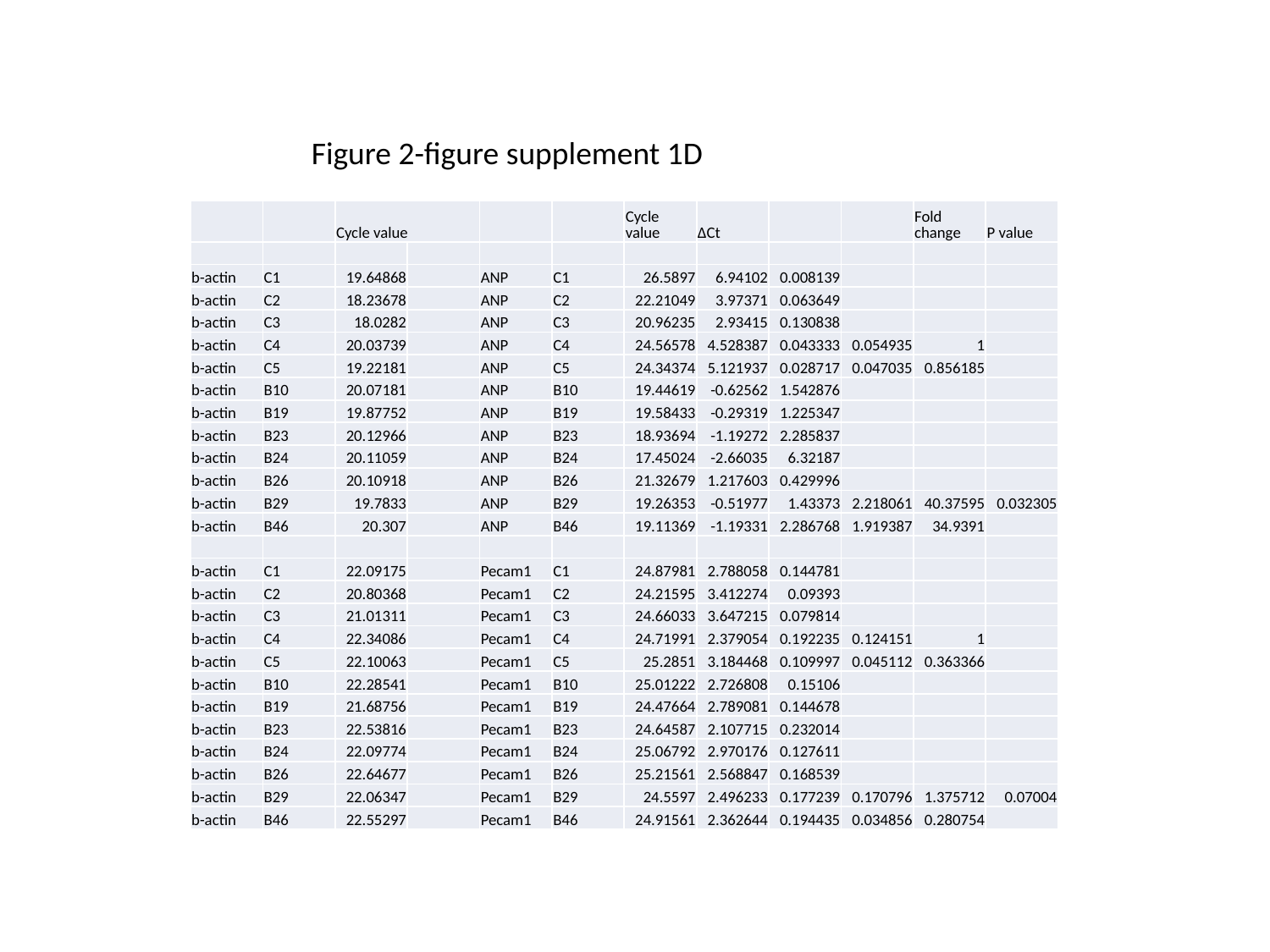

Figure 2-figure supplement 1D
| | | Cycle value | | | | Cycle value | ΔCt | | | Fold change | P value |
| --- | --- | --- | --- | --- | --- | --- | --- | --- | --- | --- | --- |
| | | | | | | | | | | | |
| b-actin | C1 | 19.64868 | | ANP | C1 | 26.5897 | 6.94102 | 0.008139 | | | |
| b-actin | C2 | 18.23678 | | ANP | C2 | 22.21049 | 3.97371 | 0.063649 | | | |
| b-actin | C3 | 18.0282 | | ANP | C3 | 20.96235 | 2.93415 | 0.130838 | | | |
| b-actin | C4 | 20.03739 | | ANP | C4 | 24.56578 | 4.528387 | 0.043333 | 0.054935 | 1 | |
| b-actin | C5 | 19.22181 | | ANP | C5 | 24.34374 | 5.121937 | 0.028717 | 0.047035 | 0.856185 | |
| b-actin | B10 | 20.07181 | | ANP | B10 | 19.44619 | -0.62562 | 1.542876 | | | |
| b-actin | B19 | 19.87752 | | ANP | B19 | 19.58433 | -0.29319 | 1.225347 | | | |
| b-actin | B23 | 20.12966 | | ANP | B23 | 18.93694 | -1.19272 | 2.285837 | | | |
| b-actin | B24 | 20.11059 | | ANP | B24 | 17.45024 | -2.66035 | 6.32187 | | | |
| b-actin | B26 | 20.10918 | | ANP | B26 | 21.32679 | 1.217603 | 0.429996 | | | |
| b-actin | B29 | 19.7833 | | ANP | B29 | 19.26353 | -0.51977 | 1.43373 | 2.218061 | 40.37595 | 0.032305 |
| b-actin | B46 | 20.307 | | ANP | B46 | 19.11369 | -1.19331 | 2.286768 | 1.919387 | 34.9391 | |
| | | | | | | | | | | | |
| b-actin | C1 | 22.09175 | | Pecam1 | C1 | 24.87981 | 2.788058 | 0.144781 | | | |
| b-actin | C2 | 20.80368 | | Pecam1 | C2 | 24.21595 | 3.412274 | 0.09393 | | | |
| b-actin | C3 | 21.01311 | | Pecam1 | C3 | 24.66033 | 3.647215 | 0.079814 | | | |
| b-actin | C4 | 22.34086 | | Pecam1 | C4 | 24.71991 | 2.379054 | 0.192235 | 0.124151 | 1 | |
| b-actin | C5 | 22.10063 | | Pecam1 | C5 | 25.2851 | 3.184468 | 0.109997 | 0.045112 | 0.363366 | |
| b-actin | B10 | 22.28541 | | Pecam1 | B10 | 25.01222 | 2.726808 | 0.15106 | | | |
| b-actin | B19 | 21.68756 | | Pecam1 | B19 | 24.47664 | 2.789081 | 0.144678 | | | |
| b-actin | B23 | 22.53816 | | Pecam1 | B23 | 24.64587 | 2.107715 | 0.232014 | | | |
| b-actin | B24 | 22.09774 | | Pecam1 | B24 | 25.06792 | 2.970176 | 0.127611 | | | |
| b-actin | B26 | 22.64677 | | Pecam1 | B26 | 25.21561 | 2.568847 | 0.168539 | | | |
| b-actin | B29 | 22.06347 | | Pecam1 | B29 | 24.5597 | 2.496233 | 0.177239 | 0.170796 | 1.375712 | 0.07004 |
| b-actin | B46 | 22.55297 | | Pecam1 | B46 | 24.91561 | 2.362644 | 0.194435 | 0.034856 | 0.280754 | |
