# Supplementary material for: LncEGFL7OS regulates human angiogenesis by interacting with MAX at the EGFL7/miR-126 locus
Source: eLife. 2019 Feb 11;8:e40470. doi: 10.7554/eLife.40470 (PMC6370342; doi:10.7554/eLife.40470)
Supplement: Figure 3—source data 1. [file elife-40470-fig3-data1.pptx]

## Slide 1
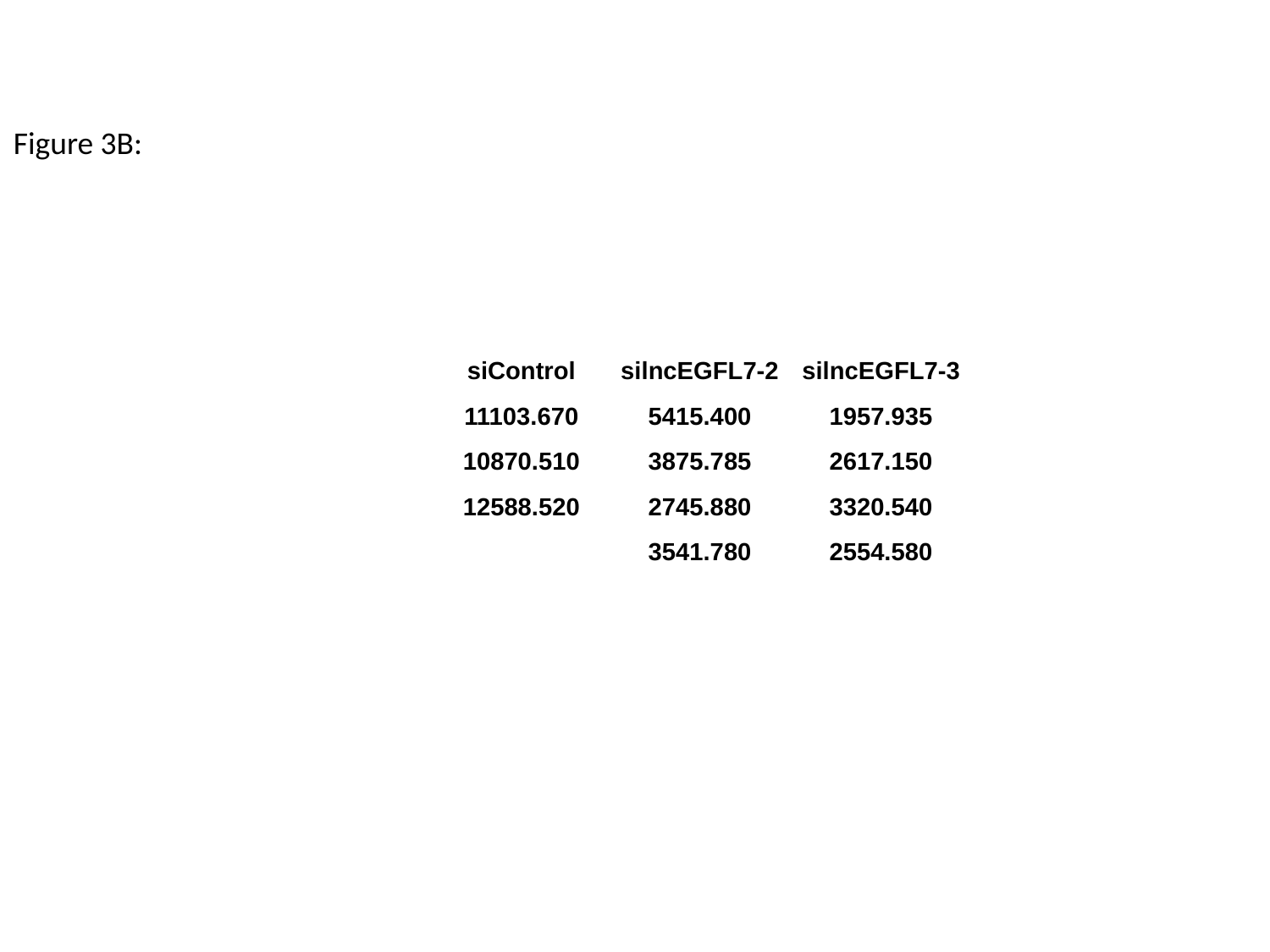

Figure 3B:
| siControl | silncEGFL7-2 | silncEGFL7-3 |
| --- | --- | --- |
| 11103.670 | 5415.400 | 1957.935 |
| 10870.510 | 3875.785 | 2617.150 |
| 12588.520 | 2745.880 | 3320.540 |
| | 3541.780 | 2554.580 |

## Slide 2
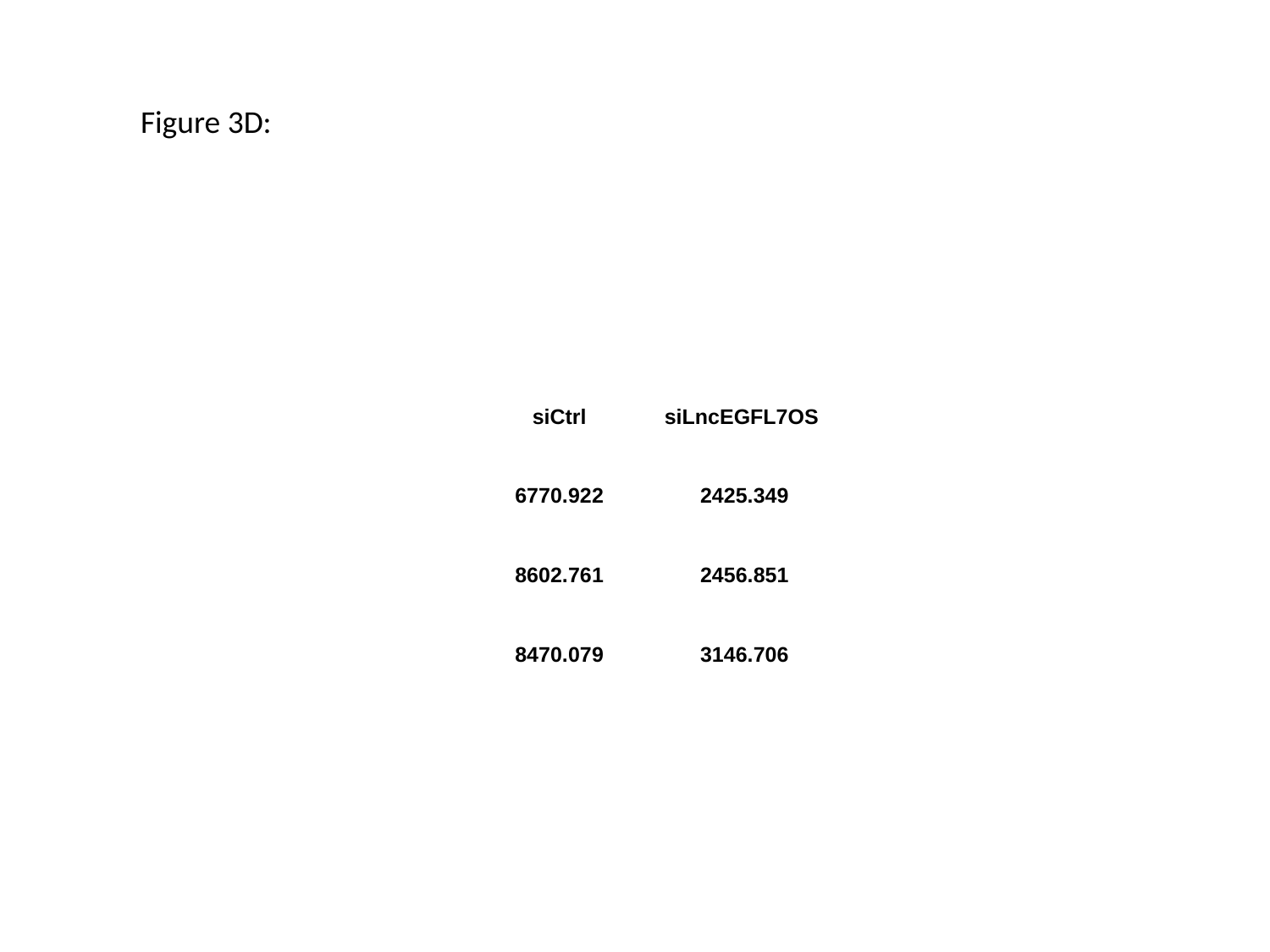

Figure 3D:
| siCtrl | siLncEGFL7OS |
| --- | --- |
| 6770.922 | 2425.349 |
| 8602.761 | 2456.851 |
| 8470.079 | 3146.706 |

## Slide 3
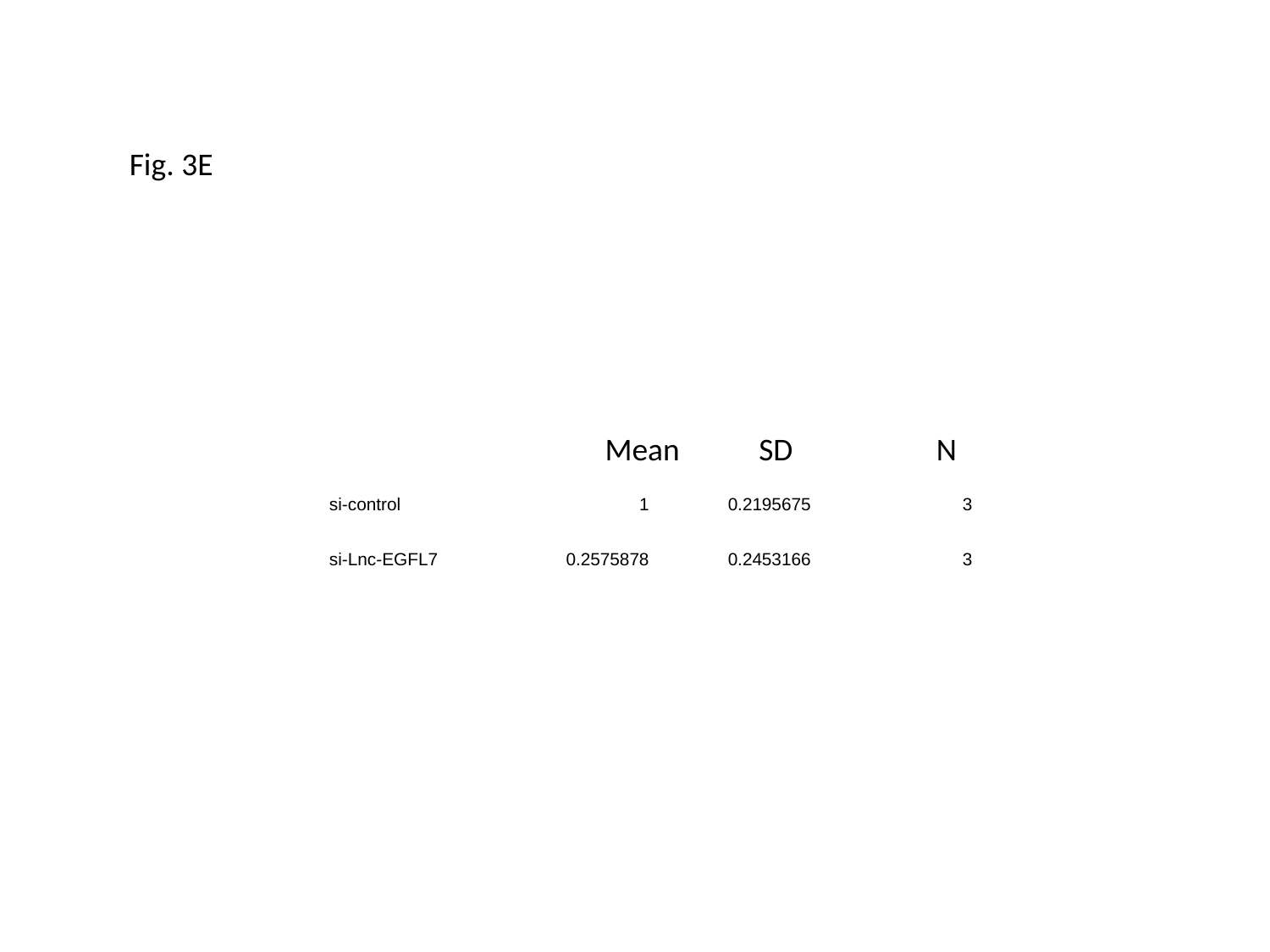

Fig. 3E
Mean SD N
| si-control | 1 | 0.2195675 | 3 |
| --- | --- | --- | --- |
| si-Lnc-EGFL7 | 0.2575878 | 0.2453166 | 3 |

## Slide 4
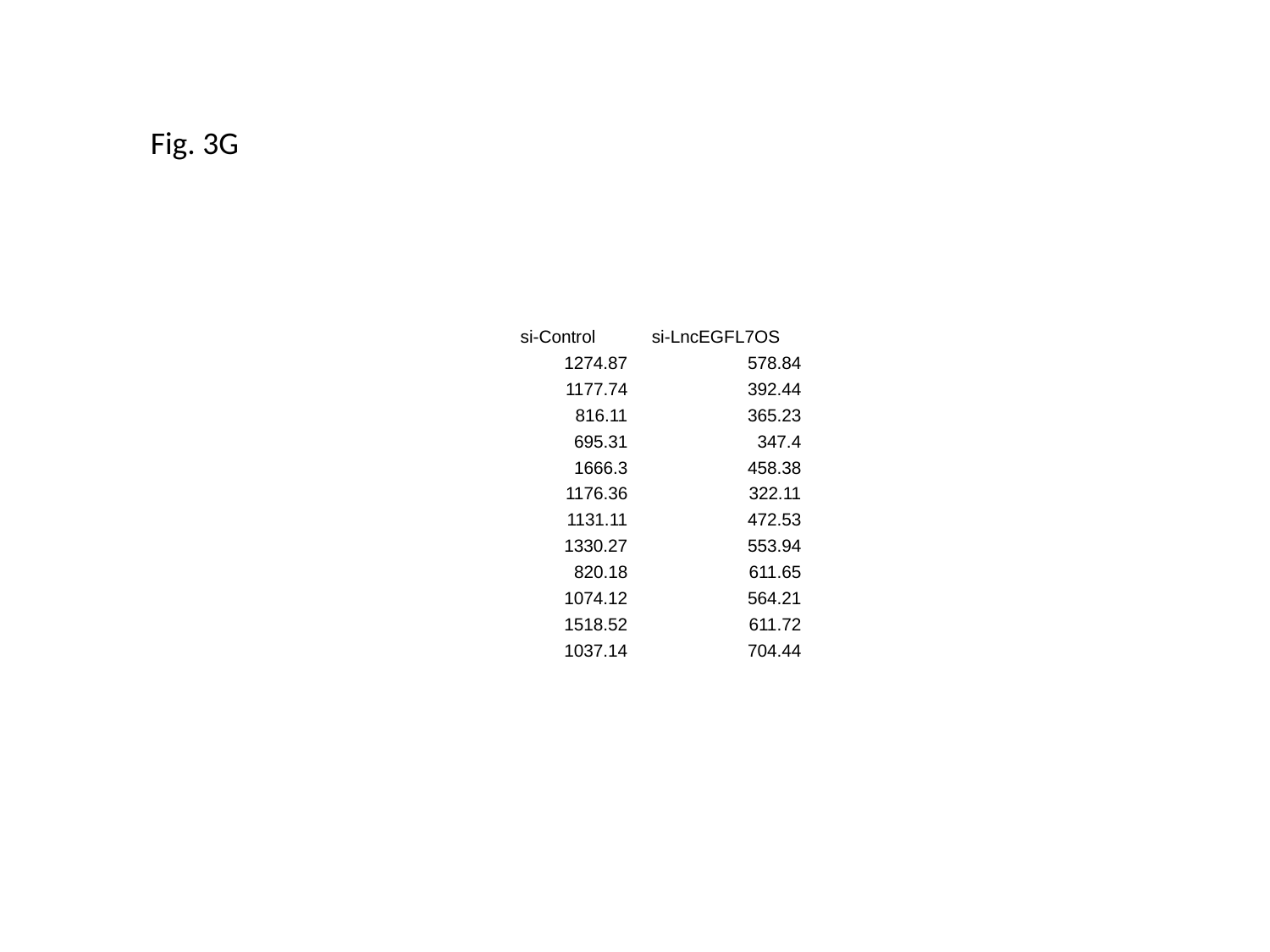

Fig. 3G
| si-Control | si-LncEGFL7OS |
| --- | --- |
| 1274.87 | 578.84 |
| 1177.74 | 392.44 |
| 816.11 | 365.23 |
| 695.31 | 347.4 |
| 1666.3 | 458.38 |
| 1176.36 | 322.11 |
| 1131.11 | 472.53 |
| 1330.27 | 553.94 |
| 820.18 | 611.65 |
| 1074.12 | 564.21 |
| 1518.52 | 611.72 |
| 1037.14 | 704.44 |
