# Supplementary figures and images for: LncEGFL7OS regulates human angiogenesis by interacting with MAX at the EGFL7/miR-126 locus
Source: eLife. 2019 Feb 11;8:e40470. doi: 10.7554/eLife.40470 (PMC6370342; doi:10.7554/eLife.40470)

## Slide 1
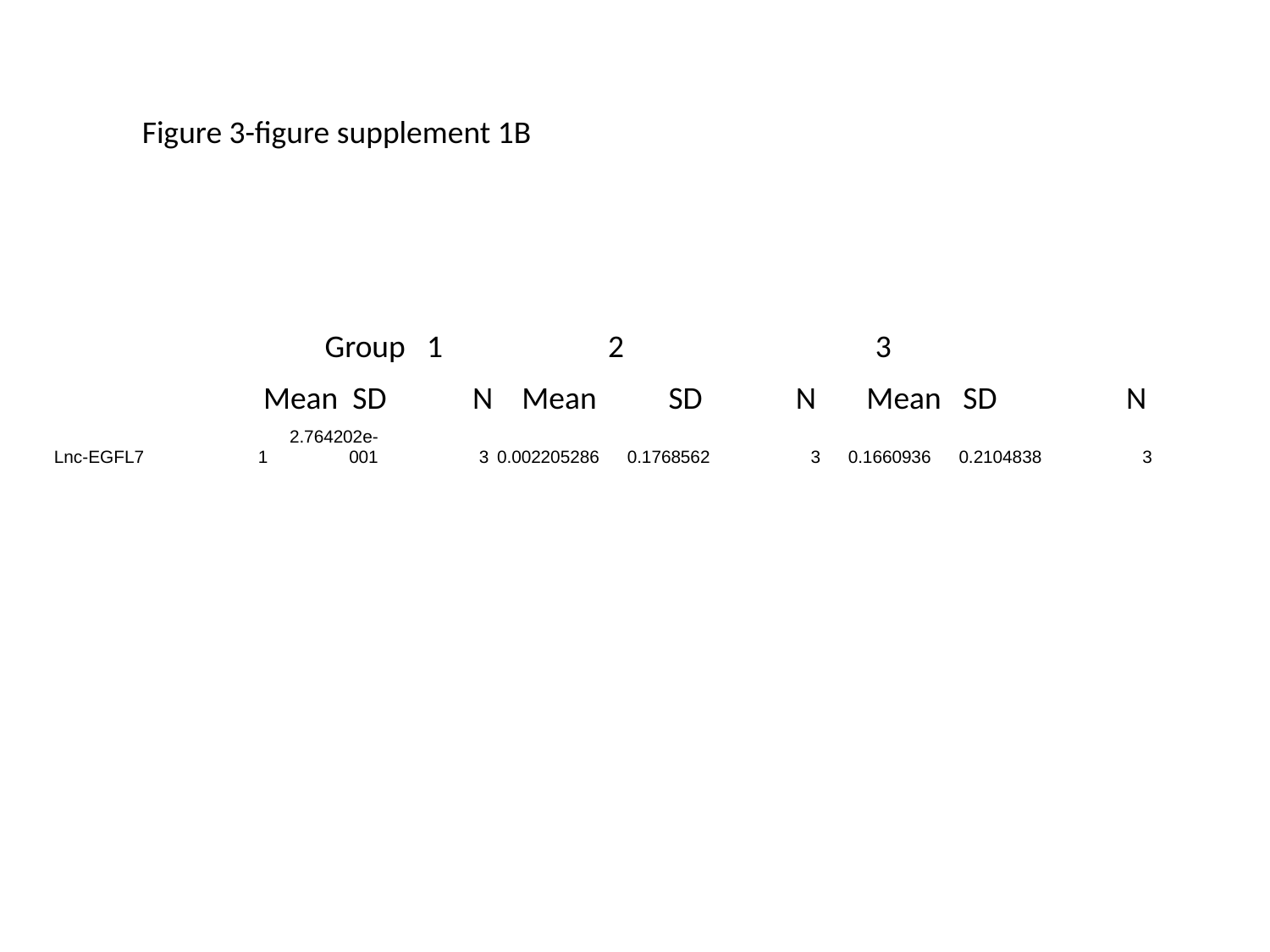

Figure 3-figure supplement 1B
Group 1 2 3
Mean SD N Mean SD N Mean SD N
| Lnc-EGFL7 | 1 | 2.764202e-001 | 3 | 0.002205286 | 0.1768562 | 3 | 0.1660936 | 0.2104838 | 3 |
| --- | --- | --- | --- | --- | --- | --- | --- | --- | --- |
| | | | | | | | | | |

Supplement: Figure 3—figure supplement 1—source data 1. [file elife-40470-fig3-figsupp1-data1.pptx]

## Slide 1
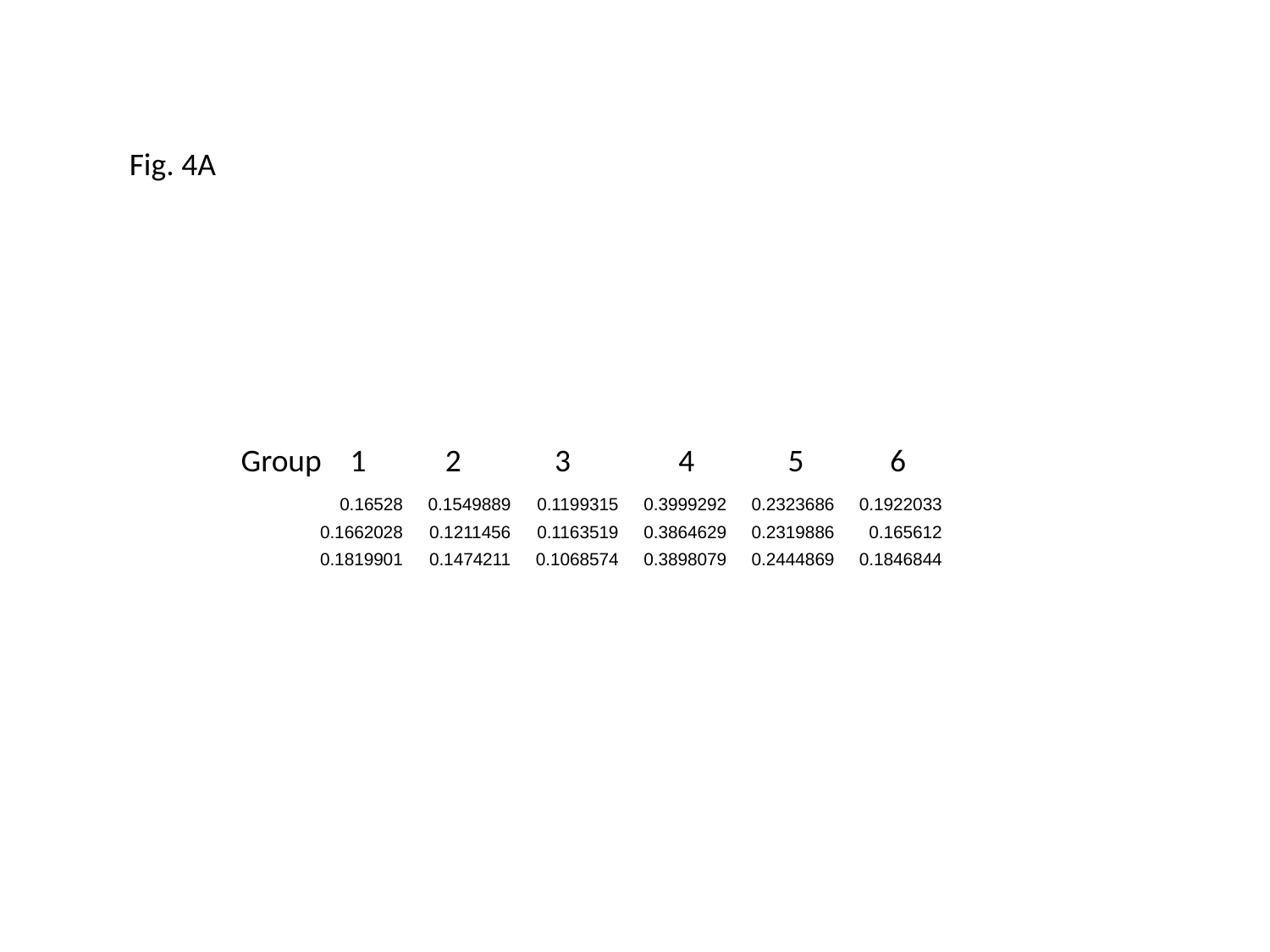

Fig. 4A
Group 1 2 3 4 5 6
| 0.16528 | 0.1549889 | 0.1199315 | 0.3999292 | 0.2323686 | 0.1922033 |
| --- | --- | --- | --- | --- | --- |
| 0.1662028 | 0.1211456 | 0.1163519 | 0.3864629 | 0.2319886 | 0.165612 |
| 0.1819901 | 0.1474211 | 0.1068574 | 0.3898079 | 0.2444869 | 0.1846844 |

## Slide 2
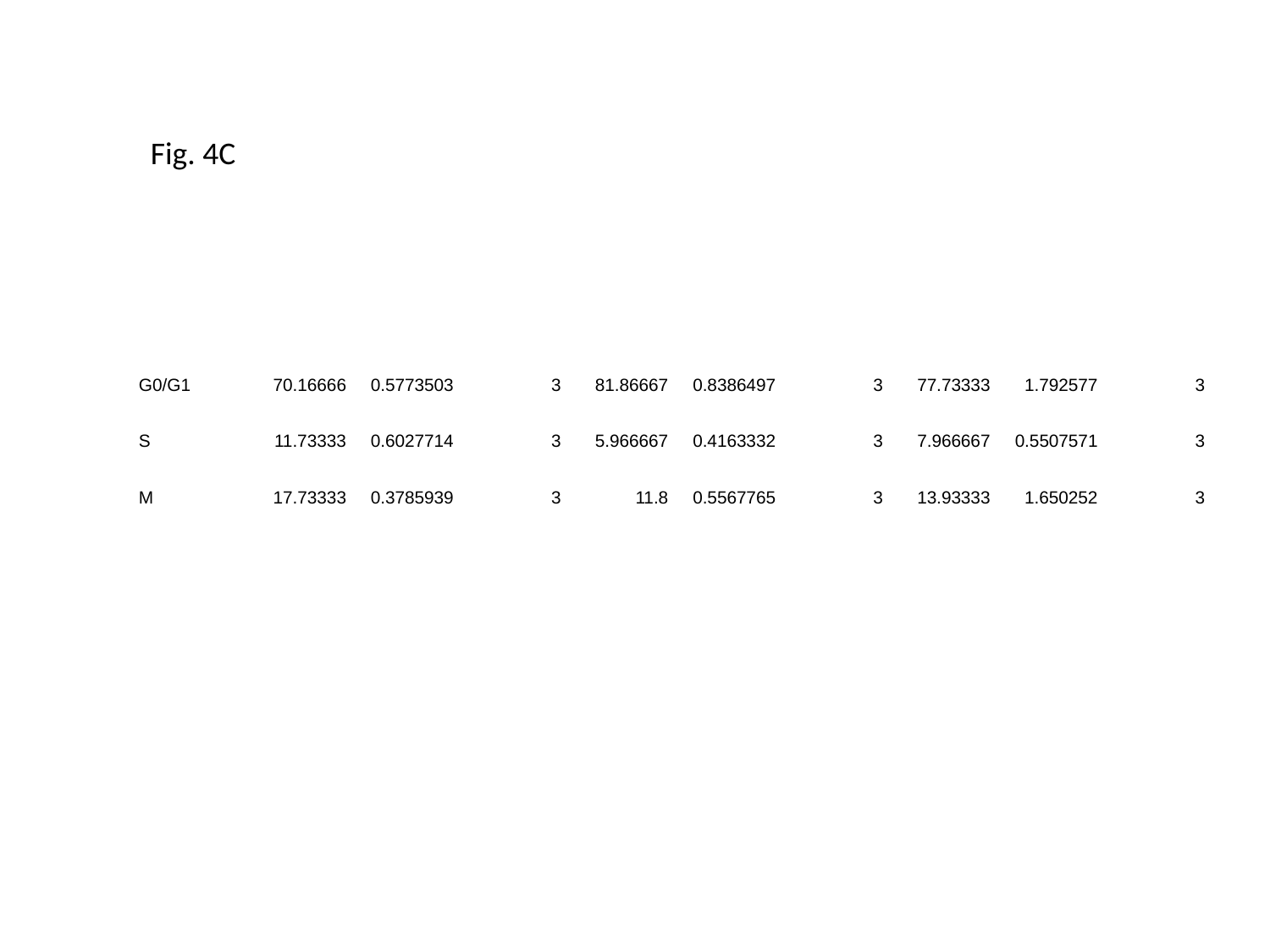

Fig. 4C
| G0/G1 | 70.16666 | 0.5773503 | 3 | 81.86667 | 0.8386497 | 3 | 77.73333 | 1.792577 | 3 |
| --- | --- | --- | --- | --- | --- | --- | --- | --- | --- |
| S | 11.73333 | 0.6027714 | 3 | 5.966667 | 0.4163332 | 3 | 7.966667 | 0.5507571 | 3 |
| M | 17.73333 | 0.3785939 | 3 | 11.8 | 0.5567765 | 3 | 13.93333 | 1.650252 | 3 |

## Slide 3
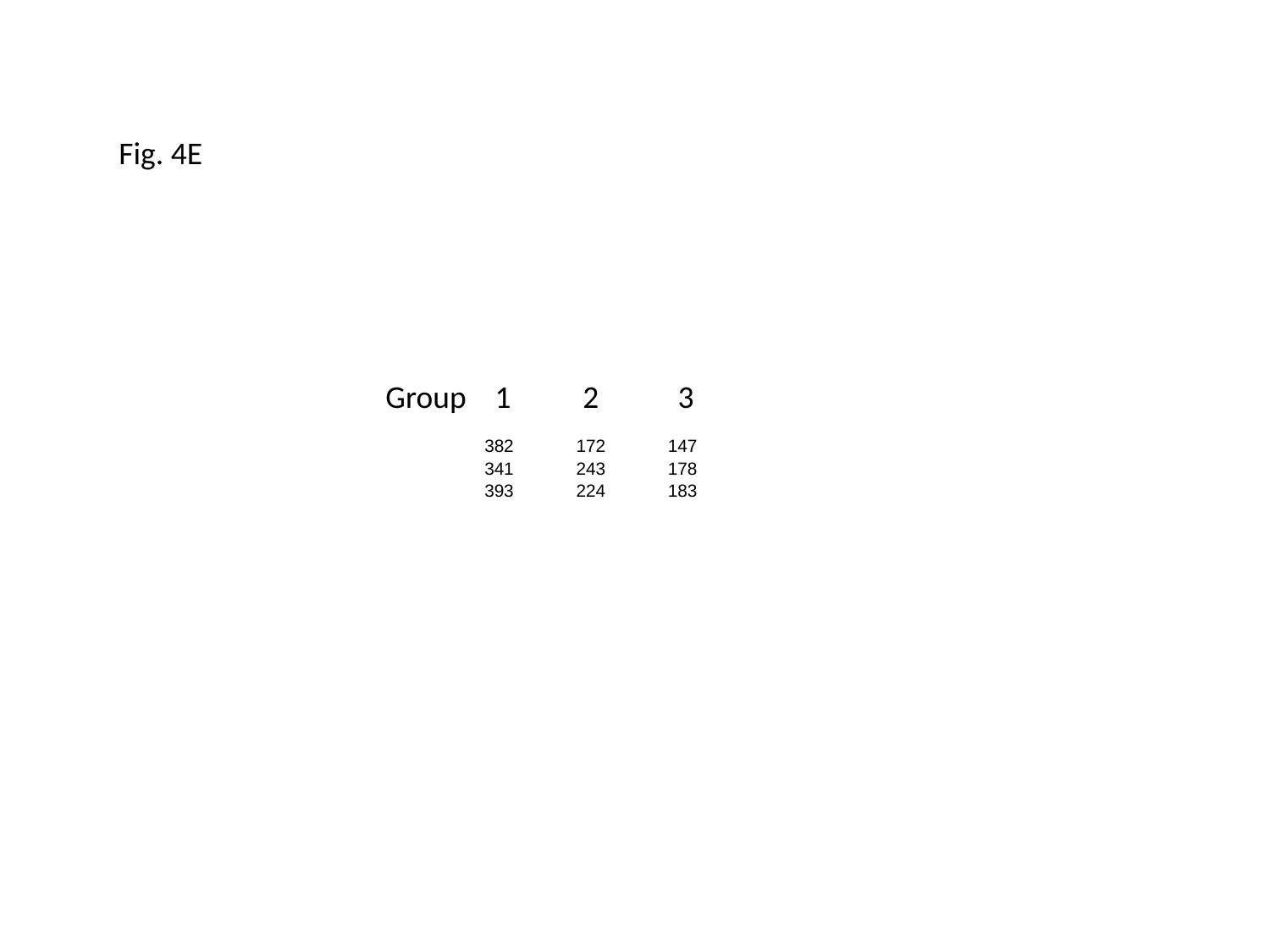

Fig. 4E
Group 1 2 3
| 382 | 172 | 147 |
| --- | --- | --- |
| 341 | 243 | 178 |
| 393 | 224 | 183 |

Supplement: Figure 4—source data 1. [file elife-40470-fig4-data1.pptx]

## Slide 1
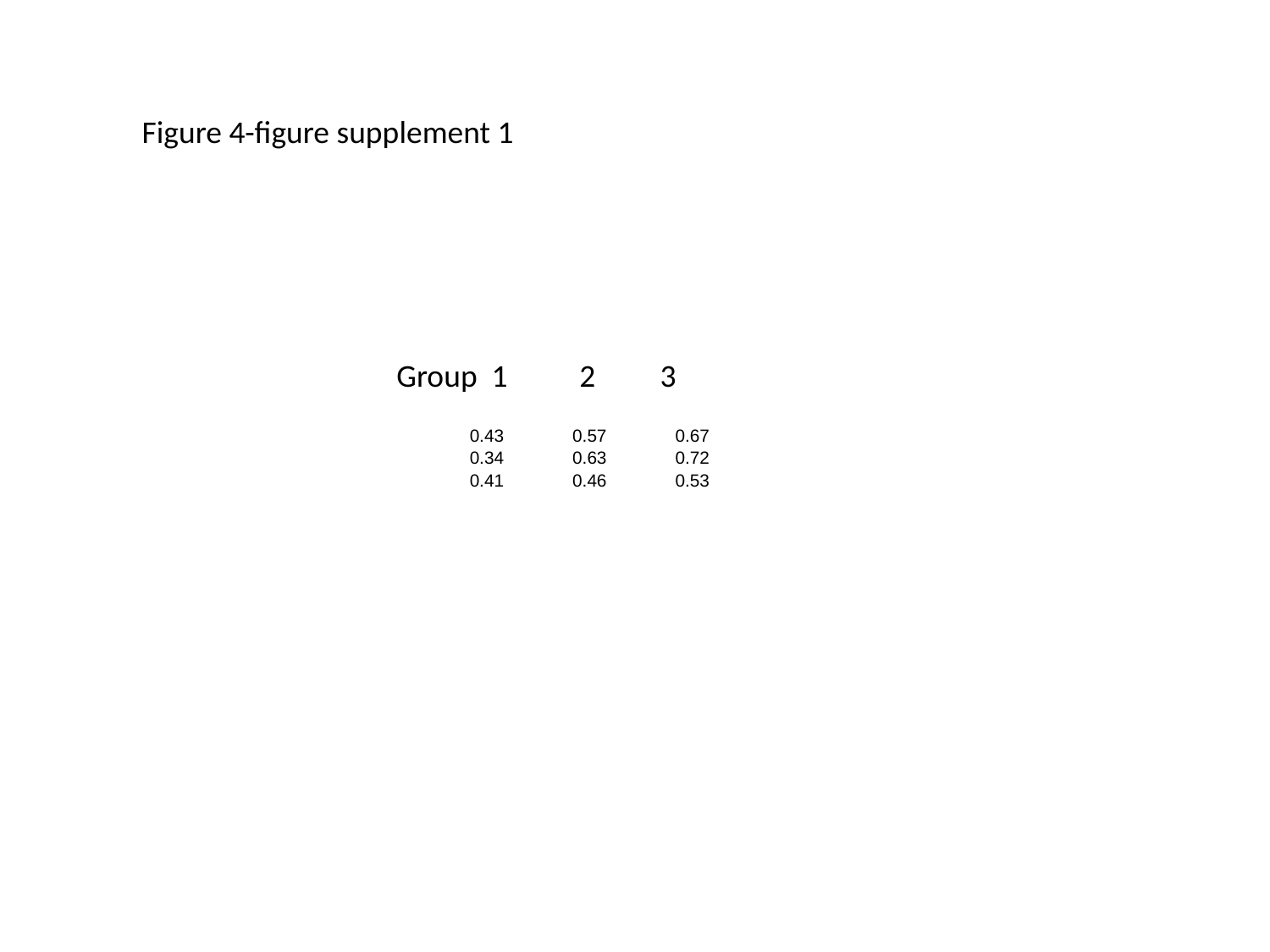

Figure 4-figure supplement 1
Group 1 2 3
| 0.43 | 0.57 | 0.67 |
| --- | --- | --- |
| 0.34 | 0.63 | 0.72 |
| 0.41 | 0.46 | 0.53 |

Supplement: Figure 4—figure supplement 1—source data 1. [file elife-40470-fig4-figsupp1-data1.pptx]

## Slide 1
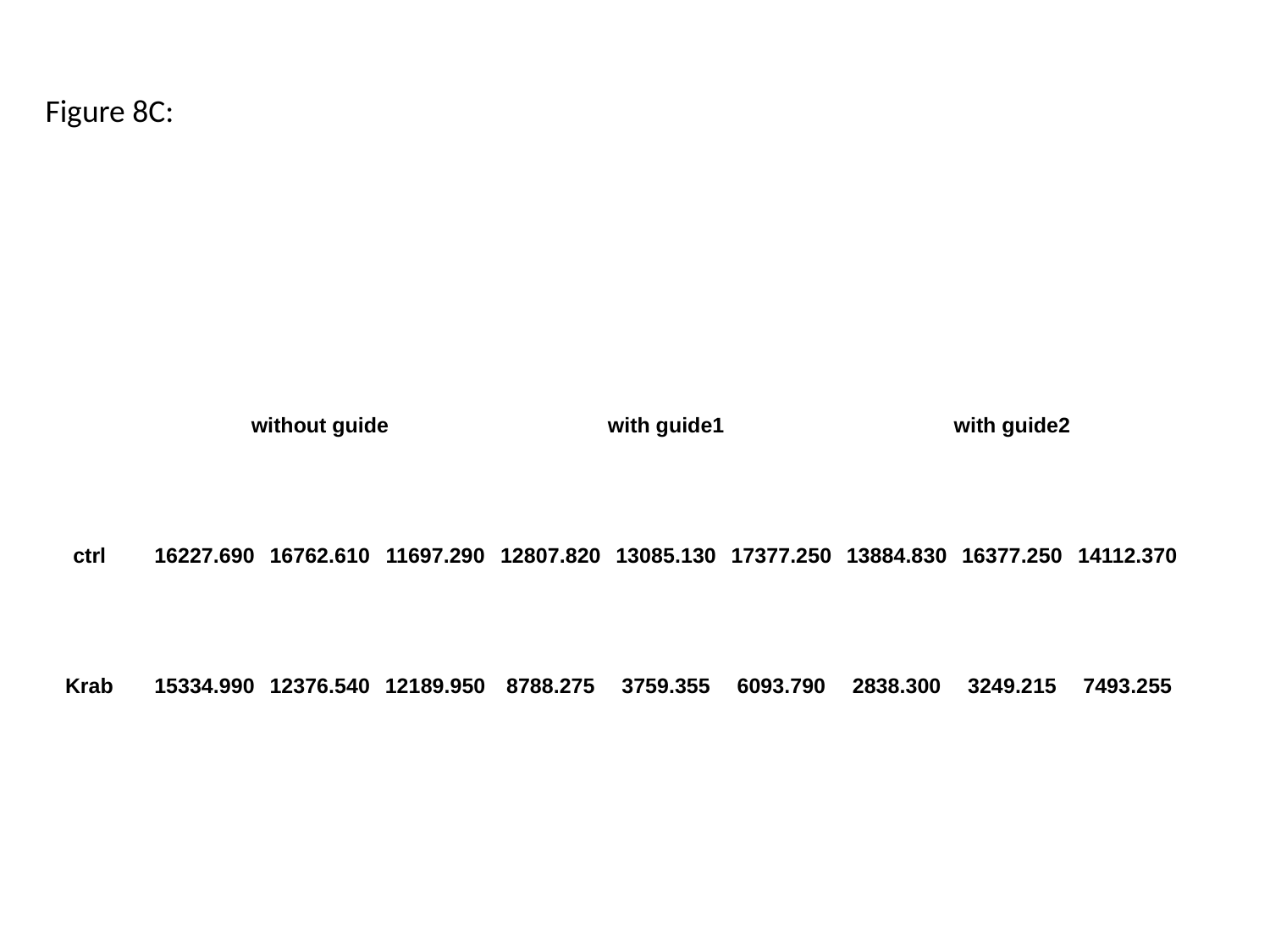

Figure 8C:
| | without guide | | | with guide1 | | | with guide2 | | |
| --- | --- | --- | --- | --- | --- | --- | --- | --- | --- |
| ctrl | 16227.690 | 16762.610 | 11697.290 | 12807.820 | 13085.130 | 17377.250 | 13884.830 | 16377.250 | 14112.370 |
| Krab | 15334.990 | 12376.540 | 12189.950 | 8788.275 | 3759.355 | 6093.790 | 2838.300 | 3249.215 | 7493.255 |

## Slide 2
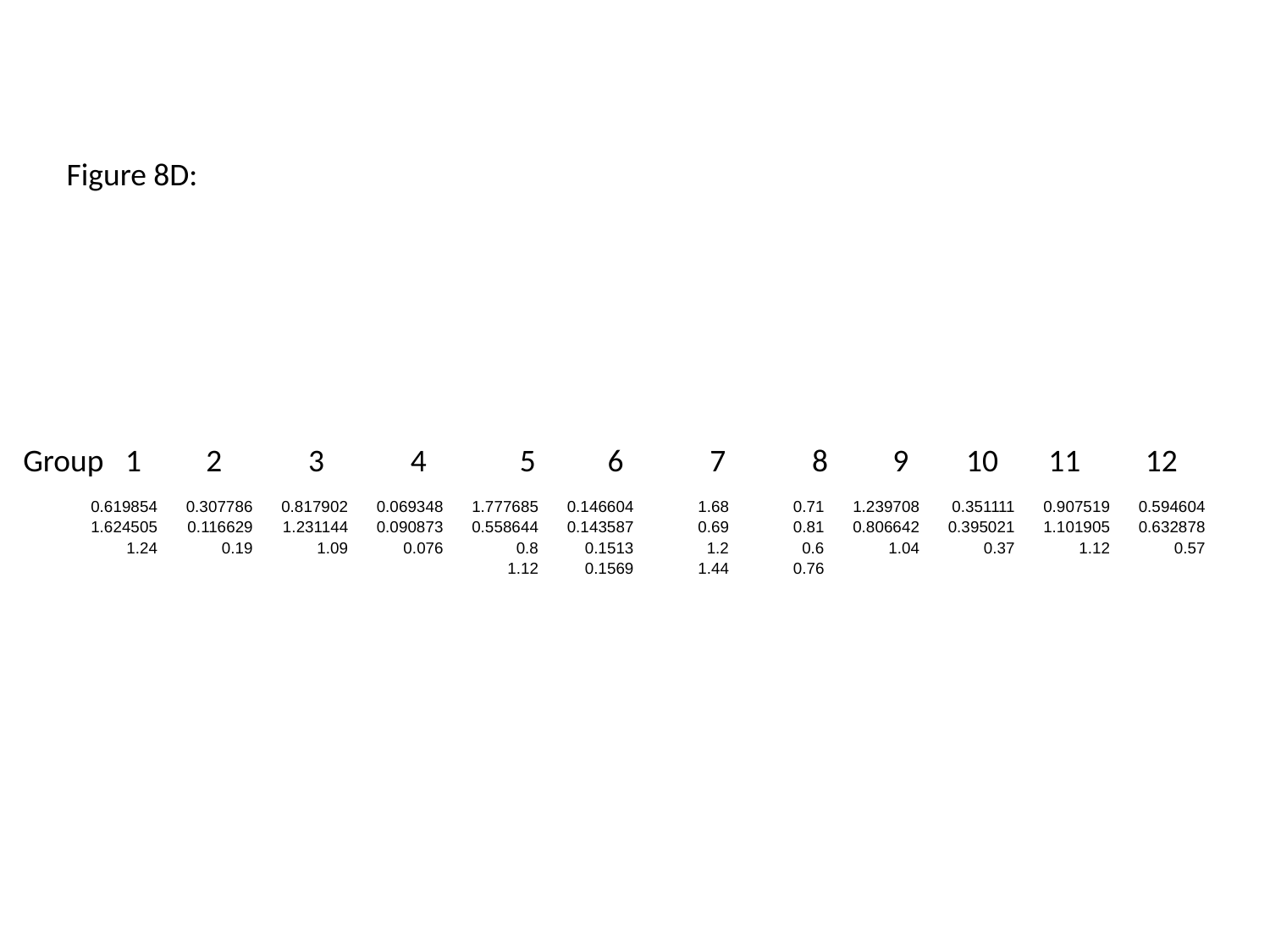

Figure 8D:
Group 1 2 3 4 5 6 7 8 9 10 11 12
| 0.619854 | 0.307786 | 0.817902 | 0.069348 | 1.777685 | 0.146604 | 1.68 | 0.71 | 1.239708 | 0.351111 | 0.907519 | 0.594604 |
| --- | --- | --- | --- | --- | --- | --- | --- | --- | --- | --- | --- |
| 1.624505 | 0.116629 | 1.231144 | 0.090873 | 0.558644 | 0.143587 | 0.69 | 0.81 | 0.806642 | 0.395021 | 1.101905 | 0.632878 |
| 1.24 | 0.19 | 1.09 | 0.076 | 0.8 | 0.1513 | 1.2 | 0.6 | 1.04 | 0.37 | 1.12 | 0.57 |
| | | | | 1.12 | 0.1569 | 1.44 | 0.76 | | | | |

Supplement: Figure 8—source data 1. [file elife-40470-fig8-data1.pptx]

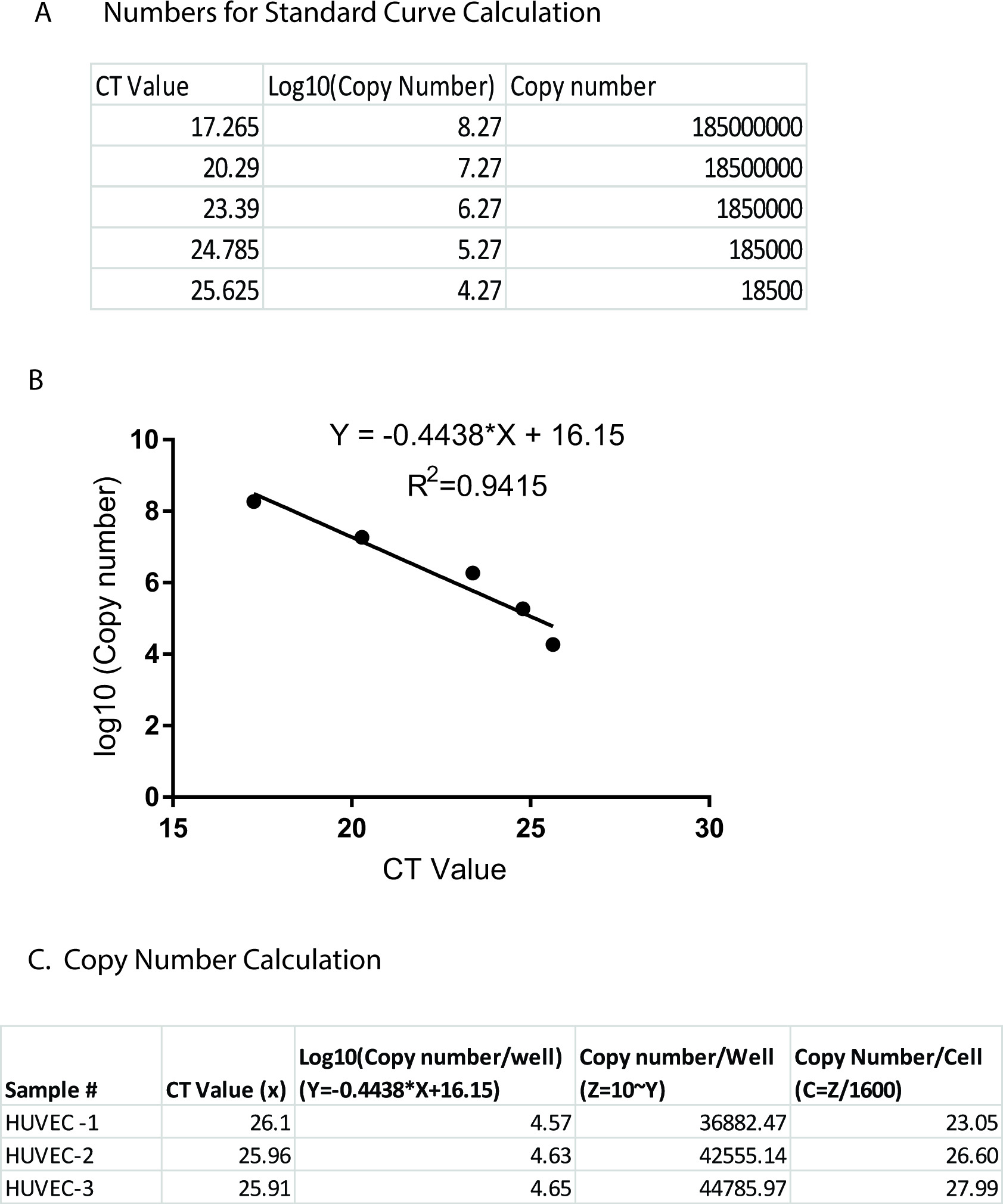

Supplement: Supplementary file 4. — The RNA was harvested at 1.85*1011 copies per µl. After reverse transcription, 1 µl the cDNA was diluted at 103, 104, 105, 106 and 107 times, respectively, as templates to carry out Real-time PCR. The copy numbers were calculated based on the dilution folds. (B) The CT values and the log10 (Copy number) were used to establish the standard curve and formulation for copy number calculation. The Log10 (copy number) and CT value relation can be modeled as: Y = −0.4438*X + 16.15. R square is 0.9415. (C) The formulation in (B) was used to calculate the copy number per well of the HUVEC cell samples. Based on the calculation that each well has ~1600 cells, the copy number per cell was calculated. [file elife-40470-supp4.jpg]
