# Supplementary material for: LncEGFL7OS regulates human angiogenesis by interacting with MAX at the EGFL7/miR-126 locus
Source: eLife. 2019 Feb 11;8:e40470. doi: 10.7554/eLife.40470 (PMC6370342; doi:10.7554/eLife.40470)
Supplement: Figure 4—figure supplement 2—source data 1. [file elife-40470-fig4-figsupp2-data1.pptx]

## Slide 1
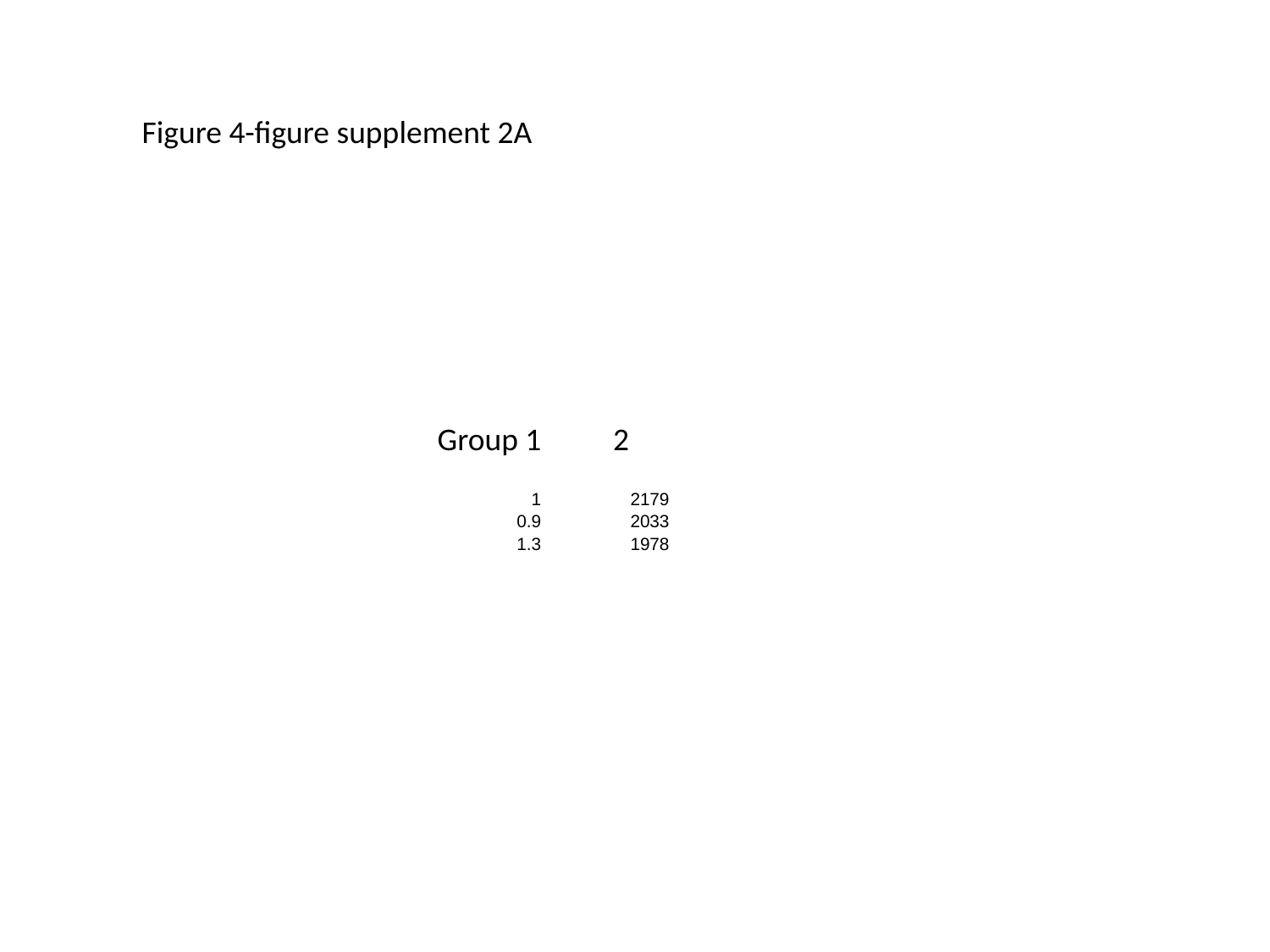

Figure 4-figure supplement 2A
Group 1 2
| 1 | 2179 |
| --- | --- |
| 0.9 | 2033 |
| 1.3 | 1978 |

## Slide 2
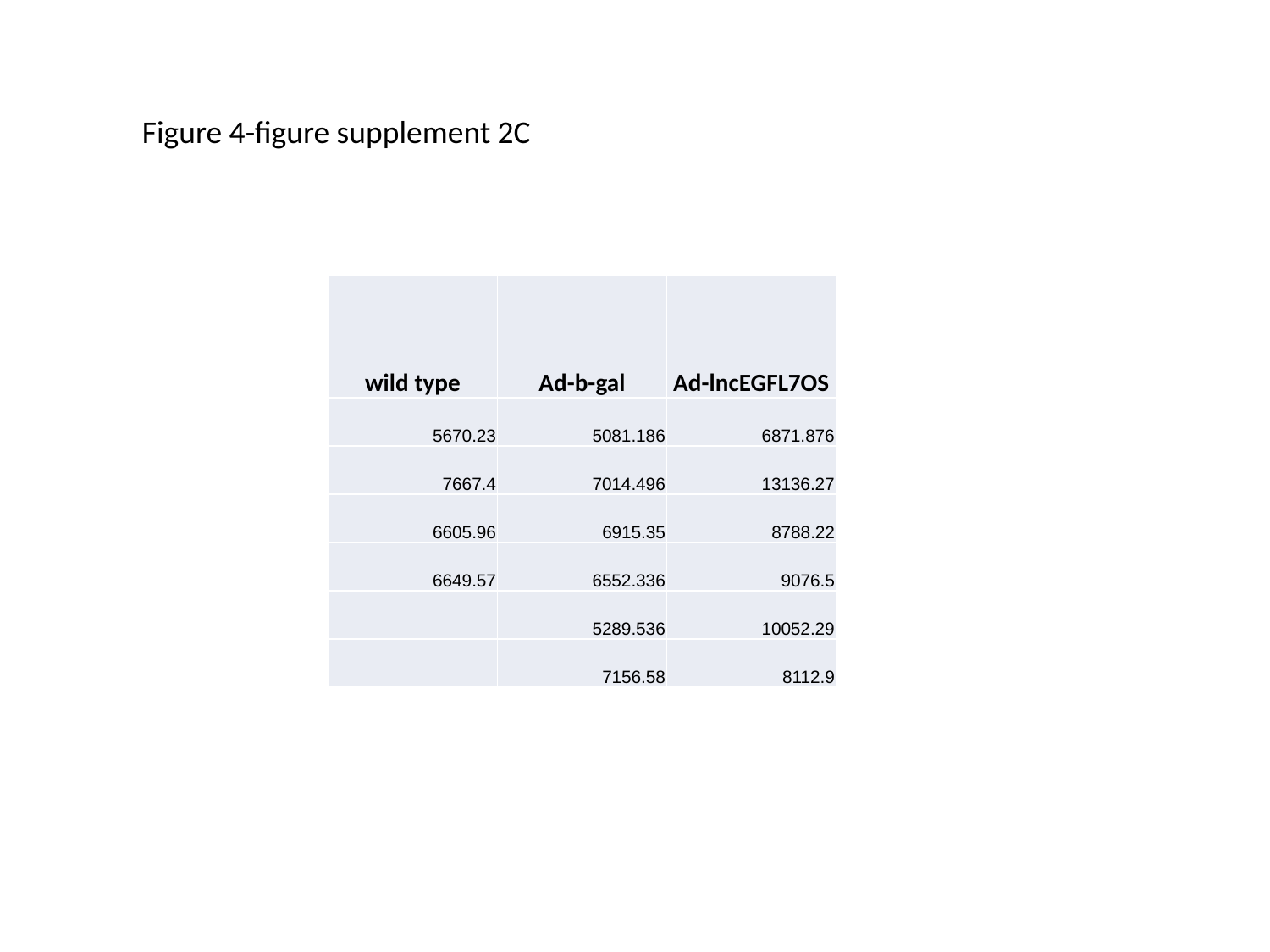

Figure 4-figure supplement 2C
| wild type | Ad-b-gal | Ad-lncEGFL7OS |
| --- | --- | --- |
| 5670.23 | 5081.186 | 6871.876 |
| 7667.4 | 7014.496 | 13136.27 |
| 6605.96 | 6915.35 | 8788.22 |
| 6649.57 | 6552.336 | 9076.5 |
| | 5289.536 | 10052.29 |
| | 7156.58 | 8112.9 |
