# Supplementary material for: LncEGFL7OS regulates human angiogenesis by interacting with MAX at the EGFL7/miR-126 locus
Source: eLife. 2019 Feb 11;8:e40470. doi: 10.7554/eLife.40470 (PMC6370342; doi:10.7554/eLife.40470)
Supplement: Figure 5—source data 1. [file elife-40470-fig5-data1.pptx]

## Slide 1
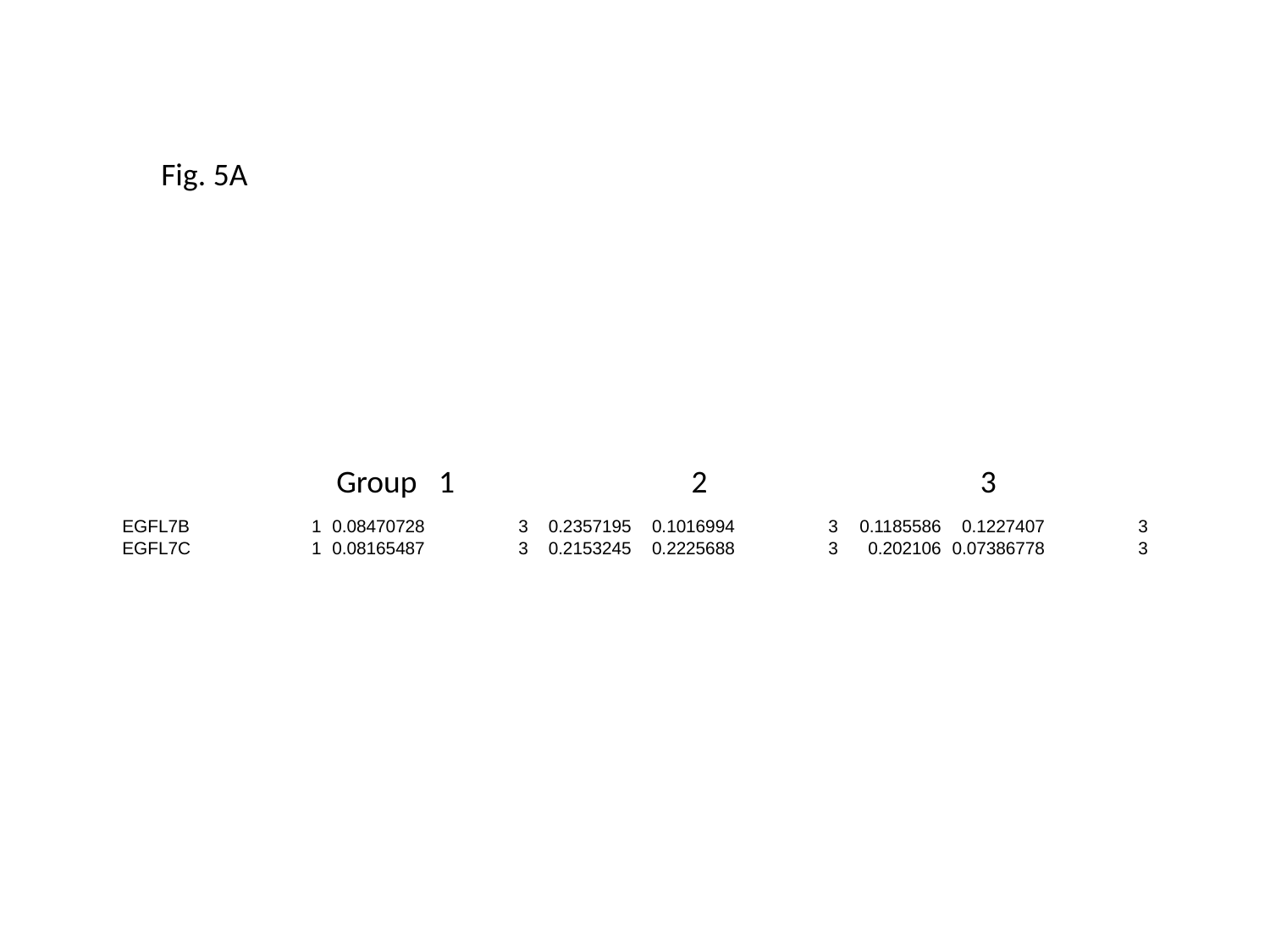

Fig. 5A
Group 1 2 3
| EGFL7B | 1 | 0.08470728 | 3 | 0.2357195 | 0.1016994 | 3 | 0.1185586 | 0.1227407 | 3 |
| --- | --- | --- | --- | --- | --- | --- | --- | --- | --- |
| EGFL7C | 1 | 0.08165487 | 3 | 0.2153245 | 0.2225688 | 3 | 0.202106 | 0.07386778 | 3 |

## Slide 2
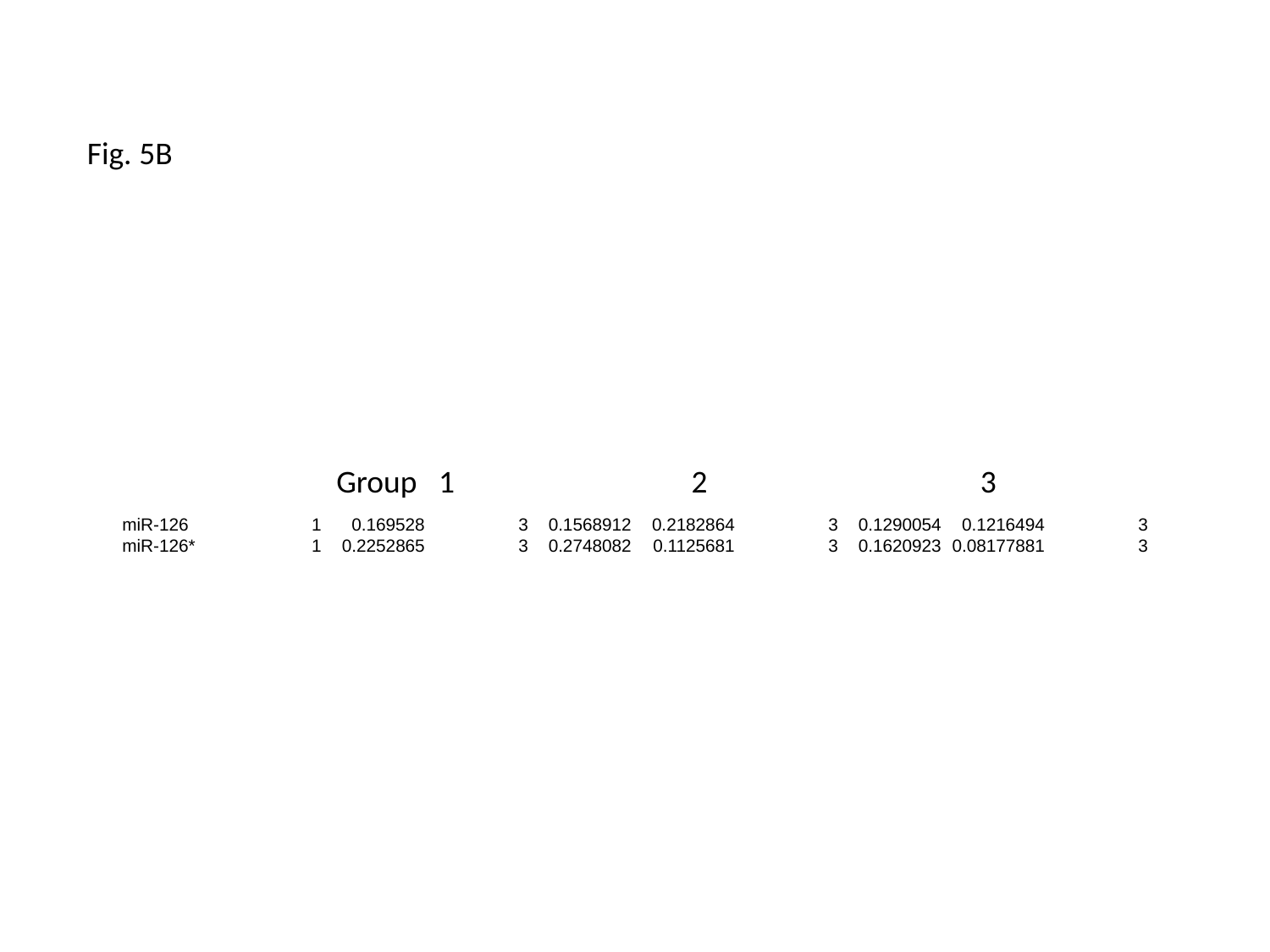

Fig. 5B
Group 1 2 3
| miR-126 | 1 | 0.169528 | 3 | 0.1568912 | 0.2182864 | 3 | 0.1290054 | 0.1216494 | 3 |
| --- | --- | --- | --- | --- | --- | --- | --- | --- | --- |
| miR-126\* | 1 | 0.2252865 | 3 | 0.2748082 | 0.1125681 | 3 | 0.1620923 | 0.08177881 | 3 |

## Slide 3
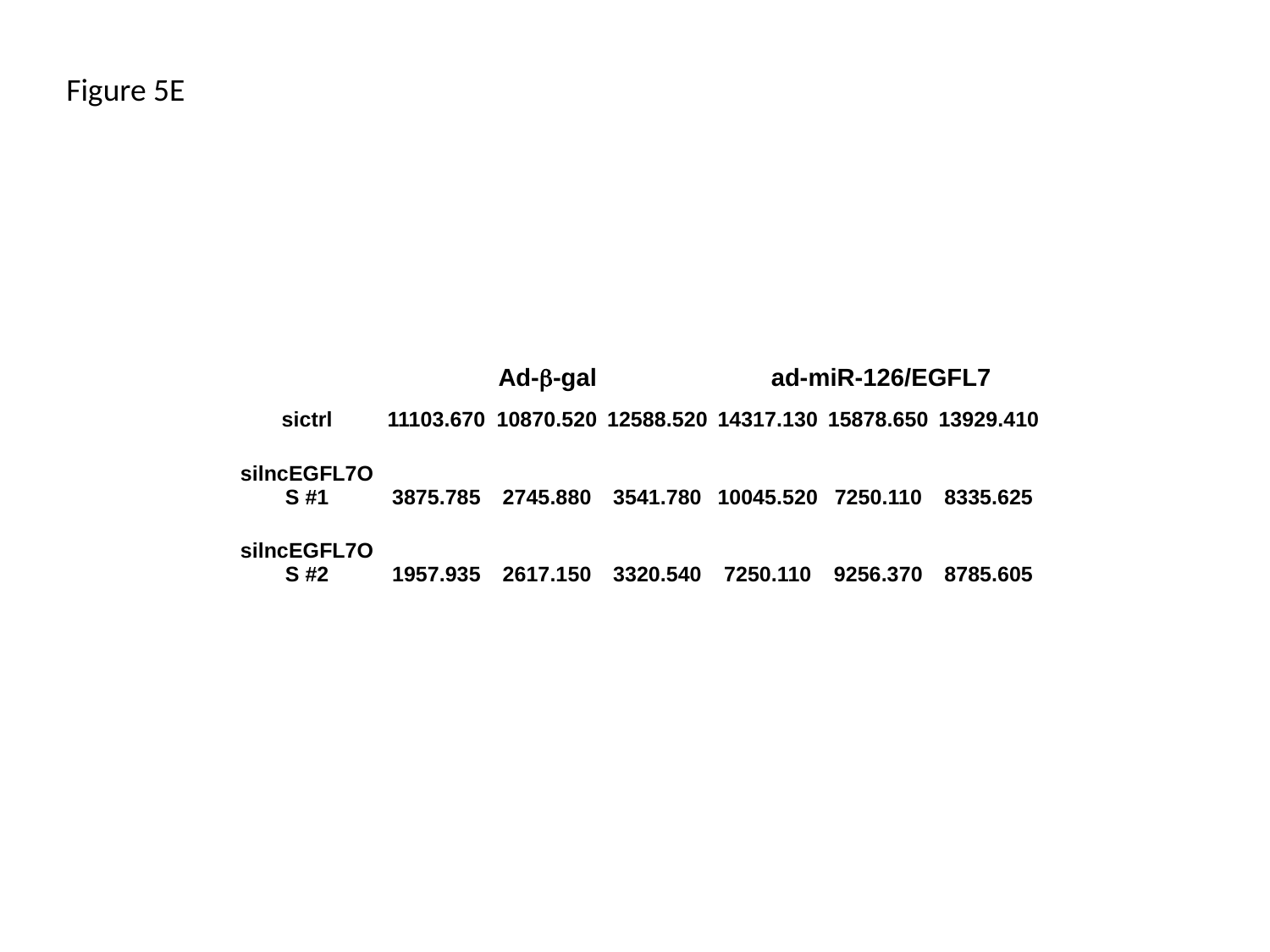

Figure 5E
| Ad-β-gal | ad-miR-126/EGFL7 |
| --- | --- |
| sictrl | 11103.670 | 10870.520 | 12588.520 | 14317.130 | 15878.650 | 13929.410 |
| --- | --- | --- | --- | --- | --- | --- |
| silncEGFL7OS #1 | 3875.785 | 2745.880 | 3541.780 | 10045.520 | 7250.110 | 8335.625 |
| silncEGFL7OS #2 | 1957.935 | 2617.150 | 3320.540 | 7250.110 | 9256.370 | 8785.605 |
