# Supplementary material for: LncEGFL7OS regulates human angiogenesis by interacting with MAX at the EGFL7/miR-126 locus
Source: eLife. 2019 Feb 11;8:e40470. doi: 10.7554/eLife.40470 (PMC6370342; doi:10.7554/eLife.40470)
Supplement: Figure 5—figure supplement 2—source data 1. [file elife-40470-fig5-figsupp2-data1.pptx]

## Slide 1
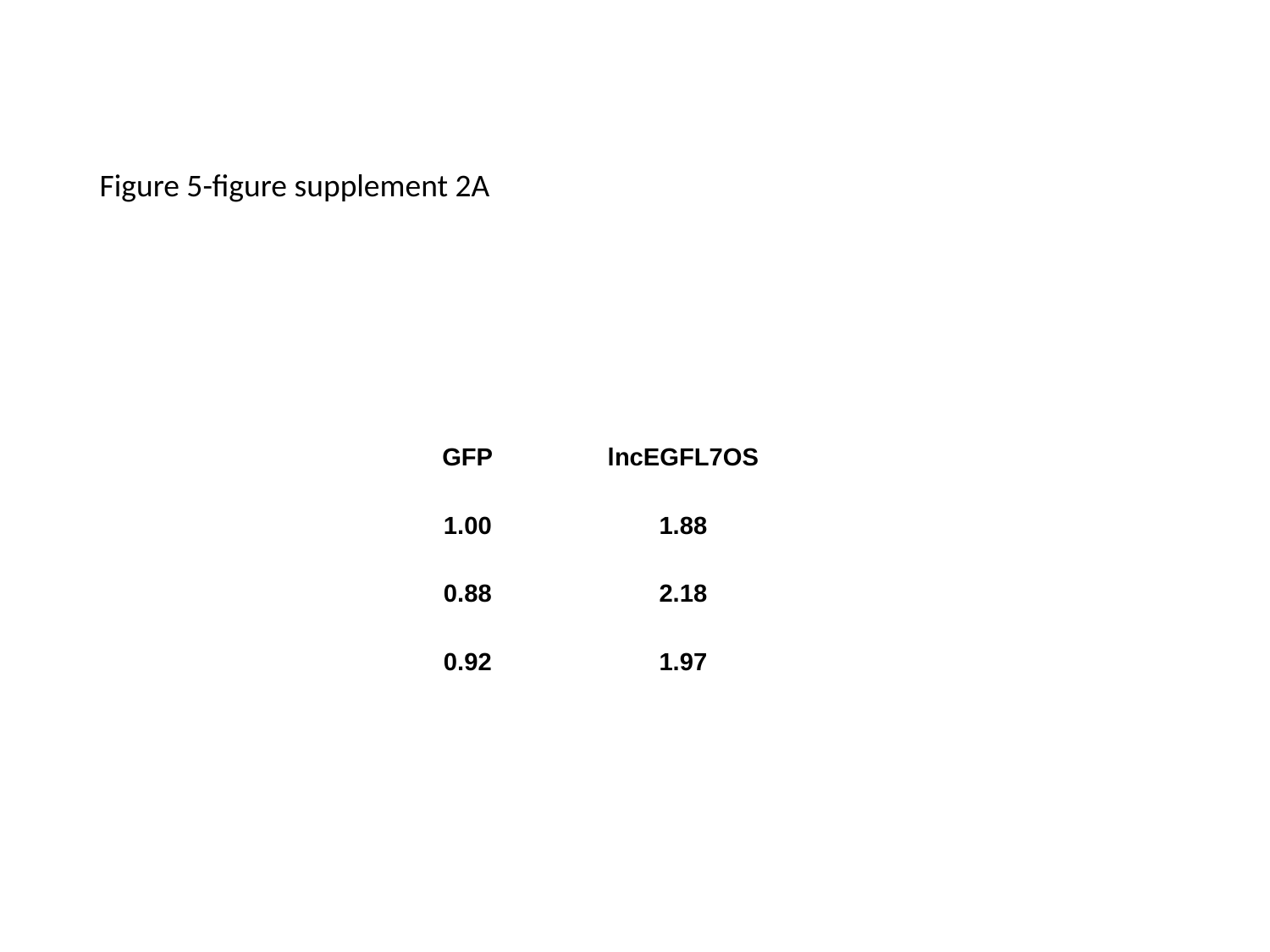

Figure 5-figure supplement 2A
| GFP | lncEGFL7OS |
| --- | --- |
| 1.00 | 1.88 |
| 0.88 | 2.18 |
| 0.92 | 1.97 |

## Slide 2
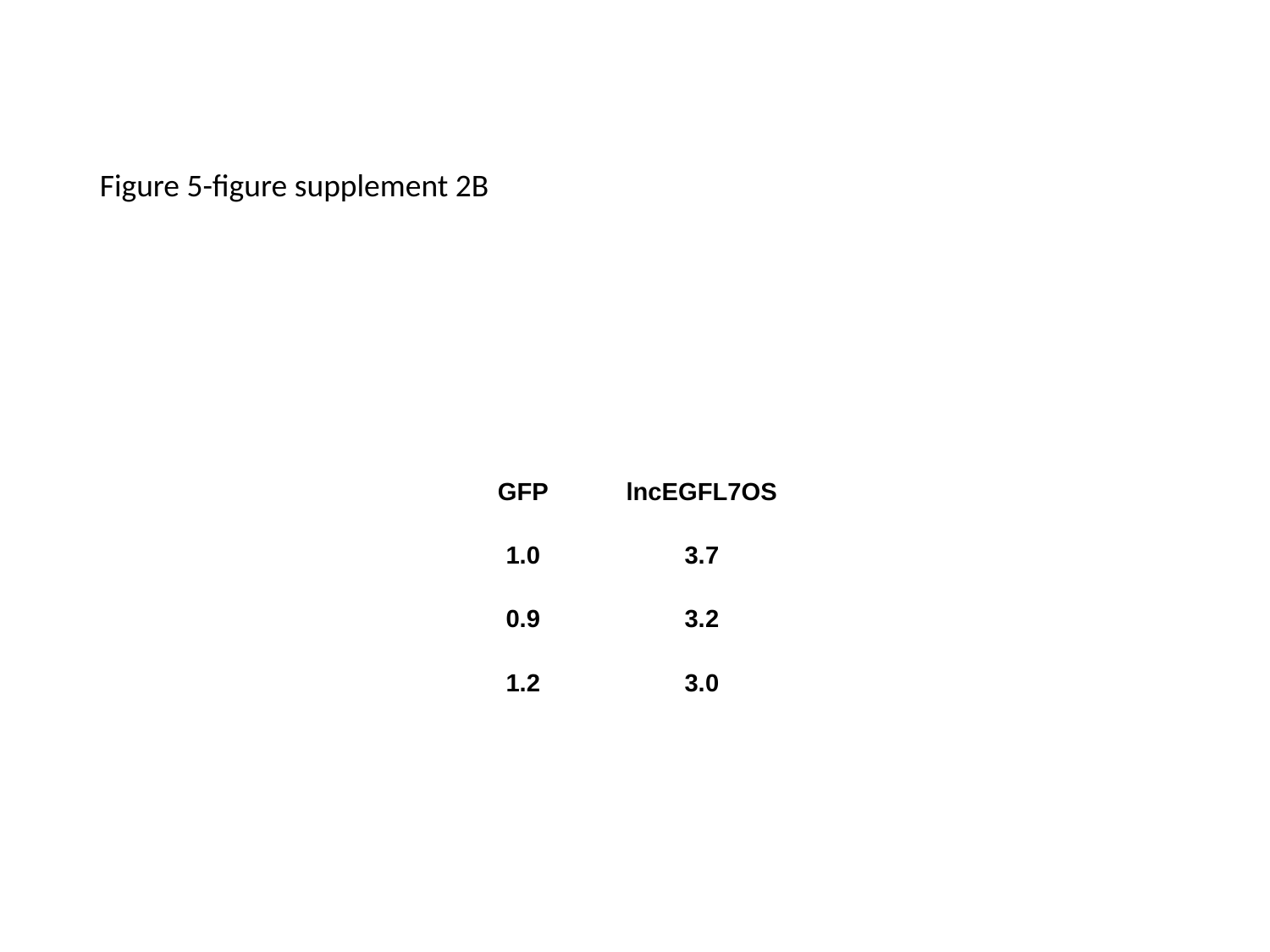

Figure 5-figure supplement 2B
| GFP | lncEGFL7OS |
| --- | --- |
| 1.0 | 3.7 |
| 0.9 | 3.2 |
| 1.2 | 3.0 |
