# Supplementary material for: LncEGFL7OS regulates human angiogenesis by interacting with MAX at the EGFL7/miR-126 locus
Source: eLife. 2019 Feb 11;8:e40470. doi: 10.7554/eLife.40470 (PMC6370342; doi:10.7554/eLife.40470)
Supplement: Figure 6—source data 1. [file elife-40470-fig6-data1.pptx]

## Slide 1
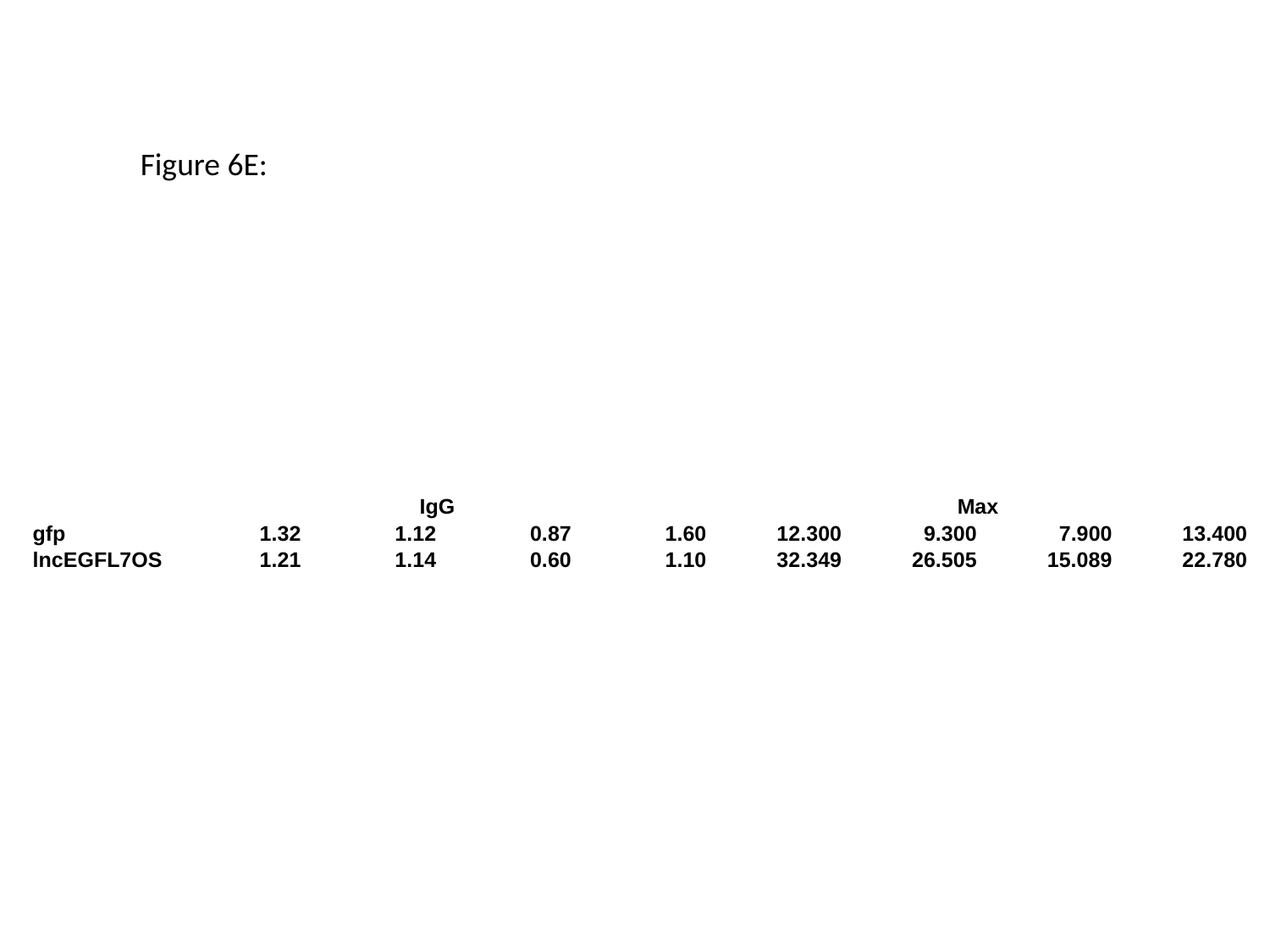

Figure 6E:
| | IgG | | | | Max | | | |
| --- | --- | --- | --- | --- | --- | --- | --- | --- |
| gfp | 1.32 | 1.12 | 0.87 | 1.60 | 12.300 | 9.300 | 7.900 | 13.400 |
| lncEGFL7OS | 1.21 | 1.14 | 0.60 | 1.10 | 32.349 | 26.505 | 15.089 | 22.780 |

## Slide 2
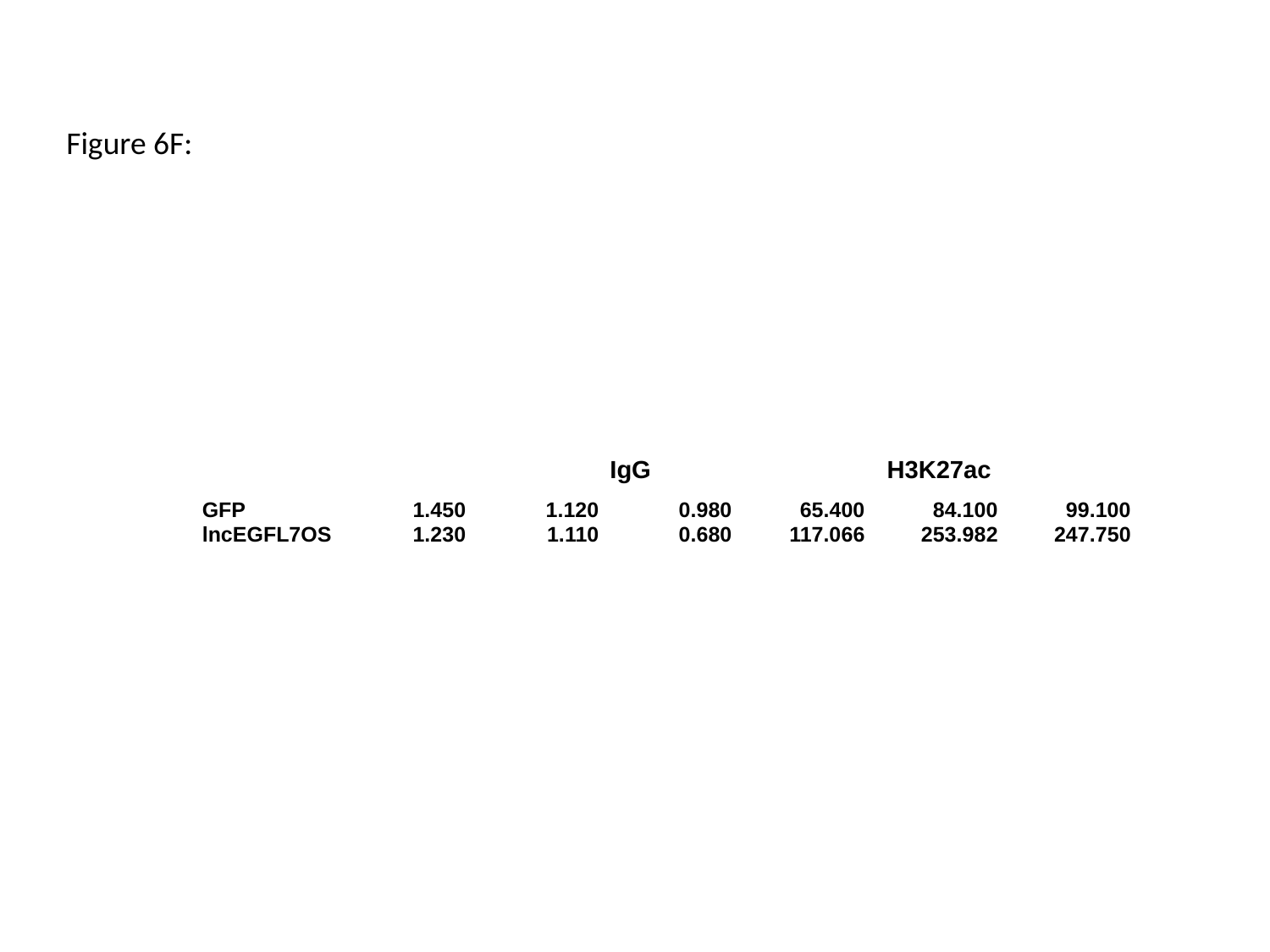

Figure 6F:
| IgG | H3K27ac |
| --- | --- |
| GFP | 1.450 | 1.120 | 0.980 | 65.400 | 84.100 | 99.100 |
| --- | --- | --- | --- | --- | --- | --- |
| lncEGFL7OS | 1.230 | 1.110 | 0.680 | 117.066 | 253.982 | 247.750 |

## Slide 3
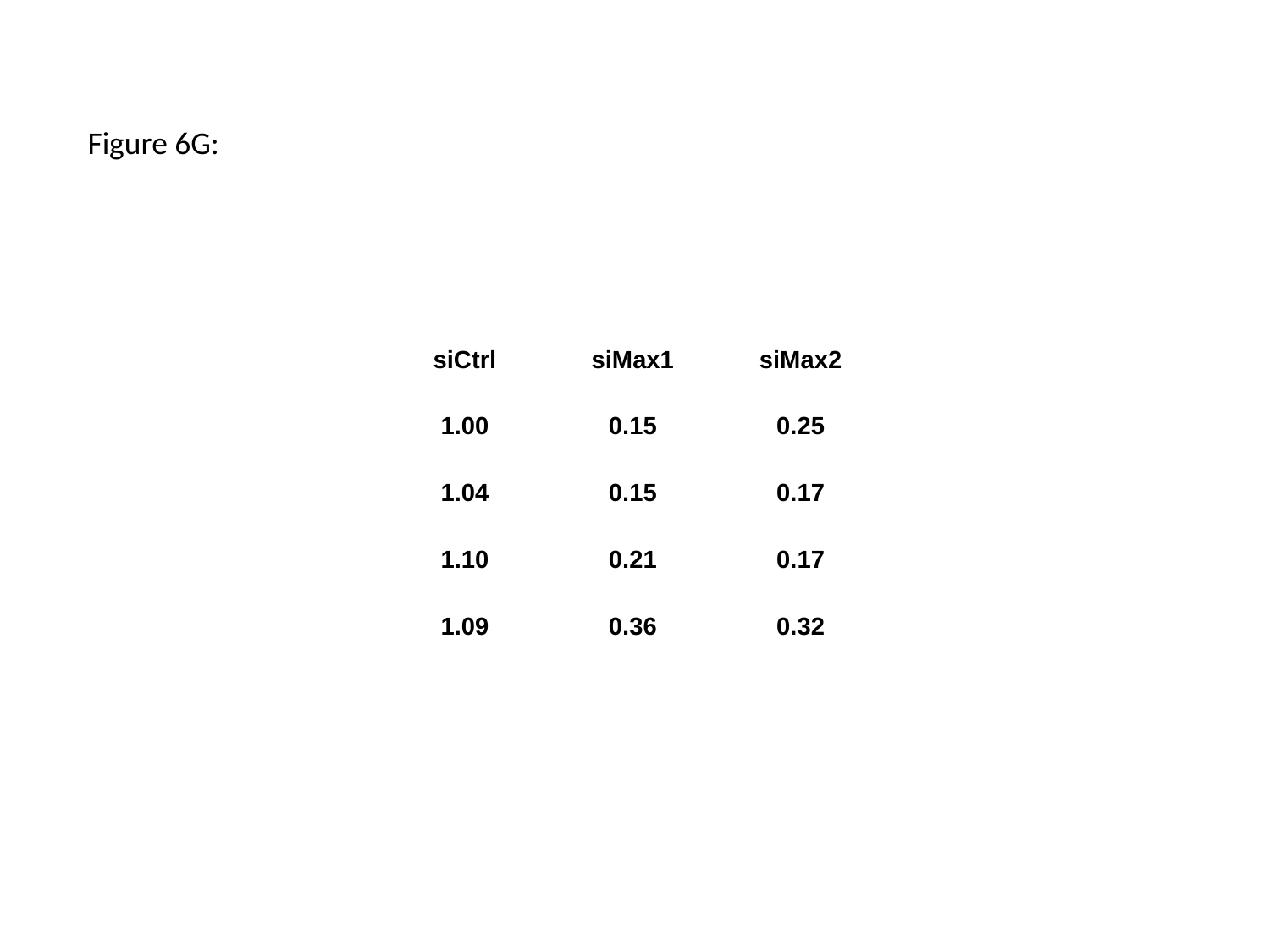

Figure 6G:
| siCtrl | siMax1 | siMax2 |
| --- | --- | --- |
| 1.00 | 0.15 | 0.25 |
| 1.04 | 0.15 | 0.17 |
| 1.10 | 0.21 | 0.17 |
| 1.09 | 0.36 | 0.32 |

## Slide 4
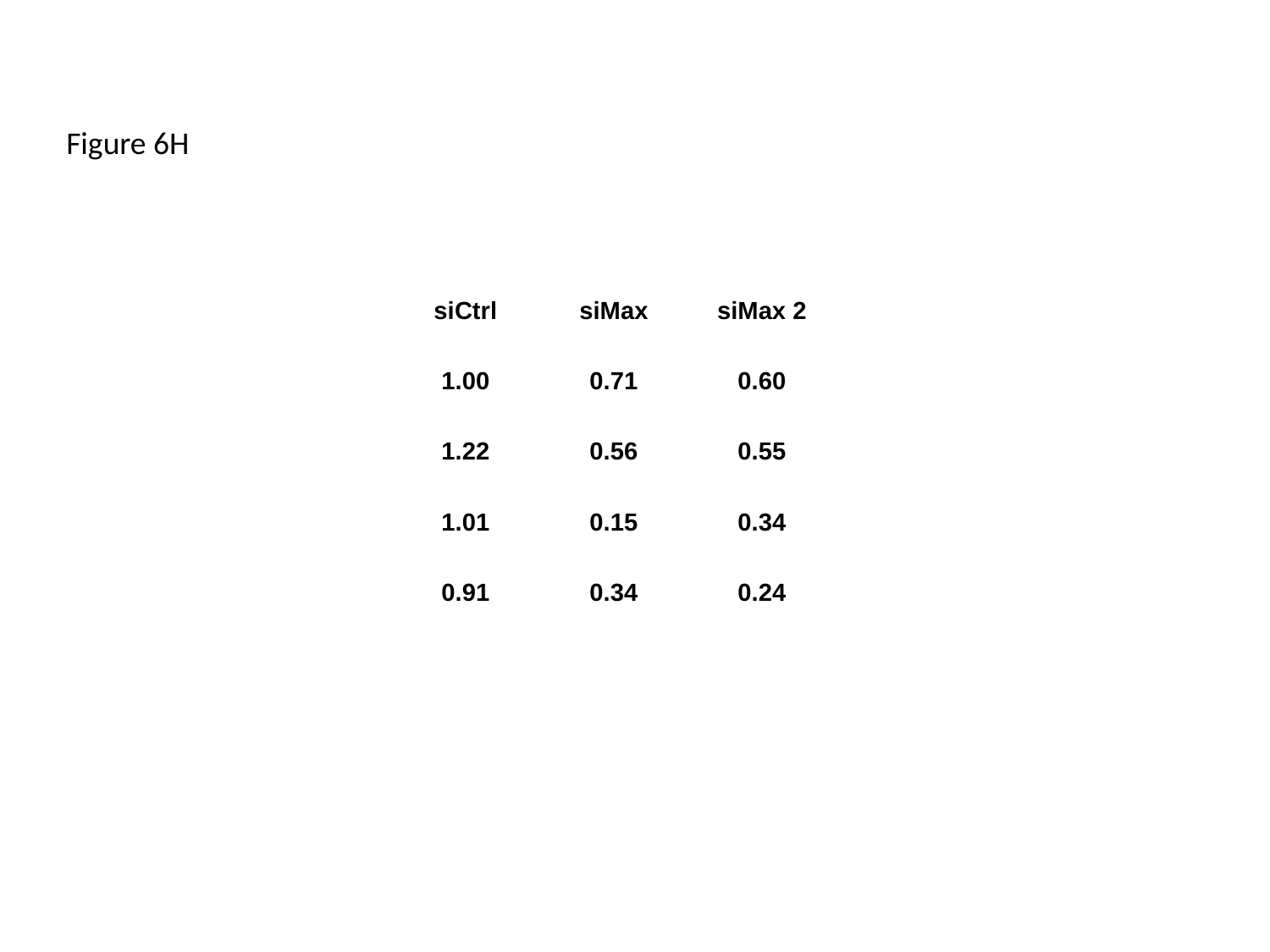

Figure 6H
| siCtrl | siMax | siMax 2 |
| --- | --- | --- |
| 1.00 | 0.71 | 0.60 |
| 1.22 | 0.56 | 0.55 |
| 1.01 | 0.15 | 0.34 |
| 0.91 | 0.34 | 0.24 |
| | | |

## Slide 5
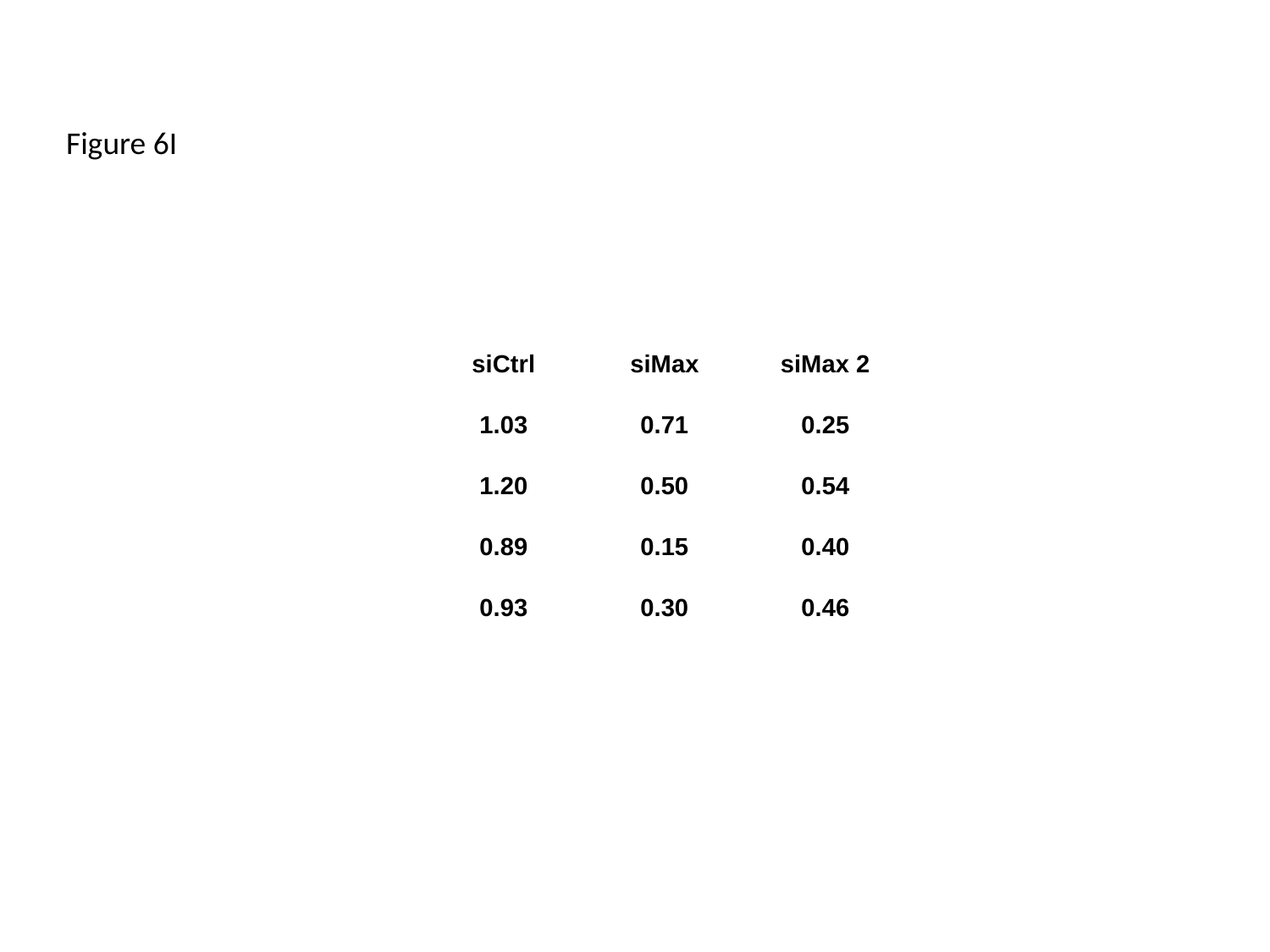

Figure 6I
| siCtrl | siMax | siMax 2 |
| --- | --- | --- |
| 1.03 | 0.71 | 0.25 |
| 1.20 | 0.50 | 0.54 |
| 0.89 | 0.15 | 0.40 |
| 0.93 | 0.30 | 0.46 |
| | | |

## Slide 6
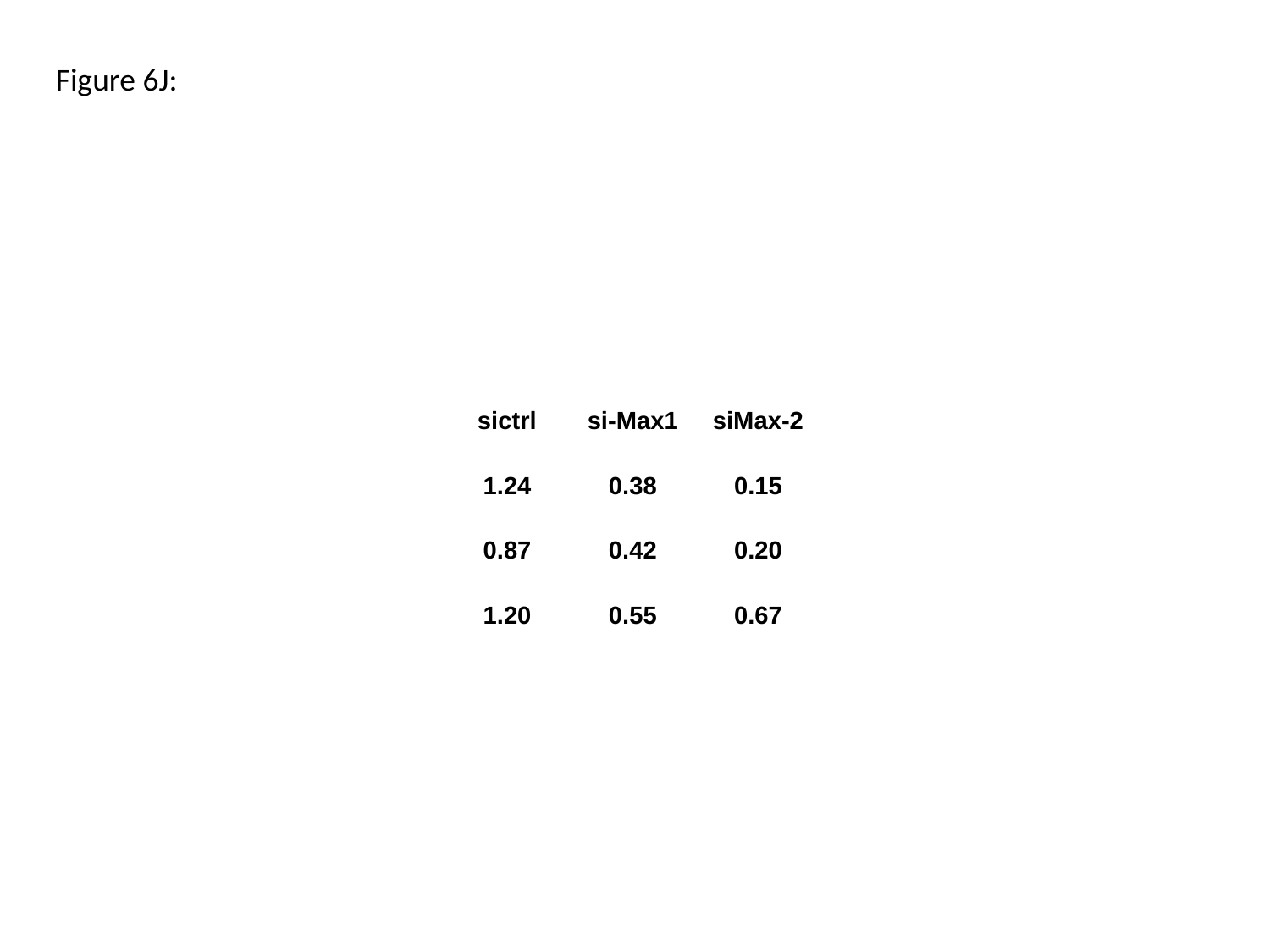

Figure 6J:
| sictrl | si-Max1 | siMax-2 |
| --- | --- | --- |
| 1.24 | 0.38 | 0.15 |
| 0.87 | 0.42 | 0.20 |
| 1.20 | 0.55 | 0.67 |

## Slide 7
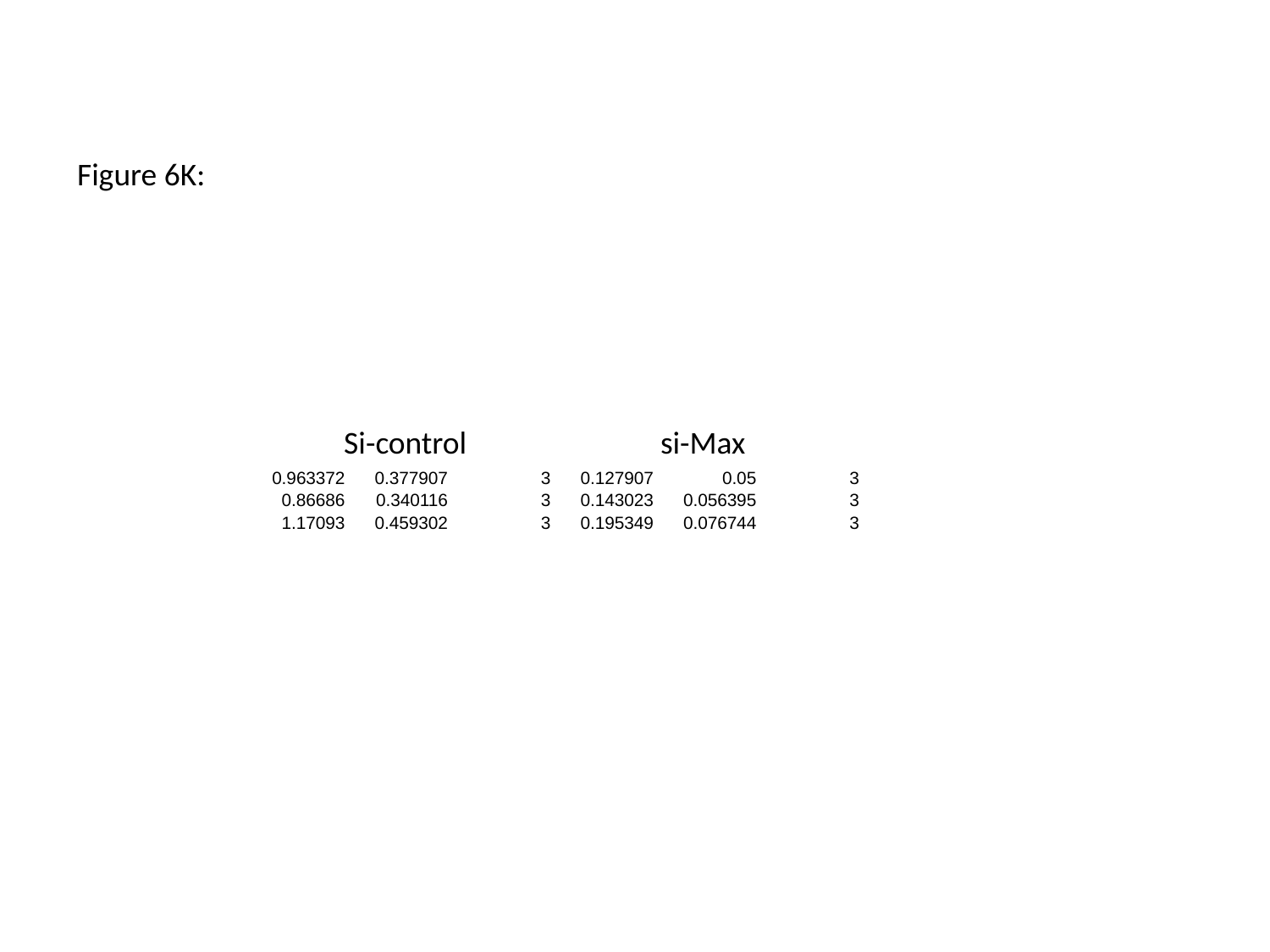

Figure 6K:
Si-control si-Max
| 0.963372 | 0.377907 | 3 | 0.127907 | 0.05 | 3 |
| --- | --- | --- | --- | --- | --- |
| 0.86686 | 0.340116 | 3 | 0.143023 | 0.056395 | 3 |
| 1.17093 | 0.459302 | 3 | 0.195349 | 0.076744 | 3 |

## Slide 8
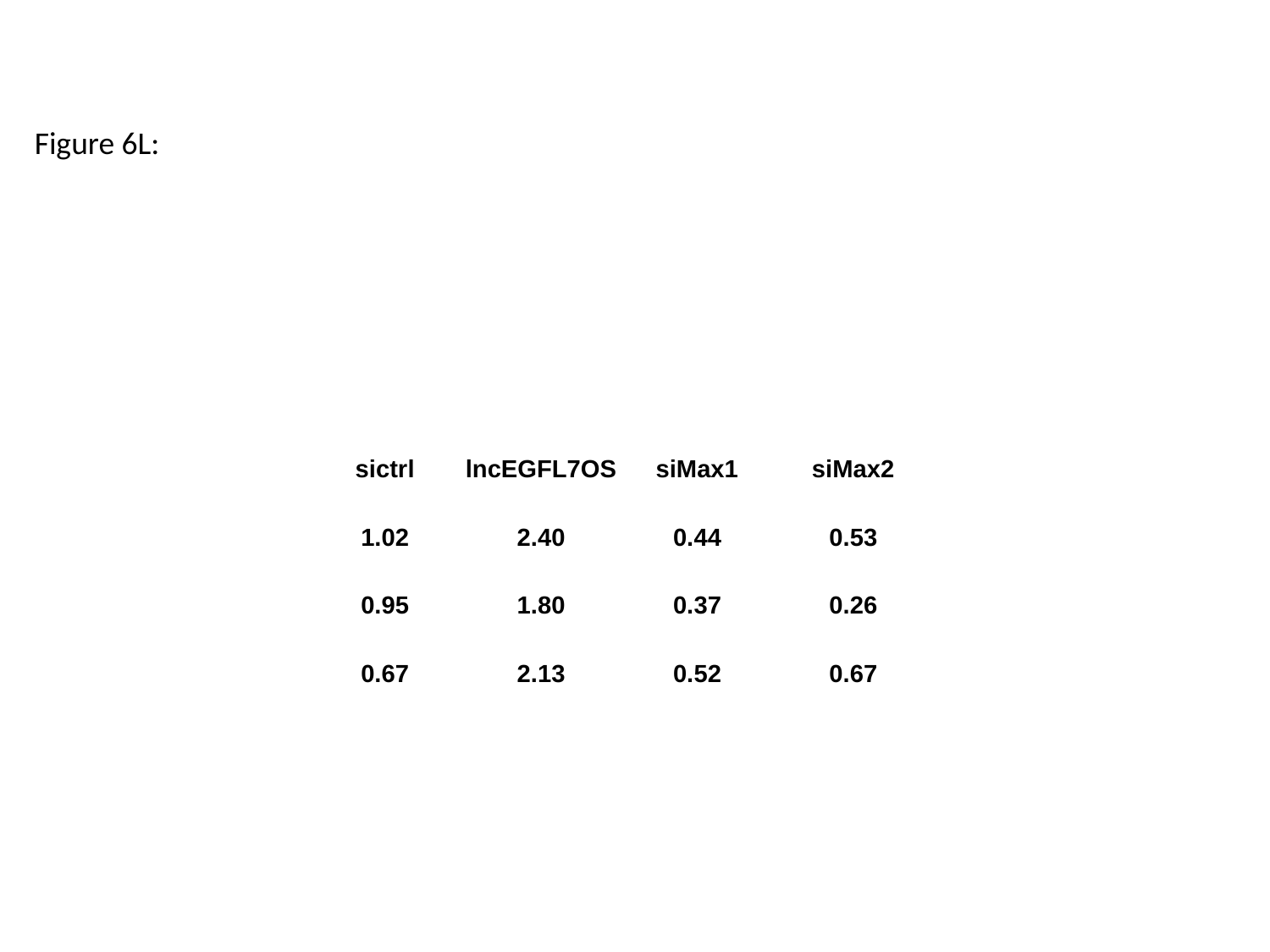

Figure 6L:
| sictrl | lncEGFL7OS | siMax1 | siMax2 |
| --- | --- | --- | --- |
| 1.02 | 2.40 | 0.44 | 0.53 |
| 0.95 | 1.80 | 0.37 | 0.26 |
| 0.67 | 2.13 | 0.52 | 0.67 |
