# Supplementary material for: LncEGFL7OS regulates human angiogenesis by interacting with MAX at the EGFL7/miR-126 locus
Source: eLife. 2019 Feb 11;8:e40470. doi: 10.7554/eLife.40470 (PMC6370342; doi:10.7554/eLife.40470)
Supplement: Figure 6—figure supplement 1—source data 1. [file elife-40470-fig6-figsupp1-data1.pptx]

## Slide 1
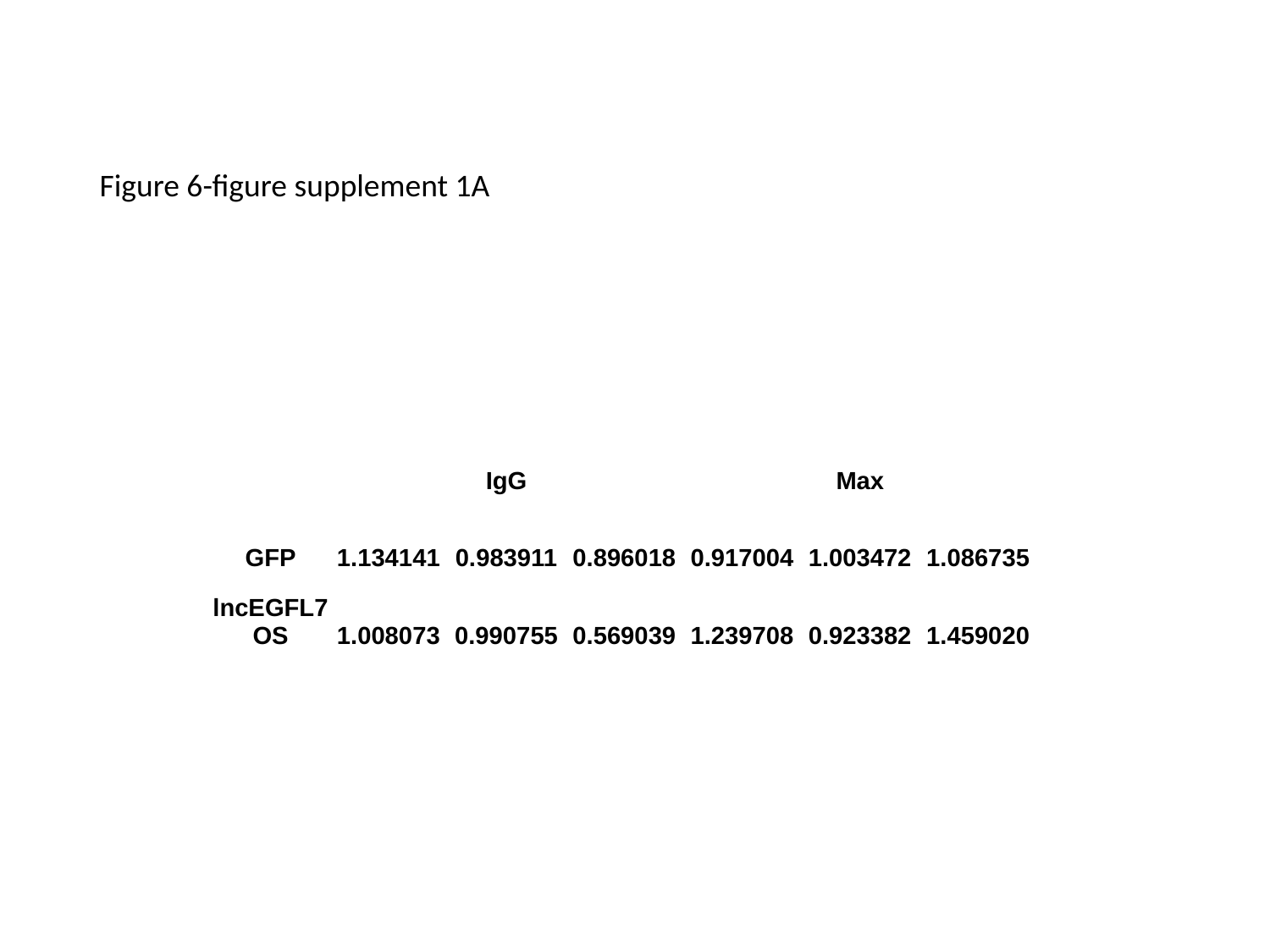

Figure 6-figure supplement 1A
| | IgG | | | Max | | |
| --- | --- | --- | --- | --- | --- | --- |
| GFP | 1.134141 | 0.983911 | 0.896018 | 0.917004 | 1.003472 | 1.086735 |
| lncEGFL7OS | 1.008073 | 0.990755 | 0.569039 | 1.239708 | 0.923382 | 1.459020 |

## Slide 2
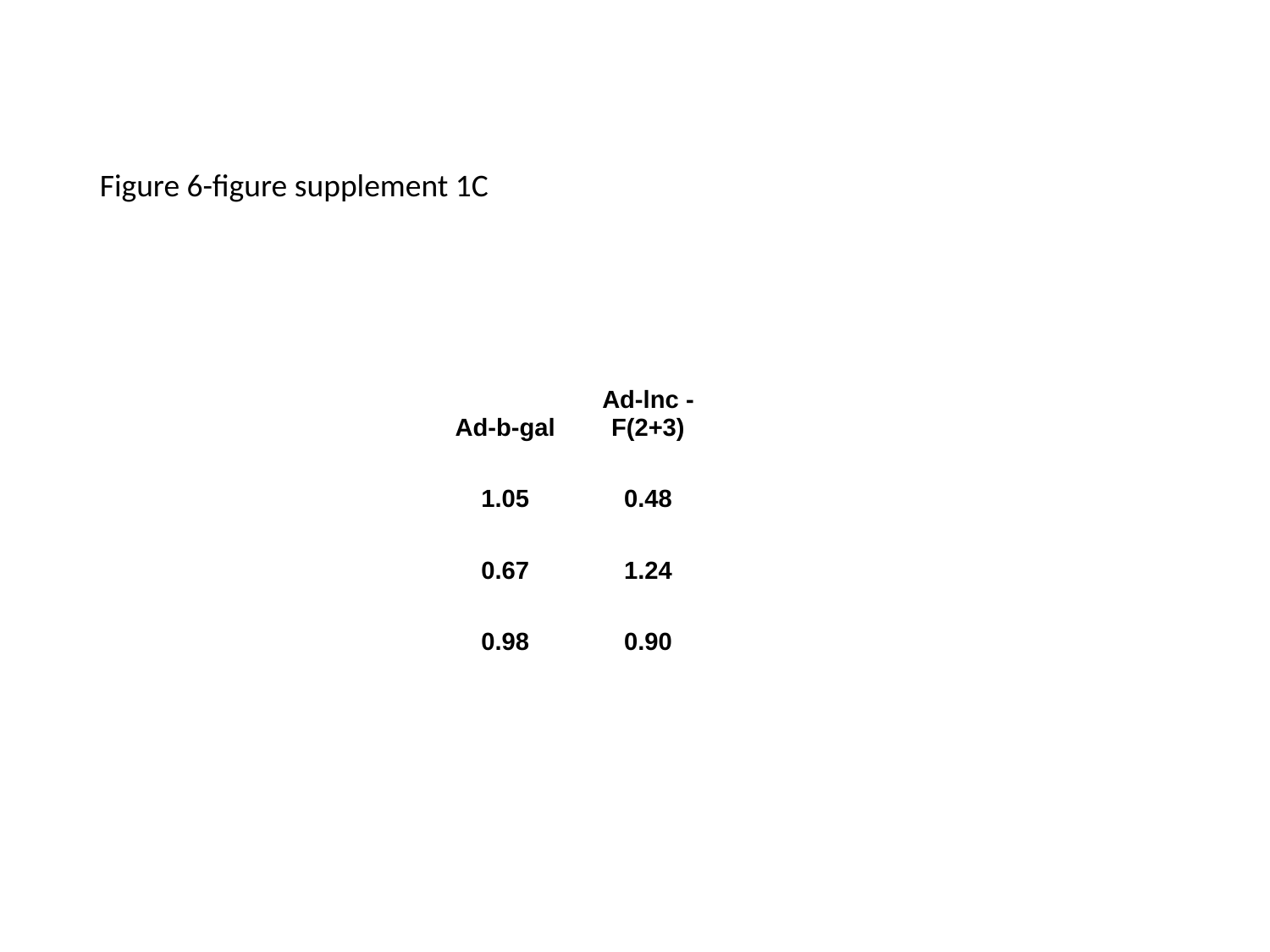

Figure 6-figure supplement 1C
| Ad-b-gal | Ad-lnc -F(2+3) |
| --- | --- |
| 1.05 | 0.48 |
| 0.67 | 1.24 |
| 0.98 | 0.90 |

## Slide 3
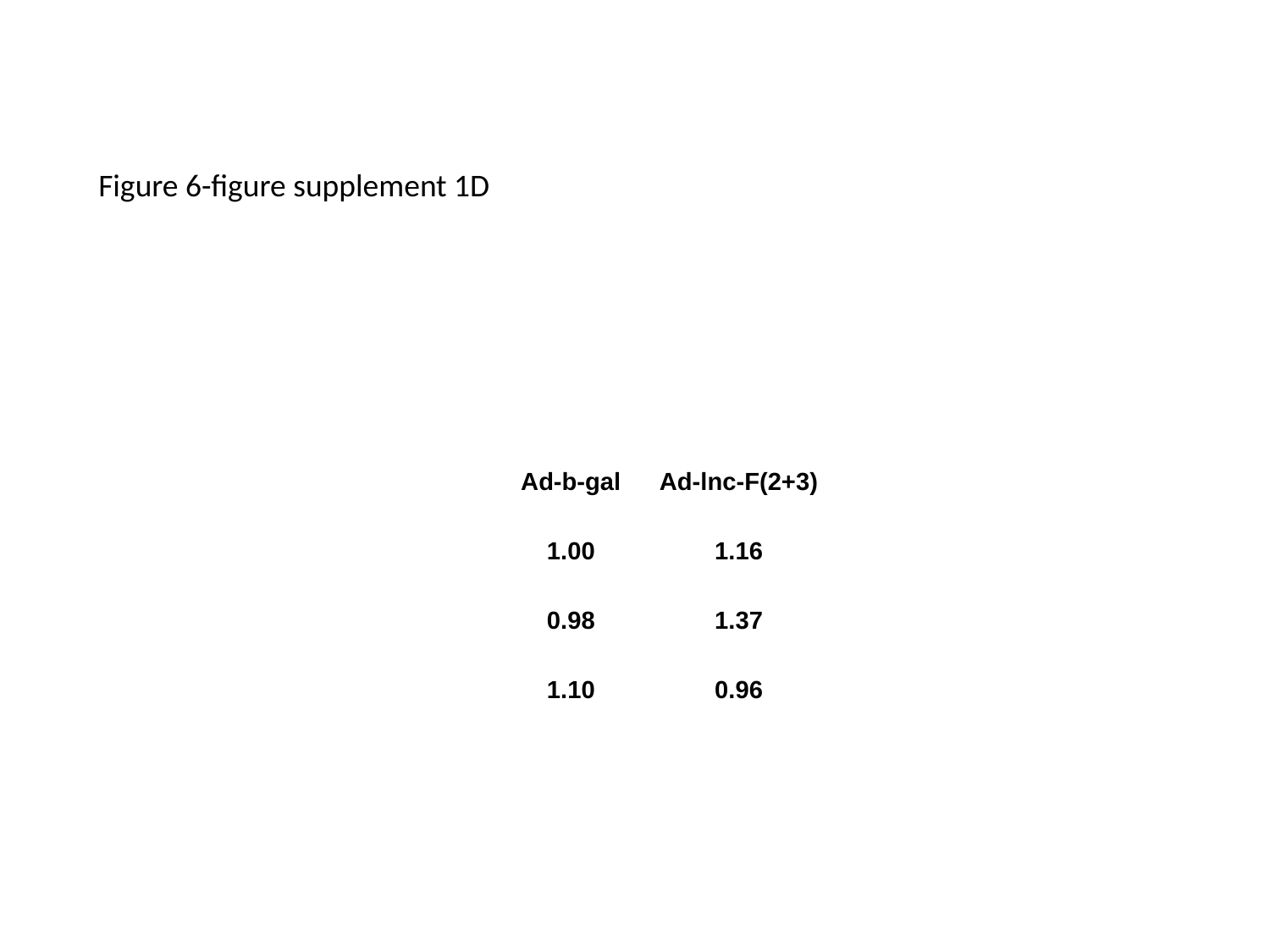

Figure 6-figure supplement 1D
| Ad-b-gal | Ad-lnc-F(2+3) |
| --- | --- |
| 1.00 | 1.16 |
| 0.98 | 1.37 |
| 1.10 | 0.96 |

## Slide 4
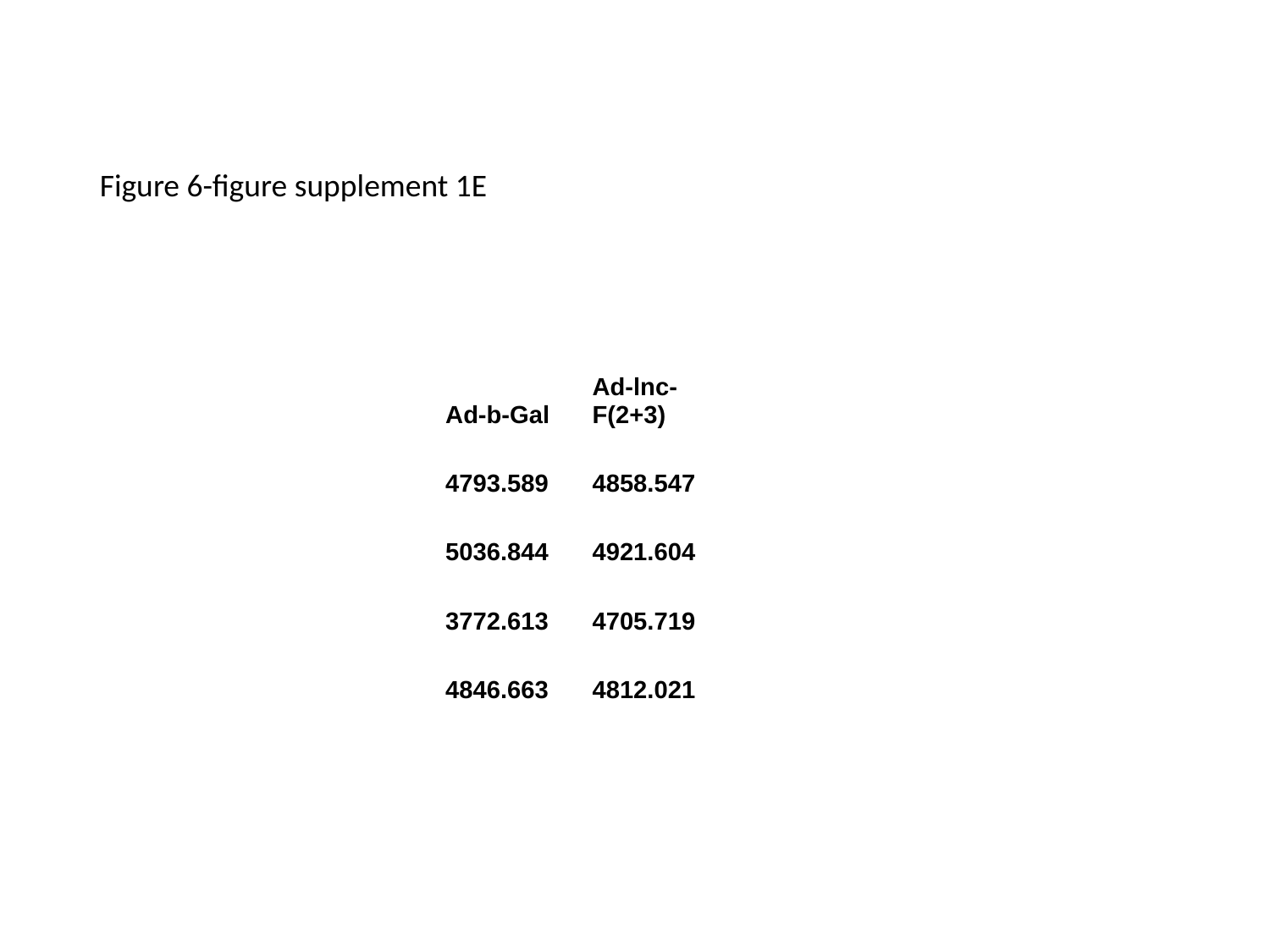

Figure 6-figure supplement 1E
| Ad-b-Gal | Ad-lnc-F(2+3) |
| --- | --- |
| 4793.589 | 4858.547 |
| 5036.844 | 4921.604 |
| 3772.613 | 4705.719 |
| 4846.663 | 4812.021 |
