# Supplementary material for: LncEGFL7OS regulates human angiogenesis by interacting with MAX at the EGFL7/miR-126 locus
Source: eLife. 2019 Feb 11;8:e40470. doi: 10.7554/eLife.40470 (PMC6370342; doi:10.7554/eLife.40470)
Supplement: Figure 7—source data 1. [file elife-40470-fig7-data1.pptx]

## Slide 1
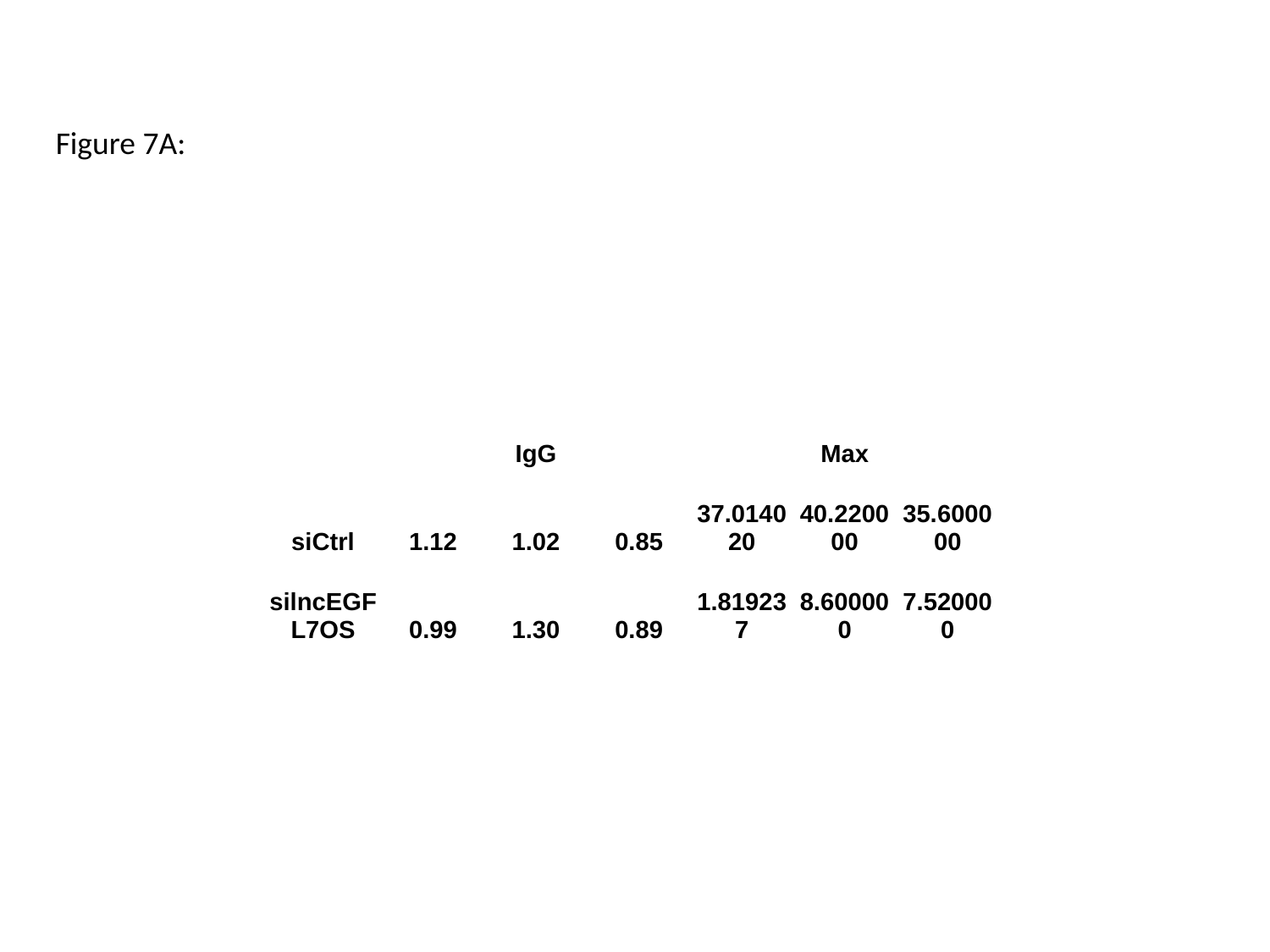

Figure 7A:
| | IgG | | | Max | | |
| --- | --- | --- | --- | --- | --- | --- |
| siCtrl | 1.12 | 1.02 | 0.85 | 37.014020 | 40.220000 | 35.600000 |
| silncEGFL7OS | 0.99 | 1.30 | 0.89 | 1.819237 | 8.600000 | 7.520000 |

## Slide 2
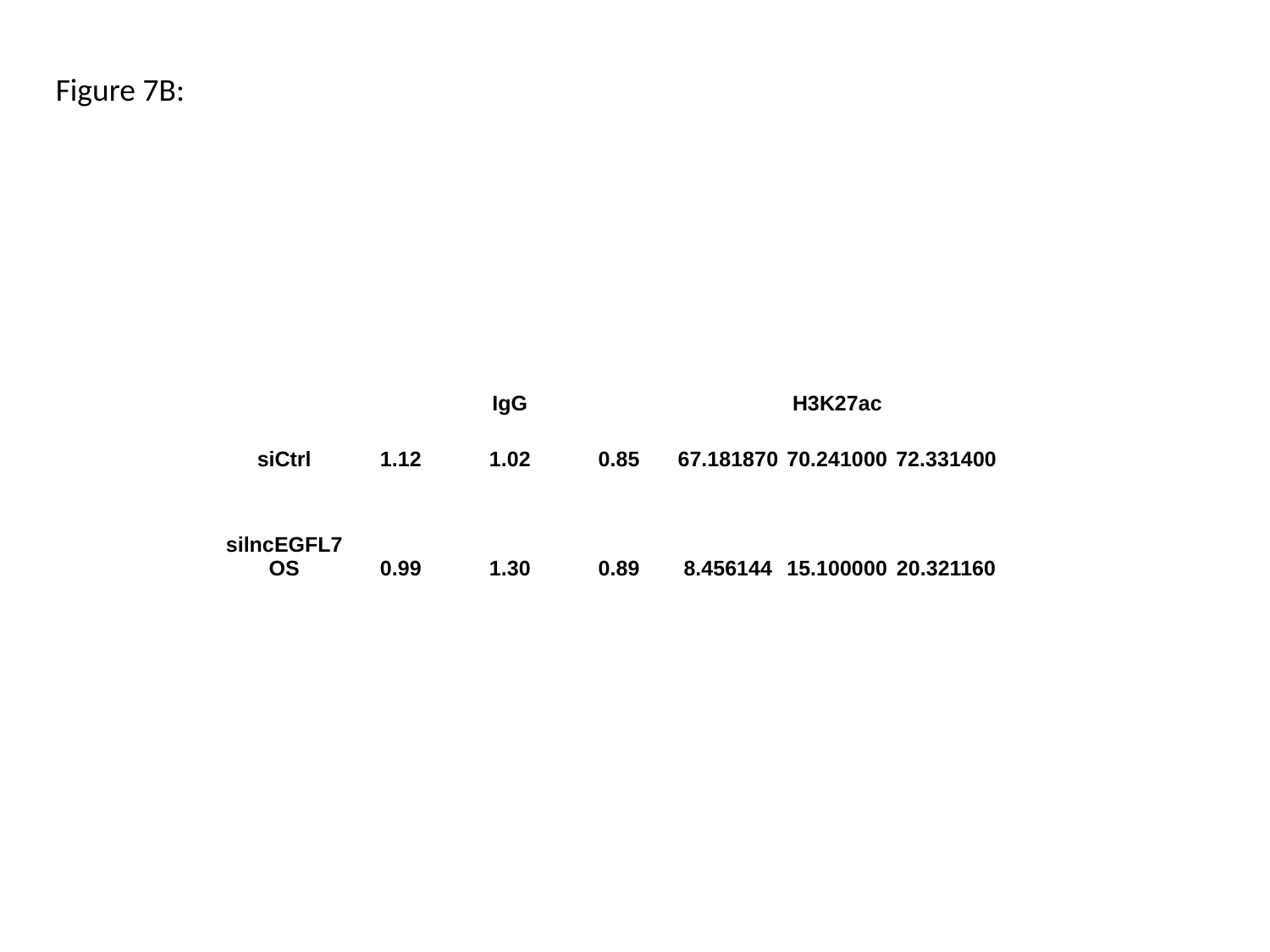

Figure 7B:
| | IgG | | | H3K27ac | | |
| --- | --- | --- | --- | --- | --- | --- |
| siCtrl | 1.12 | 1.02 | 0.85 | 67.181870 | 70.241000 | 72.331400 |
| silncEGFL7OS | 0.99 | 1.30 | 0.89 | 8.456144 | 15.100000 | 20.321160 |

## Slide 3
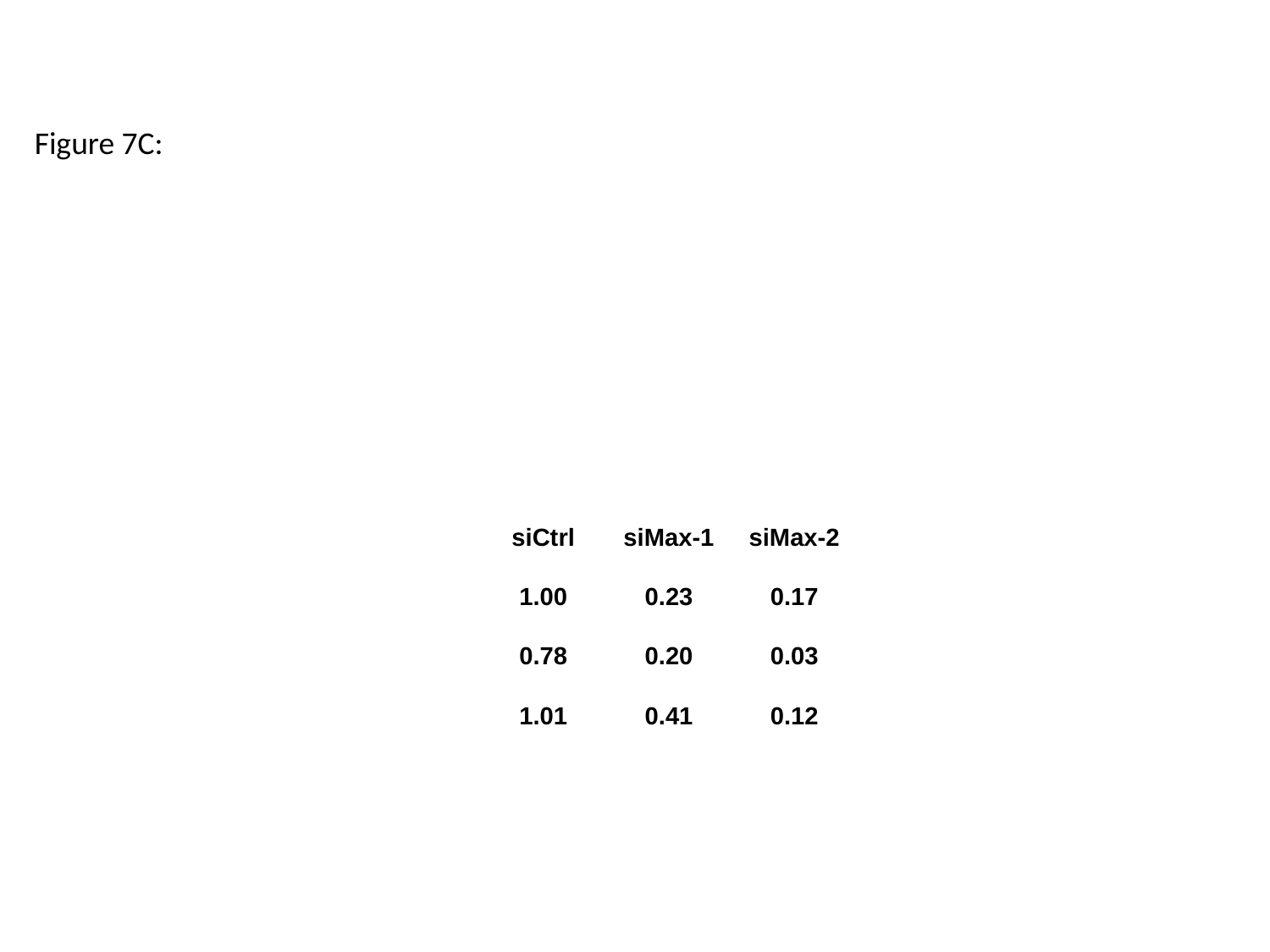

Figure 7C:
| siCtrl | siMax-1 | siMax-2 |
| --- | --- | --- |
| 1.00 | 0.23 | 0.17 |
| 0.78 | 0.20 | 0.03 |
| 1.01 | 0.41 | 0.12 |

## Slide 4
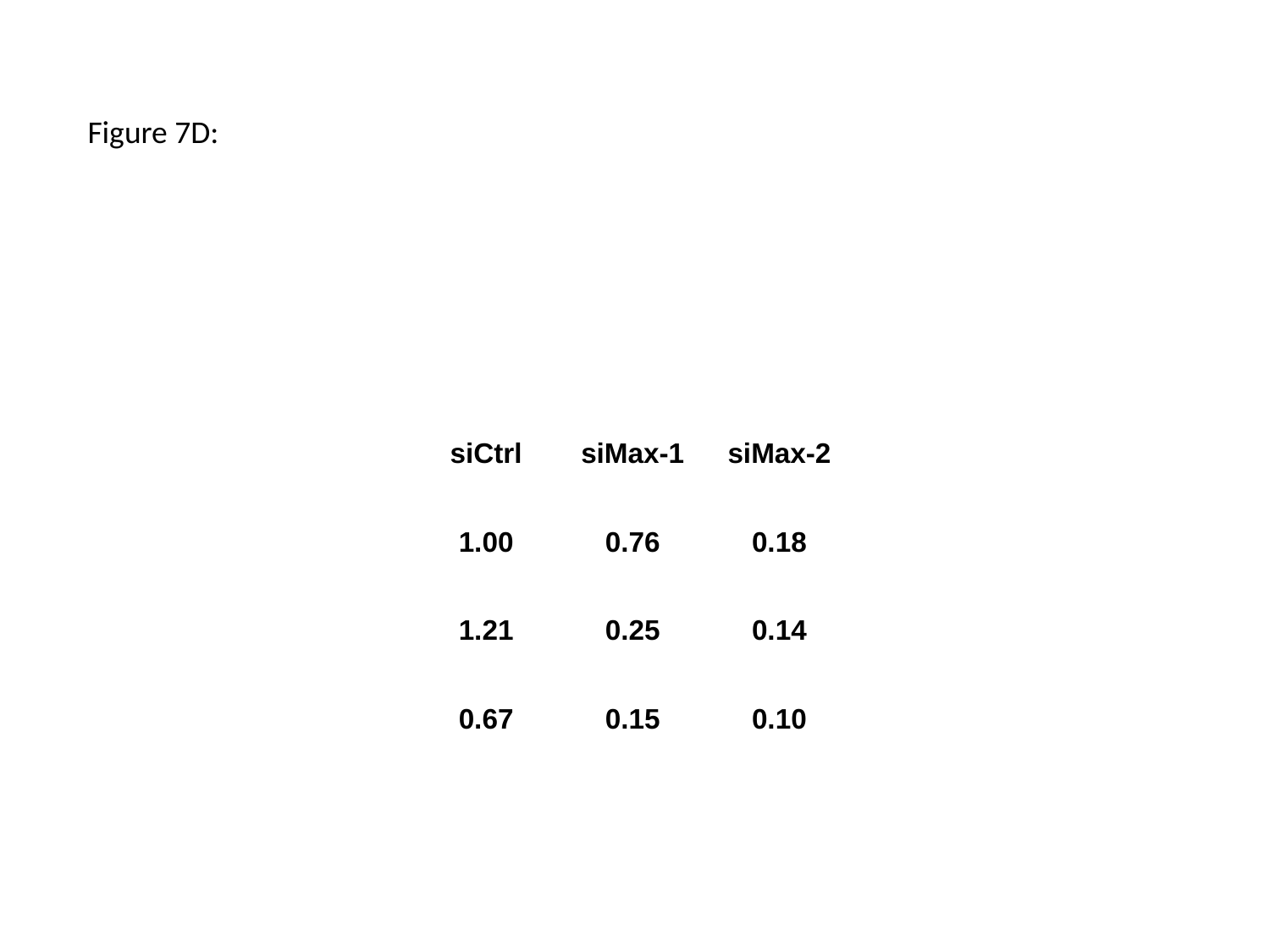

Figure 7D:
| siCtrl | siMax-1 | siMax-2 |
| --- | --- | --- |
| 1.00 | 0.76 | 0.18 |
| 1.21 | 0.25 | 0.14 |
| 0.67 | 0.15 | 0.10 |

## Slide 5
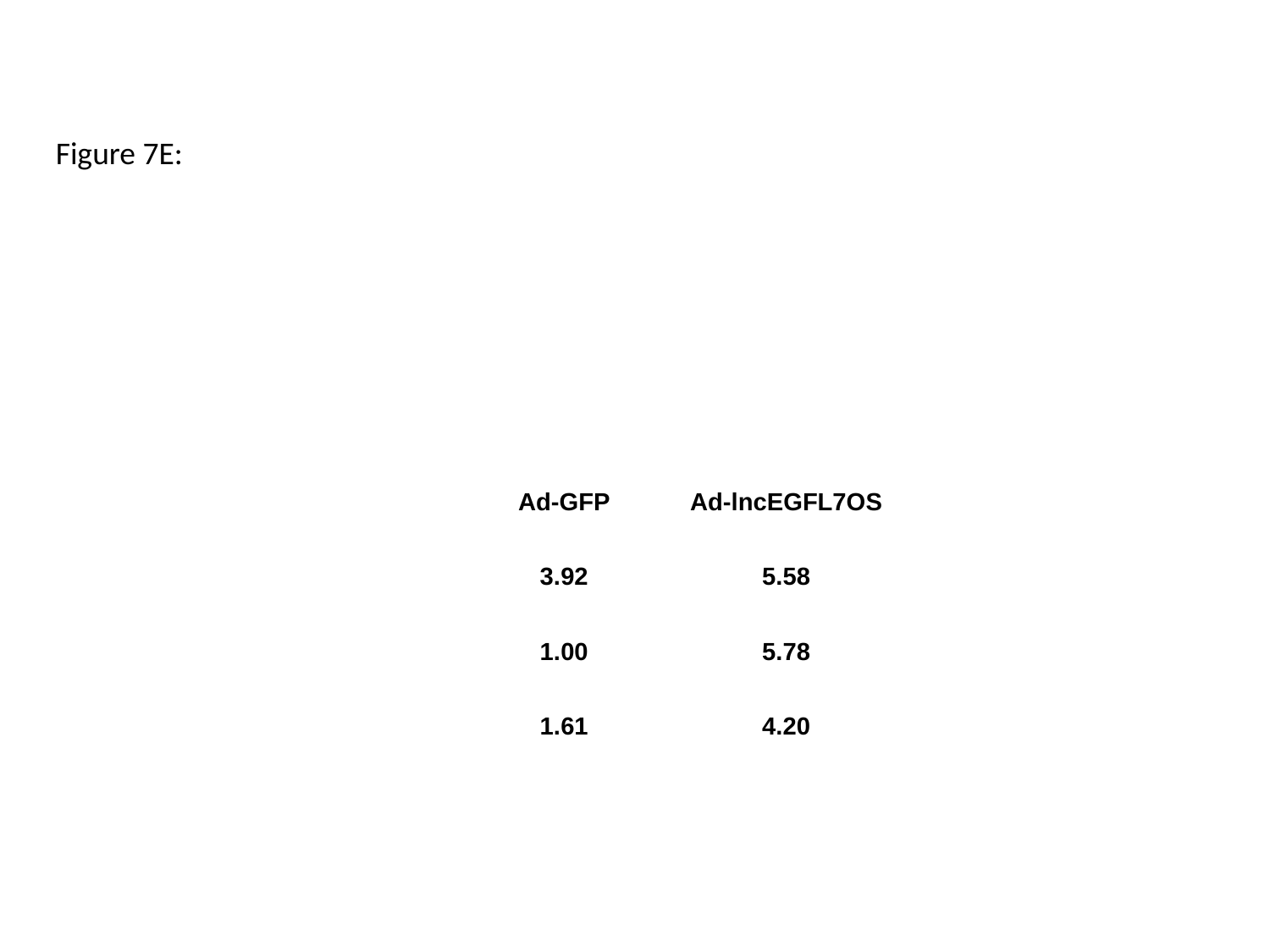

Figure 7E:
| Ad-GFP | Ad-lncEGFL7OS |
| --- | --- |
| 3.92 | 5.58 |
| 1.00 | 5.78 |
| 1.61 | 4.20 |

## Slide 6
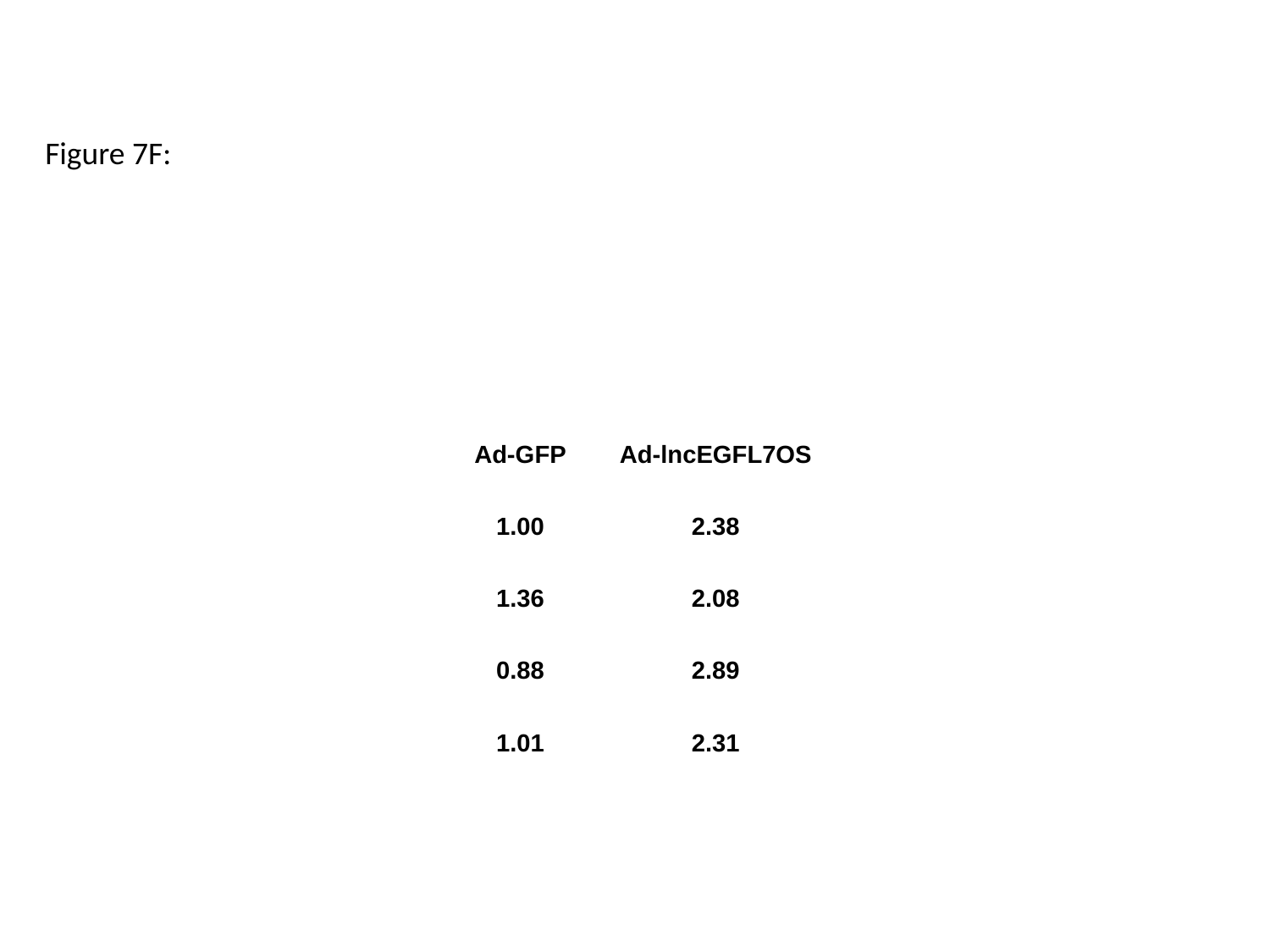

Figure 7F:
| Ad-GFP | Ad-lncEGFL7OS |
| --- | --- |
| 1.00 | 2.38 |
| 1.36 | 2.08 |
| 0.88 | 2.89 |
| 1.01 | 2.31 |

## Slide 7
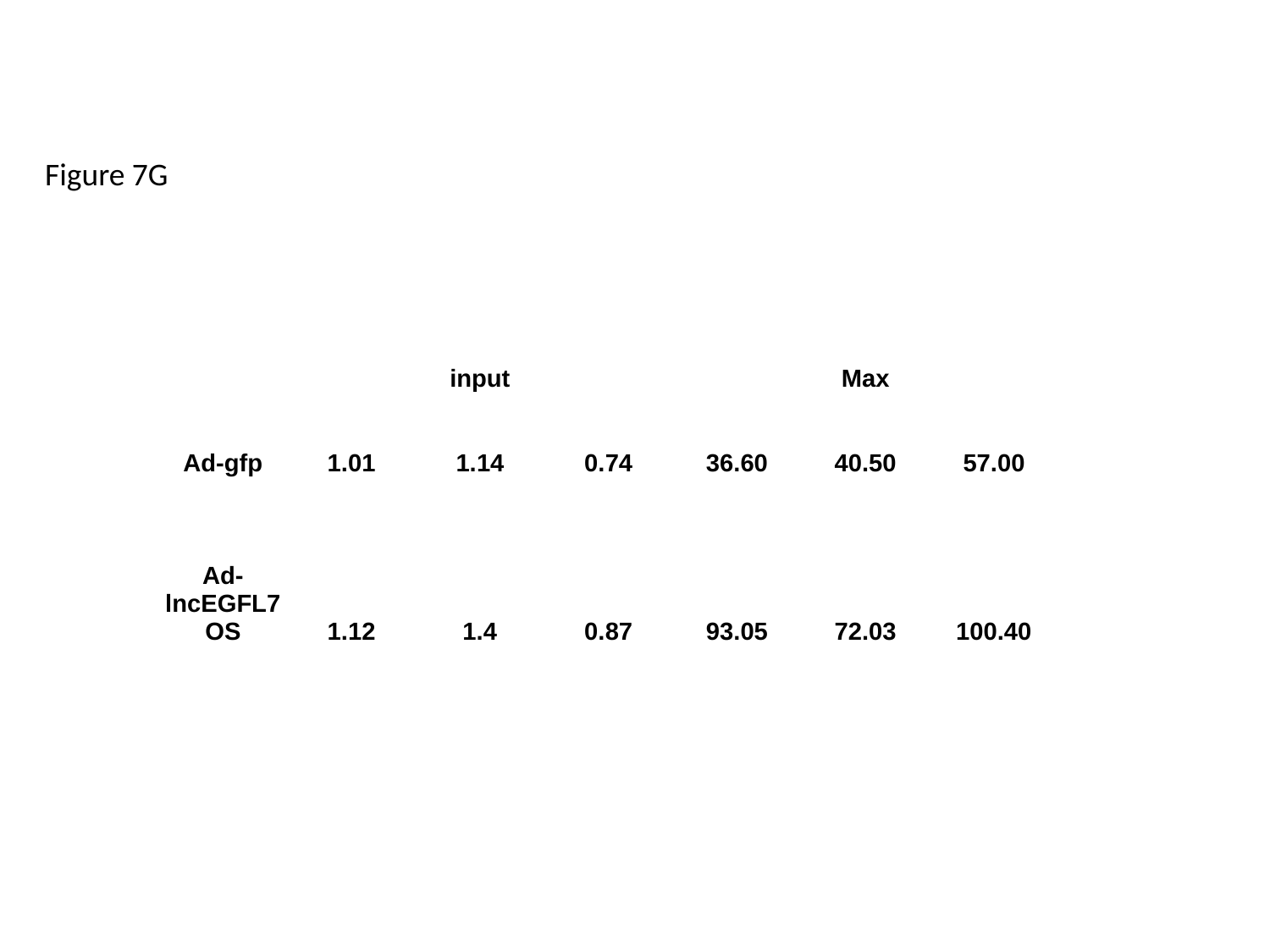

Figure 7G
| | input | | | Max | | |
| --- | --- | --- | --- | --- | --- | --- |
| Ad-gfp | 1.01 | 1.14 | 0.74 | 36.60 | 40.50 | 57.00 |
| Ad-lncEGFL7OS | 1.12 | 1.4 | 0.87 | 93.05 | 72.03 | 100.40 |

## Slide 8
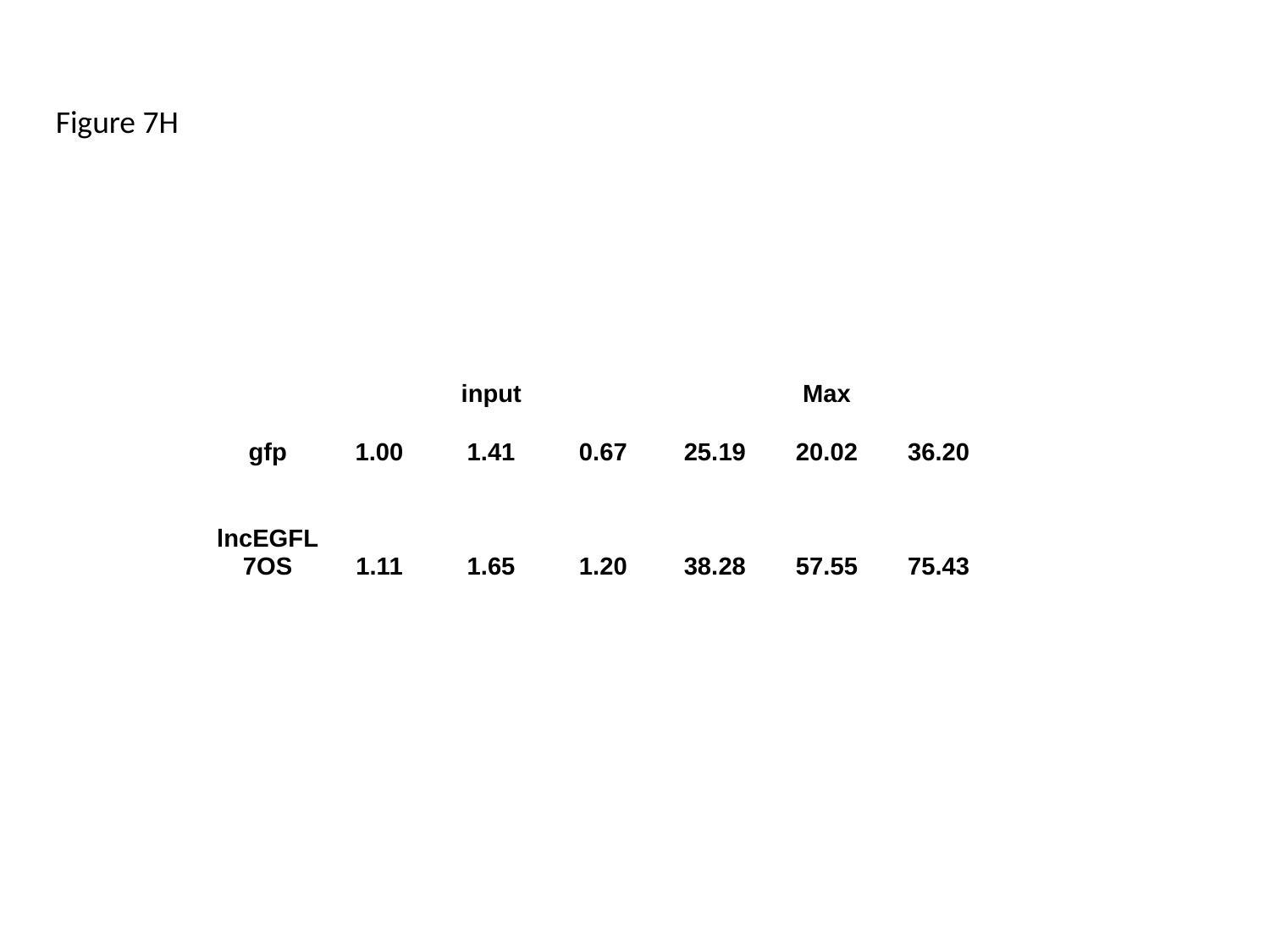

Figure 7H
| | input | | | Max | | |
| --- | --- | --- | --- | --- | --- | --- |
| gfp | 1.00 | 1.41 | 0.67 | 25.19 | 20.02 | 36.20 |
| lncEGFL7OS | 1.11 | 1.65 | 1.20 | 38.28 | 57.55 | 75.43 |

## Slide 9
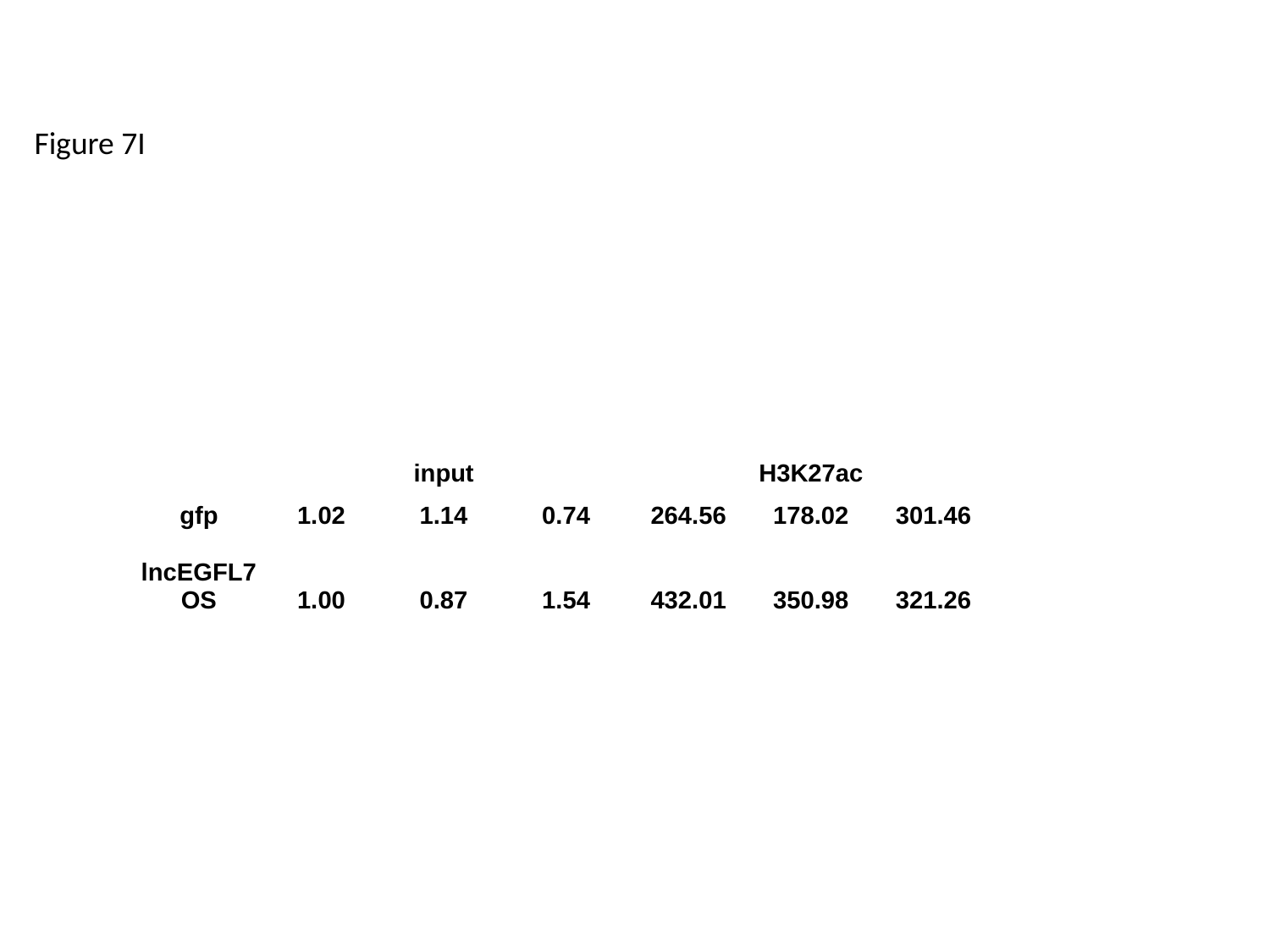

Figure 7I
| | input | | | H3K27ac | | |
| --- | --- | --- | --- | --- | --- | --- |
| gfp | 1.02 | 1.14 | 0.74 | 264.56 | 178.02 | 301.46 |
| lncEGFL7OS | 1.00 | 0.87 | 1.54 | 432.01 | 350.98 | 321.26 |

## Slide 10
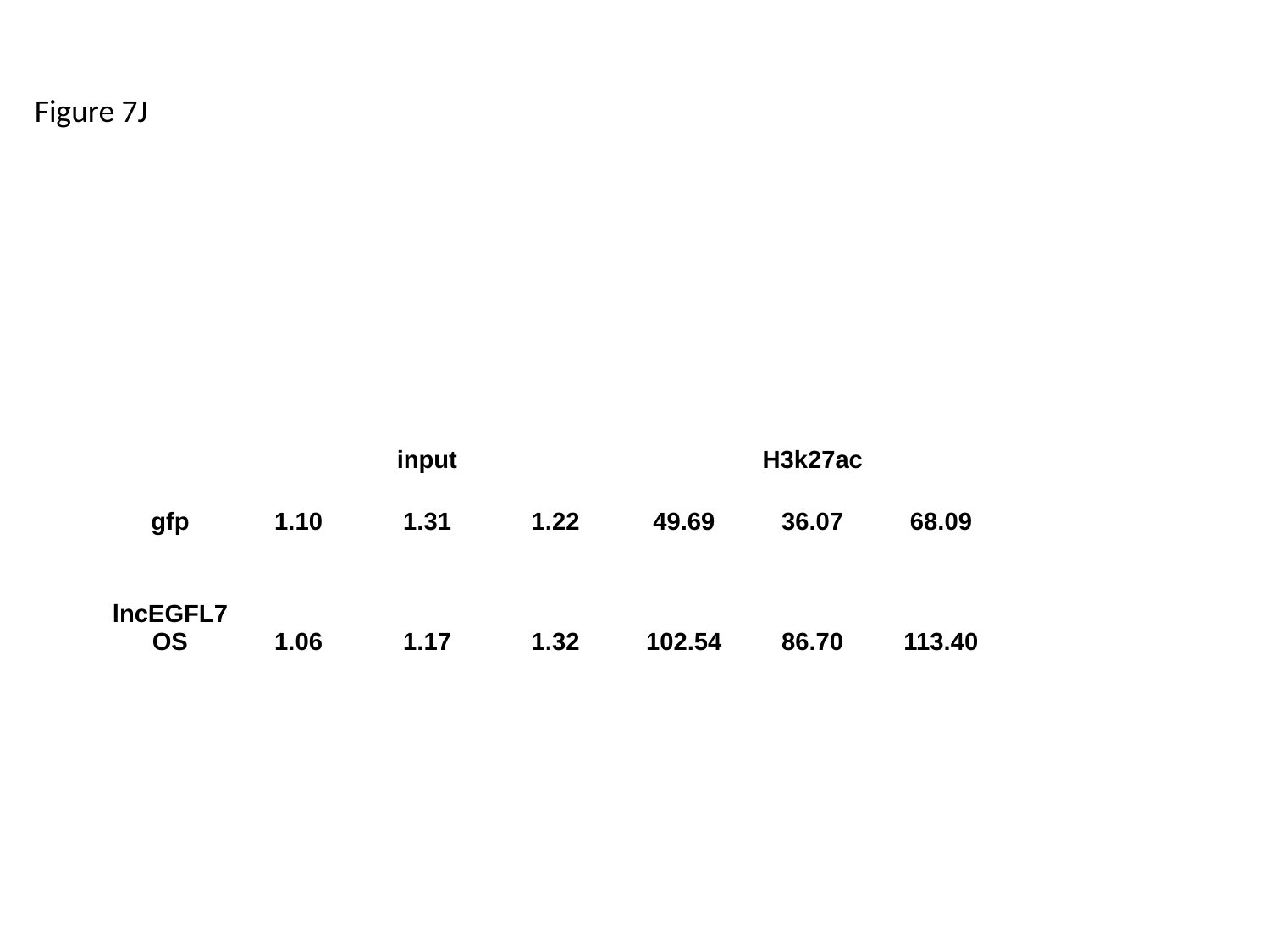

Figure 7J
| | input | | | H3k27ac | | |
| --- | --- | --- | --- | --- | --- | --- |
| gfp | 1.10 | 1.31 | 1.22 | 49.69 | 36.07 | 68.09 |
| lncEGFL7OS | 1.06 | 1.17 | 1.32 | 102.54 | 86.70 | 113.40 |

## Slide 11
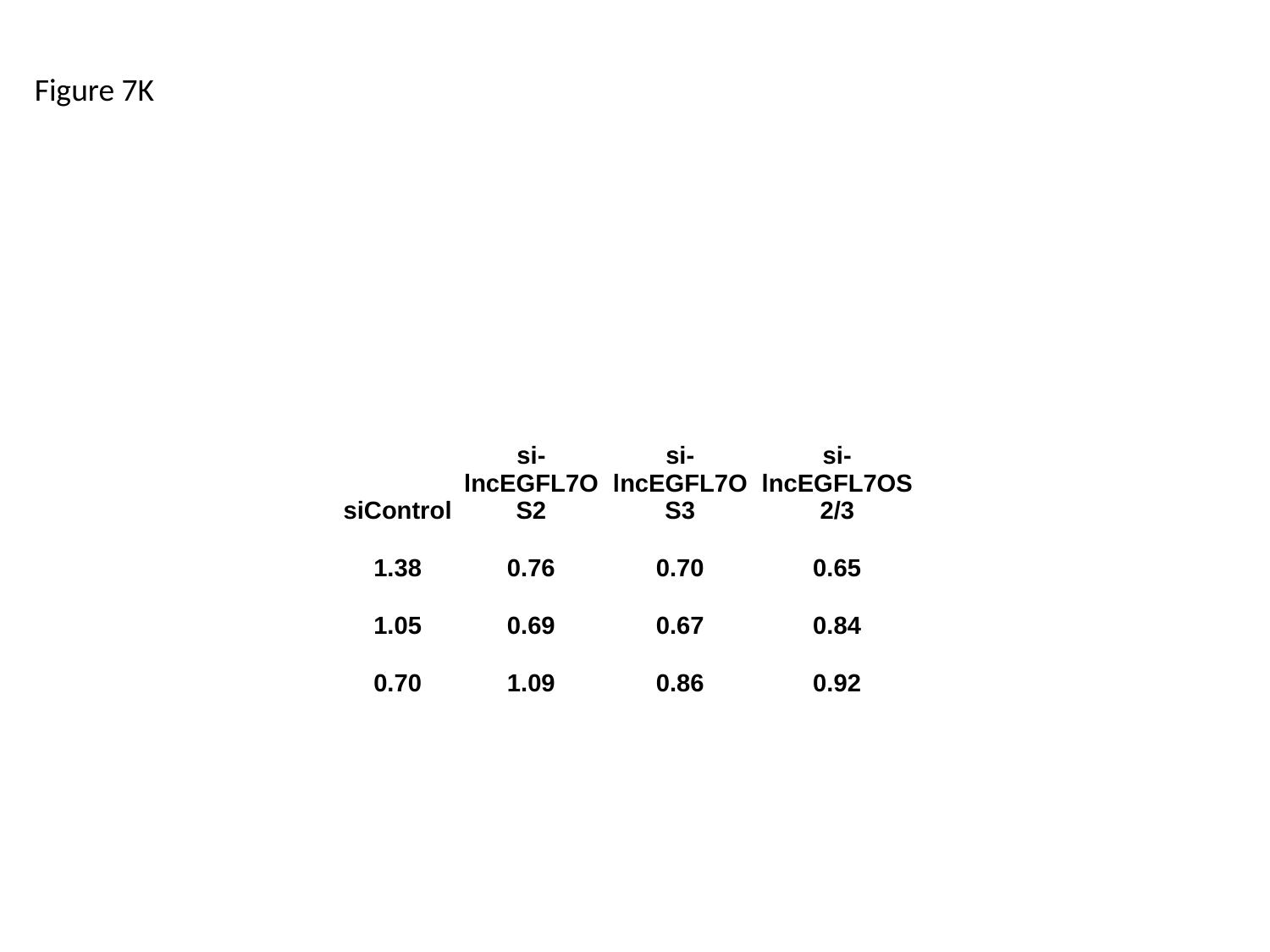

Figure 7K
| siControl | si-lncEGFL7OS2 | si-lncEGFL7OS3 | si-lncEGFL7OS2/3 |
| --- | --- | --- | --- |
| 1.38 | 0.76 | 0.70 | 0.65 |
| 1.05 | 0.69 | 0.67 | 0.84 |
| 0.70 | 1.09 | 0.86 | 0.92 |

## Slide 12
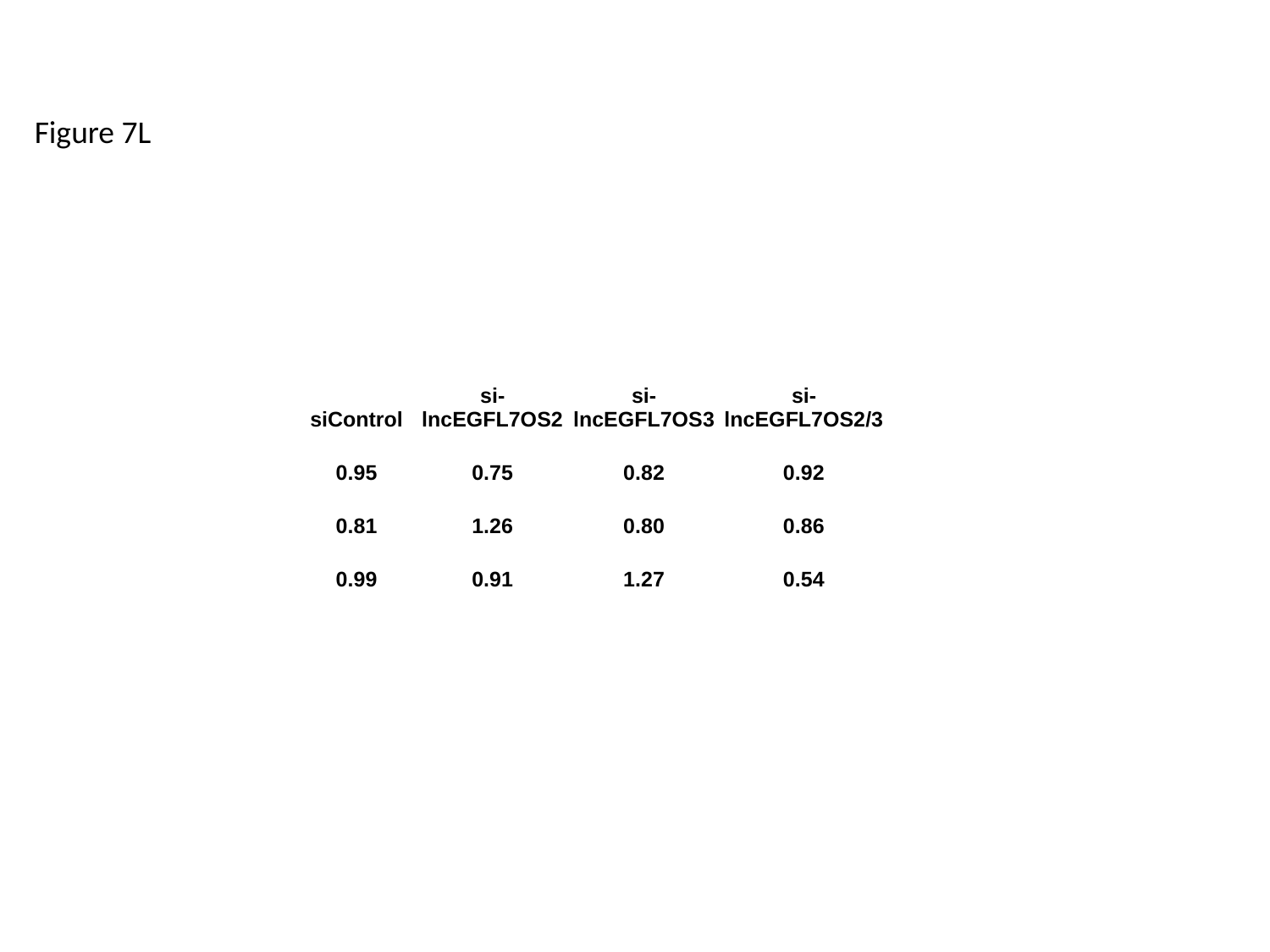

Figure 7L
| siControl | si-lncEGFL7OS2 | si-lncEGFL7OS3 | si-lncEGFL7OS2/3 |
| --- | --- | --- | --- |
| 0.95 | 0.75 | 0.82 | 0.92 |
| 0.81 | 1.26 | 0.80 | 0.86 |
| 0.99 | 0.91 | 1.27 | 0.54 |
