# Supplementary material for: LncEGFL7OS regulates human angiogenesis by interacting with MAX at the EGFL7/miR-126 locus
Source: eLife. 2019 Feb 11;8:e40470. doi: 10.7554/eLife.40470 (PMC6370342; doi:10.7554/eLife.40470)
Supplement: Supplementary file 1. [file elife-40470-supp1.docx]

**Supplementary File 1**

Topo 50 endothelial cell-enriched lncRNAs in ECs compare to non-ECs.

| lncRNA gene symbol | Probe name | Seq name | Fold  increase | P-value | Associated gene name | Normalized intensity (Log2 transformed) | | | | |
| --- | --- | --- | --- | --- | --- | --- | --- | --- | --- | --- |
|  |  |  |  |  |  | ARPE | HDEF | HCEC | HREC | HUVEC |
| SRGN | ASHGA5P018958 | NR_036430 | 255.3 | 0.0005 | SRGN | 5.0 | 5.9 | 14.0 | 13.4 | 13.0 |
| RP11-251M1.1 | ASHGA5P045551 | ENST00000411904 | 148.7 | 0.035 | EGFL7 | 4.0 | 2.5 | 7.5 | 11.9 | 11.9 |
| LOC158376 | ASHGA5P045709 | NR_024283 | 97.4 | 0.0003 |  | 5.6 | 6.1 | 12.4 | 12.9 | 12.1 |
| XLOC_000218 | ASHGA5P056199 | TCONS_00000959 | 47.4 | 0.0015 |  | 3.0 | 2.5 | 7.7 | 8.2 | 9.0 |
| AC147651.4 | ASHGA5P043463 | ENST00000429872 | 36.8 | 0.048 | PRKAR1B | 4.8 | 6.9 | 9.0 | 11.4 | 12.8 |
| XLOC_009769 | ASHGA5P049813 | TCONS_00020439 | 33.1 | 0.011 |  | 2.4 | 2.5 | 6.1 | 8.3 | 8.0 |
| RP11-463O9.5 | ASHGA5P031379 | ENST00000563280 | 30.9 | 0.031 | FOXC2 | 4.4 | 7.4 | 10.4 | 11.7 | 10.3 |
| SDHAP3 | ASHGA5P020537 | ENST00000515467 | 28.2 | 0.0018 |  | 4.2 | 4.5 | 9.4 | 9.5 | 8.5 |
| RP11-331F4.4 | ASHGA5P018983 | ENST00000463353 | 25.8 | 0.0187 |  | 2.4 | 4.0 | 6.9 | 9.1 | 7.7 |
| AC226119.4 | ASHGA5P020437 | ENST00000513501 | 25.3 | 0.015 |  | 2.4 | 4.9 | 8.0 | 8.5 | 8.4 |
| XLOC_009629 | ASHGA5P042127 | TCONS_00020285 | 24.0 | 0.008 |  | 3.4 | 2.5 | 7.2 | 6.8 | 8.5 |
| RP11-389C8.2 | ASHGA5P040497 | ENST00000564956 | 22.8 | 0.0198 |  | 2.9 | 2.5 | 7.9 | 5.7 | 8.1 |
| RP13-122B23.8 | ASHGA5P045573 | ENST00000566954 | 20.5 | 0.017 |  | 6.1 | 4.2 | 8.6 | 10.0 | 9.9 |
| XLOC_003195 | ASHGA5P031856 | TCONS_00006568 | 20.3 | 0.0057 |  | 6.3 | 5.5 | 10.1 | 11.0 | 9.6 |
| XLOC_009630 | ASHGA5P042126 | TCONS_00020286 | 19.6 | 0.0012 |  | 2.4 | 2.5 | 6.2 | 7.0 | 7.0 |
| RP11-160E2.11 | ASHGA5P032913 | ENST00000572818 | 18.4 | 0.0035 |  | 2.4 | 2.5 | 6.9 | 5.9 | 7.1 |
| LINC00520 | ASHGA5P029146 | ENST00000554221 | 18.3 | 0.0376 |  | 5.2 | 3.4 | 8.1 | 9.9 | 7.4 |
| RP13-317D12.3 | ASHGA5P047797 | ENST00000525893 | 17.3 | 0.003 | PTDSS2 | 11.4 | 12.5 | 16.4 | 16.1 | 15.7 |
| LOC100506178 | ASHGA5P043550 | NR_038393 | 17.1 | 0.0175 | STEAP1B | 2.4 | 2.5 | 5.2 | 7.1 | 7.3 |
| RP11-566K19.6 | ASHGA5P022492 | ENST00000560859 | 16.7 | 0.0054 |  | 6.2 | 6.6 | 11.2 | 10.4 | 9.8 |
| RP5-884M6.1 | ASHGA5P043843 | ENST00000470135 | 16.6 | 0.0092 |  | 6.0 | 4.5 | 8.8 | 9.4 | 9.8 |
| XLOC_009994 | ASHGA5P057682 | TCONS_00020693 | 15.7 | 0.0073 |  | 2.4 | 4.0 | 6.9 | 7.4 | 7.2 |
| AC002480.4 | ASHGA5P043551 | ENST00000439823 | 14.2 | 0.0367 | STEAP1B | 2.4 | 3.4 | 5.2 | 7.4 | 7.7 |
| XLOC_009994 | ASHGA5P042436 | TCONS_00020694 | 12.7 | 0.0177 |  | 3.7 | 3.8 | 6.3 | 8.4 | 7.6 |
| XLOC_011081 | ASHGA5P057881 | TCONS_00023134 | 11.8 | 0.0417 |  | 2.4 | 2.5 | 7.2 | 4.5 | 6.3 |
| LOC100505495 | ASHGA5P025862 | NR_040109 | 11.7 | 0.0126 |  | 2.4 | 2.5 | 5.9 | 6.9 | 5.2 |
| XLOC_014147 | ASHGA5P058304 | TCONS_00029753 | 11.7 | 0.0157 |  | 5.0 | 6.7 | 9.4 | 9.7 | 9.0 |
| FLI1-AS1 | ASHGA5P026051 | NR_038908 | 11.6 | 0.0103 | FLI1 | 9.2 | 10.4 | 13.9 | 13.0 | 13.0 |
| RP11-354K1.1 | ASHGA5P044232 | ENST00000412871 | 11.3 | 0.0059 |  | 5.3 | 4.2 | 8.7 | 8.0 | 8.0 |
| XLOC_004644 | ASHGA5P027842 | TCONS_00010837 | 11.3 | 0.0021 |  | 2.4 | 2.5 | 5.5 | 6.4 | 5.9 |
| AC116035.1 | ASHGA5P038731 | ENST00000450746 | 10.3 | 0.0486 |  | 3.5 | 4.6 | 6.0 | 7.9 | 8.4 |
| XLOC_001061 | ASHGA5P056325 | TCONS_00002502 | 10.2 | 0.0486 |  | 2.4 | 2.5 | 6.2 | 7.0 | 4.3 |
| AC156455.1 | ASHGA5P028338 | ENST00000535337 | 10.2 | 0.0484 |  | 5.5 | 7.8 | 9.1 | 10.7 | 10.1 |
| XLOC_005119 | ASHGA5P027069 | TCONS_00011631 | 9.9 | 0.0278 |  | 4.1 | 3.9 | 8.4 | 7.4 | 6.2 |
| XLOC_009994 | ASHGA5P057681 | TCONS_00020692 | 9.9 | 0.0401 |  | 2.4 | 4.9 | 6.7 | 7.2 | 7.0 |
| AC226119.4 | ASHGA5P020464 | ENST00000514073 | 9.8 | 0.0034 |  | 4.9 | 5.5 | 8.2 | 9.0 | 8.3 |
| LOC729987 | ASHGA5P050820 | NR_046088 | 9.6 | 0.0435 |  | 2.4 | 3.0 | 4.6 | 6.3 | 7.1 |
| RP11-536K7.3 | ASHGA5P018041 | ENST00000448685 | 9.5 | 0.0046 |  | 2.4 | 3.5 | 6.1 | 6.3 | 6.2 |
| XLOC_014369 | ASHGA5P058313 | TCONS_00029904 | 9.5 | 0.0057 |  | 2.4 | 2.5 | 5.1 | 5.7 | 6.3 |
| LOC100216479 | ASHGA5P015822 | NR_046259 | 9.1 | 0.0154 |  | 2.4 | 4.0 | 5.9 | 6.6 | 6.6 |
| ATP5SL | ASHGA5P025737 | NR_030765 | 9.1 | 0.014 | ATP5SL | 5.6 | 4.6 | 7.7 | 8.3 | 8.9 |
| BCRP2 | ASHGA5P051217 | ENST00000461808 | 8.9 | 0.0139 |  | 4.6 | 4.7 | 6.9 | 8.4 | 8.1 |
| BCRP2 | ASHGA5P051218 | ENST00000398241 | 8.8 | 0.0232 |  | 5.2 | 6.5 | 8.2 | 9.7 | 9.1 |
| RP1-302G2.5 | ASHGA5P042449 | ENST00000573382 | 8.7 | 0.0299 |  | 3.3 | 3.4 | 5.3 | 6.8 | 7.3 |
| XLOC_007615 | ASHGA5P057347 | TCONS_00016233 | 8.7 | 0.0179 |  | 4.5 | 4.7 | 6.7 | 8.1 | 8.4 |
| AX747766 | ASHGA5P032664 | uc001ben.1 | 8.1 | 0.0029 | ECE1 | 2.4 | 2.5 | 5.4 | 5.0 | 5.9 |
| XLOC_012861 | ASHGA5P058101 | TCONS_00026538 | 7.8 | 0.0247 |  | 2.42 | 3.2 | 5.2 | 6.8 | 5.4 |
