# Supplementary material for: LncEGFL7OS regulates human angiogenesis by interacting with MAX at the EGFL7/miR-126 locus
Source: eLife. 2019 Feb 11;8:e40470. doi: 10.7554/eLife.40470 (PMC6370342; doi:10.7554/eLife.40470)
Supplement: Supplementary file 3. [file elife-40470-supp3.docx]

| **Gene Symbol** | **Fold Change** | **Normalized Intensity (log2 transformed)** | | | | | **Associated Gene** | **Fold Change** | **Normalized Intensity (log2 transformed)** | | | | |
| --- | --- | --- | --- | --- | --- | --- | --- | --- | --- | --- | --- | --- | --- |
|  |  | **HUVEC** | **HREC** | **HCEC** | **ARPE** | **HDEF** |  |  | **HUVEC** | **HREC** | **HCEC** | **ARPE** | **HDEF** |
| RP11-291L15.2 | 66.6 | 12.3 | 11.6 | 9.9 | 8 | 2.5 | HHIP | 301.6 | 11.8 | 12 | 9.2 | 2.4 | 3.1 |
| HHIP-AS1 | 47.1 | 14 | 13.8 | 12.2 | 11.2 | 4.3 | HHIP | 301.6 | 11.8 | 12 | 9.2 | 2.4 | 3.1 |
| SRGN | 255.7 | 13 | 13.4 | 14 | 5 | 5.9 | SRGN | 160.3 | 13.2 | 13.9 | 14.8 | 6.3 | 7.1 |
| RP11-677M14.3 | 7.6 | 6.9 | 5.6 | 4.9 | 2.4 | 3.3 | ESAM | 22.5 | 10.7 | 10.2 | 6.5 | 4.4 | 4.9 |
| RP11-463O9.5 | 30.9 | 10.3 | 11.7 | 10.5 | 4.4 | 7.4 | FOXC2 | 13 | 5.4 | 6.8 | 7.9 | 3 | 3.1 |
| AC002480.4 | 14.2 | 7.7 | 7.4 | 5.2 | 2.4 | 3.5 | STEAP1B | 6.7 | 10.1 | 10.2 | 10 | 5 | 9.8 |
| LOC100506178 | 17.1 | 7.3 | 7.1 | 5.2 | 2.4 | 2.5 | STEAP1B | 6.7 | 10.1 | 10.2 | 10 | 5 | 9.8 |
| AC007255.8 | 5.9 | 5.2 | 6.5 | 7 | 3.8 | 3.6 | PRR15 | 6 | 5.3 | 5.5 | 8.1 | 4.2 | 3.2 |
| FN3KRP | 5 | 5.6 | 5.4 | 5.4 | 2.4 | 3.8 | FN3KRP | 5.4 | 6.1 | 5.7 | 5.9 | 2.4 | 4.5 |
| AX747766 | 8.1 | 5.9 | 5.1 | 5.4 | 2.4 | 2.5 | ECE1 | 5.1 | 8.2 | 8.2 | 7.7 | 5.6 | 5.7 |
| RP5-1007M22.2 | 3.8 | 4.8 | 5.3 | 6 | 3.1 | 3.8 | LRRC8B | 5 | 7.3 | 6.8 | 6.4 | 4.4 | 4.7 |
| NSUN2 | 4 | 4.4 | 4.3 | 4.7 | 2.4 | 2.5 | NSUN2 | 3.9 | 5.4 | 3.8 | 5.8 | 2.4 | 3.7 |
| AX748411 | 5.6 | 5.5 | 5.9 | 5.7 | 2.8 | 3.6 | EPN2 | 3.8 | 5.4 | 4.7 | 4.6 | 2.4 | 3.6 |
| UBE2L3 | 2.6 | 11.9 | 12.1 | 12 | 10.4 | 10.8 | UBE2L3 | 3.8 | 3.1 | 6.2 | 3.9 | 2.4 | 2.5 |
| FKBP1A-SDCBP2 | 5.3 | 5.7 | 6.5 | 5.9 | 3.3 | 4 | FKBP1A | 2.9 | 10.3 | 10.3 | 8.7 | 7.6 | 8.9 |
| AX747264 | 4.8 | 8.1 | 9 | 8.7 | 6.1 | 6.5 | THBD | 2.8 | 5.5 | 5.8 | 5.2 | 3.4 | 4.7 |
| RHBG | 3.7 | 4.1 | 4.4 | 4.5 | 2.4 | 2.5 | RHBG | 2.8 | 5.5 | 7.6 | 7.1 | 5.2 | 5.2 |
| RP5-1142A6.3 | 4.7 | 7.4 | 7.4 | 8.4 | 6.3 | 4.7 | MVD | 2.8 | 8.4 | 8.2 | 7.9 | 5.7 | 7.7 |
| RP11-410D17.2 | 8.6 | 6 | 9 | 5.8 | 4.3 | 3.4 | GOT2 | 2.5 | 11.3 | 11 | 11.6 | 9.8 | 10.2 |
| AX747737 | 3.6 | 3.9 | 4.1 | 4.9 | 2.4 | 2.5 | POLM | 2.5 | 5.2 | 6.2 | 5.7 | 4.6 | 4.2 |
| RBM18 | 5.6 | 6 | 7.3 | 7.2 | 4.6 | 4.2 | RBM18 | 2.4 | 9.9 | 9.7 | 9.7 | 8.2 | 8.9 |
| IKBKB | 4.2 | 4.5 | 4.6 | 4.4 | 2.4 | 2.5 | IKBKB | 2.3 | 4.8 | 3.6 | 4.1 | 2.4 | 3.6 |
| XLOC_001023 | 4 | 4 | 4.6 | 4.8 | 2.4 | 2.5 | ENSA | 2.3 | 5.5 | 5.3 | 5.9 | 4.3 | 4.4 |
| CREM | 3.9 | 7.7 | 7.9 | 7.9 | 5.6 | 6.2 | CREM | 2.1 | 6.5 | 7.3 | 6.4 | 6.2 | 5.2 |
| TTC19 | 4.9 | 4.9 | 5.4 | 5.6 | 3.5 | 2.5 | TTC19 | 2.1 | 5.7 | 5.7 | 6 | 4.8 | 4.8 |
| RP11-251M1.1 | 148.7 | 11.9 | 11.9 | 7.5 | 4 | 2.5 | EGFL7 | 2 | 5.1 | 5.9 | 4.7 | 4.2 | 4.3 |
| AC147651.4 | 36.8 | 12.8 | 11.4 | 9 | 4.8 | 6.9 | PRKAR1B | 2 | 5.3 | 6.9 | 6.1 | 4.8 | 5.4 |
| MIR22HG | 3.3 | 4.5 | 5.5 | 5.2 | 3.2 | 3.4 | WDR81 | 0.4 | 6.2 | 5.9 | 5.6 | 7.1 | 7.2 |
| MSTO1 | 5.2 | 5.1 | 5.7 | 3.6 | 2.4 | 2.5 | MSTO1 | 0.3 | 4.6 | 3.1 | 4.6 | 5.9 | 5.3 |
| RP11-455O6.2 | 4 | 4.2 | 4.9 | 4.2 | 2.4 | 2.5 | AZI1 | 0.3 | 2.5 | 2.7 | 3.2 | 3.6 | 5.5 |

Supplementary File 3. EC-enriched lncRNAs and their associated genes. Shown in the table are lncRNAs with more than 2 folds in ECs compared to non-ECs that have associated genes within 10kb of the lncRNA gene. Red indicates the lncRNA expression pattern is in parallel with the associated gene, and blue indicates inverse expression profile between lncRNA and its associated gene.
