# Supplementary material for: LncEGFL7OS regulates human angiogenesis by interacting with MAX at the EGFL7/miR-126 locus
Source: eLife. 2019 Feb 11;8:e40470. doi: 10.7554/eLife.40470 (PMC6370342; doi:10.7554/eLife.40470)
Supplement: Supplementary file 5. [file elife-40470-supp5.docx]

**Supplemental File 5**

LncEGFL7OS Stellaris FISH probe designed according to Stellaris FISH probe designer (<https://www.biosearchtech.com/Account/Login?return=/stellaris-designer>)

Probe Sequence (5’ to 3’) Probe Name

| ctggcactctgggcctgagc | lncEGFL7OS-probe_1 |
| --- | --- |
| aggctatgggatagggcaaa | lncEGFL7OS-probe_2 |
| gagtgcccctcctgtggtgg | lncEGFL7OS-probe_3 |
| ctccaggagcccaagagagt | lncEGFL7OS-probe_4 |
| cggctggctctgcctctgag | lncEGFL7OS-probe_5 |
| aaagttctccctcctgcact | lncEGFL7OS-probe_6 |
| agaacacaggacgtccacag | lncEGFL7OS-probe_7 |
| atgagggttctctgcgtctg | lncEGFL7OS-probe_8 |
| aagtgacctccccctcggtt | lncEGFL7OS-probe_9 |
| acacgccatctgtggattcg | lncEGFL7OS-probe_10 |
| ggaggcgctcgccatgcact | lncEGFL7OS-probe_11 |
| agggaacagtaagtgtgtcc | lncEGFL7OS-probe_12 |
| ggctggcgtctggccagagc | lncEGFL7OS-probe_13 |
| cggcgcgcacacacagggtc | lncEGFL7OS-probe_14 |
| aggcagctgcaaagagcagc | lncEGFL7OS-probe_15 |
| gtcttcgcaggaaccccttg | lncEGFL7OS-probe_16 |
| aggctcttccccaaggtgct | lncEGFL7OS-probe_17 |
| tgcgagttcaagttcagccg | lncEGFL7OS-probe_18 |
| cacaggtctgactcaggtag | lncEGFL7OS-probe_19 |
| tcttccgtagaggtgaaaaa | lncEGFL7OS-probe_20 |
| tgctagggaaacgctctgac | lncEGFL7OS-probe_21 |
| cagaagtaacttctaaaaca | lncEGFL7OS-probe_22 |
| tatacataacataatttgcc | lncEGFL7OS-probe_23 |
| ttaattctttaaaattatca | lncEGFL7OS-probe_24 |
| gggtttgagtaataattaca | lncEGFL7OS-probe_25 |
